# High quality of life, treatment tolerability, safety and efficacy in HIV patients switching from triple therapy to lopinavir/ritonavir monotherapy: A randomized clinical trial

**DOI:** 10.1371/journal.pone.0195068

**Published:** 2018-04-12

**Authors:** Juan Pasquau, Carmen Hidalgo-Tenorio, María Luisa Montes, Alberto Romero-Palacios, Jorge Vergas, Isabel Sanjoaquín, José Hernández-Quero, Koldo Aguirrebengoa, Francisco Orihuela, Arkaitz Imaz, María José Ríos-Villegas, Juan Flores, María Carmen Fariñas, Pilar Vázquez, María José Galindo, Isabel García-Mercé, Fernando Lozano, Ignacio de los Santos, Samantha Elizabeth de Jesus, Coral García-Vallecillos, on behalf of The QoLKAMON STUDY GROUP

**Affiliations:** 1 Hospital Universitario Virgen de las Nieves, Infectious Diseases, Granada, Spain; 2 Hospital Universitario de La Paz, Internal Medicine HIV Unit, Madrid, Spain; 3 Hospital Universitario de Puerto Real, Infectious Diseases, Cádiz, Spain; 4 Hospital Clínico San Carlos, Infectious Diseases, Granada, Spain; 5 Hospital Clínico Universitario Lozano Blesa, Infectious Diseases, Zaragoza, Spain; 6 Hospital Universitario San Cecilio, Infectious Diseases, Granada, Spain; 7 Hospital Universitario de Cruces, Infectious Diseases, Bilbao, Spain; 8 Hospital Regional Universitario de Málaga, Infectious Diseases, Málaga, Spain; 9 Hospital Universitario de Bellvitge, Infectious Diseases, Barcelona, Spain; 10 Hospital Universitario Virgen Macarena, Infectious Diseases and Clinical Microbiology, Seville, Spain; 11 Hospital Arnau de Vilanova, Infectious Diseases, Valencia, Spain; 12 Hospital Universitario Marqués de Valdecilla, Infectious Diseases, Santander, Spain; 13 Hospital Universitario Juan Canalejo, Infectious Diseases, La Coruña, Spain; 14 Hospital Clínico Universitario de Valencia, Infectious Diseases, Valencia, Spain; 15 Hospital General de L’Hospitalet, Infectious Diseases, Barcelona, Spain; 16 Hospital Universitario Nuestra Señora de Valme, Infectious Diseases, Seville, Spain; 17 Hospital Universitario de La Princesa, Infectious Diseases, Madrid, Spain; Imperial College London, UNITED KINGDOM

## Abstract

**Trial design:**

The QoLKAMON study evaluated quality of life, efficacy and treatment safety in HIV patients receiving lopinavir/ritonavir in monotherapy (MT) versus continuing combined antiretroviral triple treatment with a boosted protease inhibitor (TT).

**Methods:**

This was a 24-week, open-label, multicentre study in virologically-suppressed HIV-infected participants (N = 225) with a 2:1 randomization: 146 patients who switched to MT were compared with 79 patients who remained on a TT regimen. The primary endpoint was change in patient-reported outcomes in quality of life as measured by the MOS-HIV and EQ-5D questionnaires. Secondary endpoints included treatment adherence, patient satisfaction, incidence of adverse events and differences in plasma HIV-1 RNA viral load (VL) and CD4 cell counts.

**Results:**

Baseline quality of life, measured with the MOS-HIV score, was very good (overall score of 83 ± 10.5 in the MT arm and 82.3 ± 11.3 in the TT arm) and suffered no change during the study in any of the arms (at week 24, 83.5 ± 12.2 in MT arm and 81.9 ± 12.7 in TT arm), without statistically significant differences when compared. In regards to adherence to therapy and patient satisfaction, some aspects (number of doses forgotten in the last week and satisfaction of treatment measured with the CESTA score, dimension 1) improved significantly with MT. There were also no differences in the incidence and severity of adverse events, even though 22.8% of those in the MT arm switched their treatment when they were included in the study. Moreover, there was also no significant difference between the immunological and virological evolution of MT and TT. In the MT arm, the VL was always undetectable in 83% of patients (vs 90.7% in the TT arm) and there were only 6.7% of virological failures with VL > 50 copies/mL (vs 2.3% in the TT arm), without resistance mutations and with resuppression of VL after switching back to TT.

**Conclusions:**

In a new clinical trial, monotherapy as a treatment simplification strategy in HIV-1 infected patients with sustained viral suppression has demonstrated quality of life, safety and efficacy profiles comparable to those of conventional triple therapy regimens.

## Introduction

With the introduction of highly active antiretroviral therapy (HAART), HIV-1 infection has evolved from a fatal disease to a manageable chronic condition. Central goals of current HIV research are to avoid side effects associated with long-term therapy, promote patient comfort and compliance, and reduce economic costs. The standard treatment with three antiretroviral agents, usually two nucleoside/nucleotide reverse transcriptase inhibitors (NRTIs) plus either a boosted protease inhibitor (PI), an integrase inhibitor (II), or a non-nucleoside reverse transcriptase inhibitor (NNRTI) [1] is not exempt from risks, including the emergence of drug resistance and, principally, long-term toxicity. Some NRTIs can cause mitochondrial damage, which in turn, results in NRTI-related adverse events such as peripheral neuropathy, pancreatitis, liver disturbances, renal disease, lactic acidosis, bone mineral density loss or lipoatrophy [2, 3]. Furthermore, triple therapy (TT) regimens entail high economic costs and when co-formulation is unavailable or restricted, a less convenient regimen is necessary which may lead to low treatment adherence or dissatisfaction, which may potentially jeopardize treatment efficacy.

During the last decade, the use of simplification regimens [4], initiated once the patient has become stable and virologically suppressed, has become more attractive. Putative advantages of monotherapy (MT) strategies include decreases in toxicity, antiretroviral interactions, treatment complexity and cost, as well as preserving future treatment options, as the PIVOT study has recently shown [5]. Lopinavir/ritonavir (LPV/r) is a suitable candidate for single drug maintenance therapy due to its efficacy and high genetic barrier to resistance, which requires the accumulation of multiple mutations [6–9]. The emergence of resistance mutations in the protease gene are rare and have shown limited clinical significance [10–13]. Several clinical trials have addressed the efficacy of an LPV/r simplification strategy versus maintenance triple therapy [11, 14–19]. After 2 to 4 years of follow-up, different studies have shown that MT can maintain HIV viral suppression in a large proportion of patients [11, 19, 20]. MT is also the preferred salvage therapy for first line failures in developing countries as per WHO HIV treatment guidelines [21]. This has recently been challenged by the EARNEST study [22]. Furthermore, patients who experience viral load blips and emergence from suppression during MT can be safely reinduced with prior nucleosides without any apparent loss of therapeutic options, sparing other treatment classes for later use [11, 14–17, 20]. In Spain, as in the European EACS Guidelines, simplification to MT has been included since January 2008 in treatment guidelines as a therapeutic option for patients with no history of prior PI failure, undetectable viral load (VL) (<50 copies/mL) for at least six months, and signs and/or symptoms of toxicity from NRTIs [21]. In addition, pharmacoeconomic analysis of MT within the Spanish public health system was demonstrated to be a more affordable strategy than triple therapy [23–25].

However, poor treatment satisfaction is a significant risk factor for loss of viral control in patients undergoing antiretroviral therapy, including MT [11, 26]. To investigate the degree of patient compliance with this simplification treatment, we undertook a clinical trial that sought to measure the impact of two alternative regimens containing boosted PIs (MT vs TT) on patients’ quality of life, treatment satisfaction, compliance and tolerance, as well as virological efficacy.

The primary objective was to compare quality of life in patients who started MT with LPV/r versus patients continuing on TT containing a PI.

Secondary objectives were to compare MT vs TT with regard to treatment satisfaction, adherence, tolerability, regimen safety and virological and immunologic efficacy.

## Methods

### Study design

This was a phase IV national, open-label, multicentre, controlled, randomized (2:1), 24-week follow-up trial in HIV infected patients. The study was conducted at 31 different Spanish hospitals and was sponsored by the Sociedad Andaluza de Enfermedades Infecciosas (SAEI). The trial protocol was approved in advance by Ethics Committees from each participating hospital, and by a Reference Ethics Committee, representing all of them, the CCEIBA (Comité Coordinador de Ética de la Investigación Biomédica de Andalucía [Andalusian Biomedical Research Ethics Coordinating Committee]) in November 2009. It met the ethical principles specified in the Declaration of Helsinki and international good clinical practice guidelines, and was registered in December 2009 under EudraCT number 2009-014430-25. The authors confirm that all ongoing and related trials for this drug/intervention are registered. Written informed consent was obtained from each patient prior to any study procedures being performed. A pseudorandom number generator algorithm [27, 28] was used to prepare a randomisation list, which was held by a qualified contract research organization (CRO), not accessible to researchers, who were informed of the assigned treatment by telephone. Randomization was centralized and stratified by study centre, and each centre was regarded as a stratum. The size of the block was 4 to balance treatment arms of the global sample and within each centre. There was no further planned stratification by any other factor. The randomization list was centralized and hidden from researchers. Clinicians were informed of the treatment assigned to each patient by telephone consultation after they had confirmed, by fax, that the patient met all the inclusion and none of the exclusion criteria. The QoLKAMON trial is registered in ClinicalTrials.gov (number NCT01166477).

### Eligibility criteria

Patients over 18 years of age, with a positive HIV-1 antibody and/or PCR test, receiving any TT containing a boosted PI, and with undetectable VL (<50 viral RNA copies/mL) during the six months prior to enrolment were eligible for this study. For women with childbearing potential, a negative urine pregnancy test in the initial screening was mandatory. Patients were ineligible if any of the following conditions were present: pregnancy or nursing, acute hepatitis, documented resistance to LPV/r or failure on a PI therapy, concomitant therapy with drugs contraindicated for use with LPV/r, known history of drug addiction or chronic alcohol consumption, current active opportunistic infection or documented infection within 4 weeks of screening, renal disease with creatinine clearance <60 mL/min, concomitant use of nephrotoxic or immunosupressor drugs including corticosteroids, interleukin-2 or chemotherapy, prior medical history of psychiatric disorders such as depressive syndrome, schizophrenia or psychotic disease. Patients took Kaletra (lopinavir 200mg/ritonavir 50mg), 2 tablets bid. A protocol amendment modified the initial exclusion criterion (CD4 cell count nadir < 100 cells/μL) to allow any CD4 cell nadir, provided that the current CD4 count was > 250 cells/μL. In total, 46 patients had a nadir < 100 cells/μL.

### Outcome measurements

Patients’ overall quality of life was assessed using the Medical Outcomes Study HIV Health Survey (MOS-HIV) and EQ-5D questionnaires [29]. The MOS-HIV is a 35-item questionnaire that evaluates the following 10 dimensions of quality of life: general health perceptions, pain, physical functioning, role functioning, social functioning, mental health, energy/fatigue, health distress, cognitive functioning, and overall quality of life. Physical health summary (PHS) and mental health summary (MHS) scores are generated from some of the MOS-HIV dimensions, on a scale of 0–100, with higher scores indicating better health status. The EQ-5D is a 5-item instrument that measures mobility, self-care, usual activities, pain/discomfort and anxiety/depression. Index scores range from 0 to 100 where higher scores indicate better health. To measure adherence, a validated adherence questionnaire (from the GEEMA study) was employed [30], which includes 6 items recording multidimensional adherence measurements versus missed-dose measurements, the classification of adherence as a dichotomous (adherent/non-adherent) variable, and the time interval evaluated (weeks versus days). The visual analogue scale (VAS) is a subjective evaluation of the patient’s own level of compliance with the treatment and classifies adherence as a continuum variable, being 0 and 100 the worst and best health perceptions, respectively [31]. Patient satisfaction was evaluated using the CESTA questionnaire (Spanish Questionnaire of Satisfaction with Antiretroviral Treatment), which is an *ad-hoc* questionnaire developed to assess satisfaction in patients switching to a simplified regimen, addressing general health status, treatment, illness control, side effects, number of pills and doses, and dietary differences. This questionnaire has two dimensions, the first one related to the degree of treatment satisfaction and the second one to the assessment of factors determining patient satisfaction [32].

Safety was determined by the incidence of treatment adverse events, differences between arms in vital signs and clinical laboratory data, especially regarding HIV-1 RNA levels and CD4 cell counts. Adverse events were graded according to Common Toxicity Criteria [33]. Serious events were adverse events grade IV. Grade I events were classified as mild, grade II as moderate and grade III as severe. The relationship of adverse effects to treatment was based on the judgment of the physician.

The virological efficacy was assessed in different ways. Strict virological failure was defined by two consecutive measurements of plasma HIV-1 RNA > 500 copies/mL separated by at least two weeks. During virological failure, HIV genotyping was performed to determine resistance mutations. In the MT arm, virological rebounds were not considered as therapeutic failure if there was no evidence of LPV/r genotypic resistance and viral suppression could be reinduced with the addition of two NRTIs, leading to a subsequent decrease in VL of at least tenfold in four weeks and to < 50 copies/mL of HIV-1 RNA after 16 weeks. However, an ITT analysis in which analogous reintroduction meant a failure was also performed. The ability of the two strategies to maintain VL lower than 50 and 200 copies/mL was also assessed, and major virological efficacy was defined as undetectable VL during the whole follow-up period and at week 24.

### Study evaluations

Patients were assessed at baseline, week 12, and week 24. At each time point, the following were obtained: a clinical assessment, list of concomitant medications, adverse events, GEEMA, VAS and CESTA questionnaires, VL, CD4 cell count, liver enzymes, biochemistry and hematologic values. MOS-HIV and EQ-5D questionnaires were completed at the first and last assessments. An extra set of data was collected on week 4 for the experimental arm, that included VL and CD4 cell count, GEEMA, VAS, and CESTA questionnaires, and information on concomitant medications and adverse events.

### Statistical analysis

Sample size calculations for the current study were based on the expected difference between the two treatment arms. It was estimated, according to the literature [34], that in order to detect as statistically significant a relative improvement in quality of life of 10% in the experimental arm, with 80% power and a two-tailed significance level of 5%, a total of 390 fully evaluable patients (248 and 130 in each group) would be required. The study was discontinued by the sponsor after enrolment of 228 patients, due to a lower than estimated enrolment rate, and the final statistical power achieved was 59%. Treatment arms were compared using the Fisher’s exact test for categorical variables and the Student’s t-test and the non-parametric Wilcoxon or Mann-Whitney tests for continuous variables. The significance threshold was set at *P* = 0.05. Baseline characteristics of study groups were also compared using an analysis of variance (ANOVA). The main endpoint was ascertained from all the MOS-HIV scores and EQ-5D questionnaire outcomes. The odds ratio of virological rebound was calculated.

For all analyses, the primary population included all patients from the control arm and those from the experimental arm who received at least one dose of LPV/r (ITT: intention-to-treat population; n = 225). In some particular cases, such as MOS-HIV evaluation, an analysis of covariance (ANCOVA) was used for comparisons between both treatment arms, including baseline values, sex, and age as covariates.

## Results

**Fig 1:**
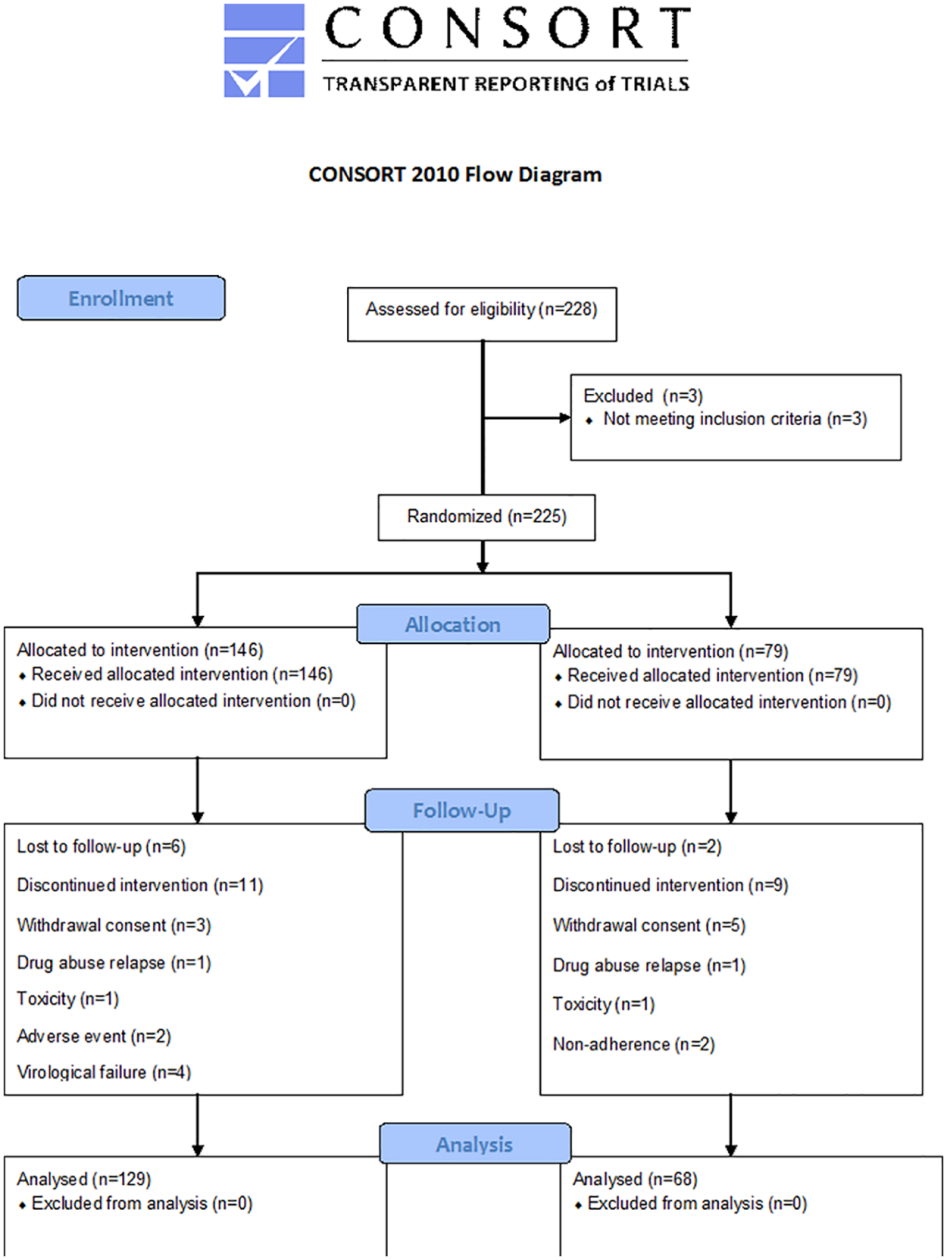
CONSORT flow diagram showing disposition of patients throughout the study from baseline to week 24.

Between February 2010 and June 2011, 225 patients were identified as eligible for the study and were randomized: 146 patients switched to MT and 79 patients remained on TT. At the beginning of the study, 77.2% of patients in the TT arm were taking lopinavir/ritonavir, while the remaining patients were taking fosamprenavir, saquinavir, atazanavir, darunavir, and nelfinavir. Furthermore, 90% of them were using two NRTIs, the most common being TDF/FTC (53%), followed by ABC/3TC (19%). Overall, 129 patients in the MT arm and 68 patients in the TT arm were still in the study at week 24 (Fig 1). Of the 79 randomized patients in the control arm, 5 withdrew consent to the study, 2 were lost to follow-up, 2 were non-adherent, 1 experienced toxicity and 1 had a drug abuse relapse. Of the 146 patients from the experimental arm, 6 were lost to follow-up, 3 withdrew consent, 2 had adverse events, 4 had virological failure, 1 suffered toxicity and 1 had a drug abuse relapse.

### Baseline characteristics

Baseline characteristics of the patients included in the study were not significantly different between the two groups, TT and MT, in the majority of measured parameters (Table 1). Mean age at screening was 45 years. Patients were predominantly male (71.4% and 71.8% respectively) with a mean time since HIV diagnosis of 12.8 ± 7 and 13.5 ± 7 years. At baseline, the mean CD4 cell count in both groups was > 600 cells/μL (720 ± 318 for the experimental arm and 611 ± 267 for the control arm, P < 0.01). As required per protocol, all patients had undetectable plasma HIV-1 RNA (< 50 copies/mL). A total of 73 patients in the MT arm and 39 in the TT arm were included with a CD4 nadir < 200 cells/μl; 28 patients in the MT arm and 18 in the TT arm had a nadir < 100 cells/μl.

**Table 1:** Patient demographics and baseline characteristics for the ITT population.

	MT	TT	P value
**Age (years)**			
Mean ± SD	44.5 ± 8	45.2 ± 9	0.745
**Gender**			
Male; N (%)	102 (71.8)	55 (71.4)	1
**Time from HIV diagnosis (years)**	13.5 ± 7	12.8 ± 7	0.587
**HIV treatment (years)**	8.83 ± 5.45	9.06 ± 5.32	0.637
**Risk factor for acquiring HIV** N (%)			
Homosexual	37 (25.3)	23 (29.1)	0.636
Heterosexual	39 (26.7)	27 (34.2)	0.283
Blood transfusion	1 (0.7)	1 (1.3)	1
IV drug user	66 (45.2)	28 (35.4)	0.202
Others	5 (3.4)	3 (3.8)	
**Absolute CD4+ count (cell/mm**^**3**^**)**			
Mean (SD)	719.99 ± 317.77	610.93 ± 266.88	0.01
CD4 (%)	30.87 ± 9.19	27.92 ± 9.14	0.027
**Viral load (RNA copies/mL)**			
Mean (SD)	26.85 ± 17.31	25.45 ± 11.99	0.306
**MOS-HIV overall score**			
Mean (SD)	83.0 ± 10.5	82.3 ± 11.3	
EQ-5D overall score			
Mean (SD)	81.8 ± 18.2	82.6 ± 15.7	

ITT, intent-to-treat; HIV, human immunodeficiency virus; MT, monotherapy; SD, standard deviation; TT, triple therapy.

### Quality of life evaluation

**Fig 2:**
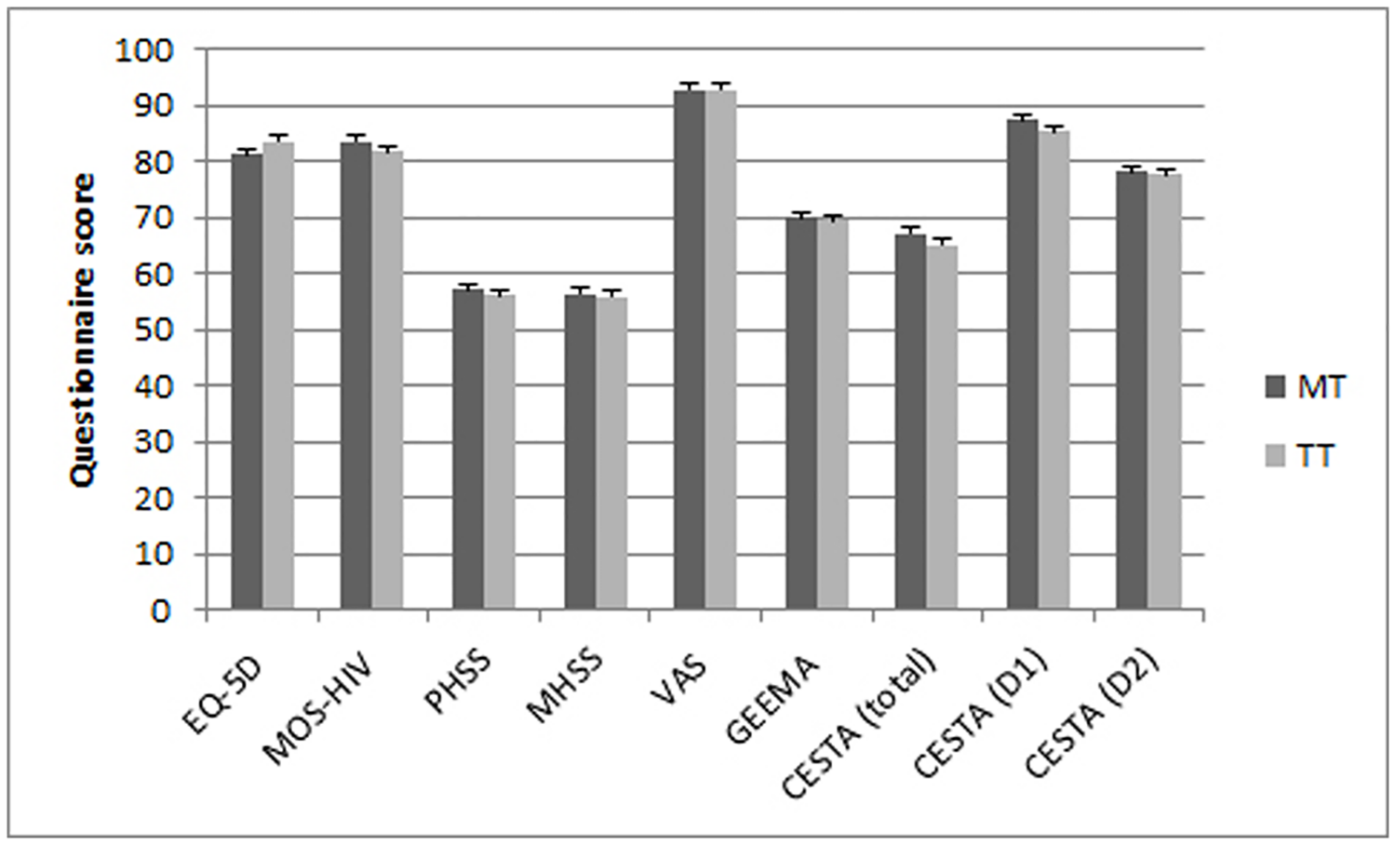
Final scores (mean values at week 24 with error bars showing 95% confidence intervals) of the 5 questionnaires evaluating the quality of life of both groups.

At baseline, the mean MOS-HIV overall scores were 83.0 ± 10.5 in the MT arm and 82.3 ± 11.3 in the TT arm (Table 1). An analysis of covariance (ANCOVA) was performed for comparisons between both treatment arms, including baseline values, sex, and age as covariates. There were no significant differences between treatment arms in mean MOS-HIV overall scores at week 24 (83.5 ± 12.2 vs 81.9 ± 12.7 for the experimental and control arms, respectively; mean difference: 1.6; CI 95%: -1.8–5.0; P = 0.366) (Fig 2). Moreover, regarding the physical and mental components of MOS-HIV, no significant differences were observed on the PHS (56.9 ± 6.5 and 56.3 ± 6.9; mean difference: 0.6; CI 95% -1.2–2.4; P = 0.356) and MHS (56.4 ± 7.2 and 56.4 ± 6.9; mean difference: 0.0; CI 95%: -2.0–2.0; P = 0.762) final scores between the experimental and control arms.

Concerning the EQ-5D questionnaire, the baseline scores were 81.8 ± 18.2 in the MT arm and 82.6 ± 15.7 in the TT arm (Table 1). The ANCOVA analysis showed that the EQ-5D final scores at week 24 were also similar (81.3 ± 21.9 and 83.5 ± 20.1; mean difference: -2.2 CI 95% -8.1–3.7; P = 0.704) betwee the control and experimental arms (Fig 2).

### Treatment adherence

Adherence rates (as GEEMA overall) were similar in both boosted PI-based regimens with no significant change over time (Fisher exact test at last visit, P = 1). At the beginning and end of the study, 74.1% and 70.1% of patients in the MT arm and 70% and 69.4% of patients in the TT arm were fully adherent to treatment, respectively. When all the items included in the GEEMA questionnaire were analysed separately, there were statistical differences only in the number of doses missed within the last week, which were more numerous in the TT arm (85.4% of the experimental arm did not miss any dose, versus 77.3% of the control arm, P = 0.05) (S1 Table). In the MT arm, the reason for not taking the pills was “forgetfulness” in 12.3% of the cases, and a similar figure was obtained for the TT arm (12.0%, P = 1.0; at last visit). There was no significant difference between the mean values of the VAS scale over time for subjects in the MT (92.8 ± 14.6 at last visit) and TT (92.9 ± 12.8 at last visit) arms nor between them (P = 0.197) (Fig 2).

### Patient satisfaction

Comparing both treatment arms, there was a significant difference in the mean score measuring the change in dimension 1 (3.9 ± 11.6 of MT vs 1.2 ± 8.8 of TT, P = 0.043), and specifically, a significantly greater satisfaction with the number of pills taken daily (P <0.001) among the patients in the monotherapy arm versus the TT arm (S2 Table).

### Safety and tolerability

There was no significant difference in the incidence of adverse events between treatment arms. Total and serious adverse events were reported in 17.8% and 4.1% of patients from MT versus 14.3% and 1.3% from TT (P = 0.573 and P = 0.426). The non-serious adverse events were mild in 81.1% and 58.8% in MT and TT arms respectively and considered unrelated to treatment in 83.8% and 82.4% of cases (Table 2).

**Table 2:** Patients with adverse events during lopinavir/ritonavir monotherapy compared with triple therapy, and number of adverse events according to grade severity.

**Patients**		**MT** **(n = 146)**		**TT** **(n = 76)**		
		**N**	**%**	**N**	**%**	**P (Fisher’s exact test)**
**Total of patients with adverse events**		26	17.8	11	14.3	0.573
**Patients with serious adverse events**		6	4.1	1	1.3	0.426
**Adverse events**		**MT** **(n = 45)**		**TT** **(n = 18)**		
**According to seriousness**	Serious adverse events (Grade IV)	8	17.8	1	5.6	0.426
	Non-serious adverse events (Grade I-III)	37	82.2	17	94.4	
**Number of non-serious adverse events according to intensity**	Mild (Grade I)	30	81.1	10	58.8	0.119
	Moderate (Grade II)	6	16.2	7	41.2	
	Severe (Grade III)	1	2.7	0	0	
**Number of non-serious adverse events according to causality**	Not related	31	83.8	14	82.4	0.275
	Possibly not related	1	2.7	2	11.8	
	Possibly related	4	10.8	0	0	
	Probably related	1	2.7	1	5.9	
**Number of non-serious adverse events according to action taken**	None	29	78.4	15	88.2	0.457
	Dose reduction	0	0	0	0	
	Temporary treatment interruption	1	2.7	0	0	
	Permanent treatment discontinuation	4	10.8	0	0	
	Others	3	8.1	2	11.8	

Per-protocol analysis.

MT, monotherapy; TT, triple therapy.

There were no significant differences for haematological parameters, vital signs and anthropometric measures over time either in each arm or between arms.

### Virological and immunologic responses

As to maintaining virological suppression, there were no significant differences between MT and TT arms. In the ITT analysis (where analogue introduction = failure), the percentages of patients with undetected VL (< 50 copies/mL) at any time during the study period were similar in both treatment arms (78.6% for MT vs 87% for TT; P = 0.148). Likewise, there was a similar percentage of subjects who, at the end of the study, had VL < 50 copies/mL (84.1% for MT vs 89.6% for TT, P = 0.313). No significant differences were found in the virological failure rate considering VL > 500 copies/mL (1.4% for MT vs 0% for TT, P = 0.546), VL > 200 copies/mL (3.4% for MT vs 0% for TT, P = 0.167), or VL > 50 copies/mL (8.2% for MT vs 3.9% for TT, P = 0.271), nor for the percentage of patients with VL < 200 copies/mL at any time during the study (91.8% for MT vs 96.2% for TT, P = 0.204). Furthermore, there were no statistical differences in adherence and virological failure between arms (Table 3).

**Table 3:** Comparison of adherence and virological failure between arms.

		**Adherent at the end of the follow-up period**				**P**
		**MT**		**TT**		
		N	%	N	%	
**Last VL undetectable** **(<50 copies/mL)**	No (> 50)	11	12.5	3	7.0	
	Yes (< 50)	77	87.5	40	93.0	0.337
**Undetectable VL** **during the study**	Yes	73	83.0	39	90.7	
	No	15	17.0	4	9.3	0.237
**Virological failure** **(>500 copies/mL)**	Yes	1	1.1	0	0.0	
	No	88	98.9	43	100.0	1.000
**Virological failure** **(>200 copies/ml)**	Yes	3	3.4	0	0.0	
	No	86	96.6	43	100.0	0.551
**Virological failure** **(>50 copies/mL)**	Yes	6	6.7	1	2.3	
	No	83	93.3	42	97.7	0.426
		**Non-adherent at the end of the follow-up period**				**P**
		**MT**		**TT**		
		N	%	N	%	
**Last VL undetectable** **(<50 copies/mL)**	No (> 50)	8	21.1	5	26.3	
	Yes (< 50)	30	78.9	14	73.7	0.655
**Undetectable VL** **during the study**	Yes	27	71.1	13	68.4	
	No	11	28.9	6	31.6	0.838
**Virological failure** **(>500 copies/mL)**	Yes	1	2.6	0	0.0	
	No	37	97.4	19	100.0	1.000
**Virological failure** **(>200 copies/ml)**	Yes	1	2.6	0	0.0	
	No	37	97.4	19	100.0	1.000
**Virological failure** **(>50 copies/mL)**	Yes	4	10.5	2	10.5	
	No	34	89.5	17	89.5	1.000

Intent-to-treat analysis.

Furthermore, the number of detectable VLs over time did not show significant differences (for example, 6.6% for MT vs 4.7% for TT, P = 0.253, for VL > 50 copies/mL). The risk of virological rebound was similar for both arms: the OR of MT versus TT was 1.429 (CI 95% 0.772–2.646) for VL > 50 copies/mL; and 1.762 (CI 95% 0.584–5.315) for VL > 200 copies/mL.

Genotype testing showed no LPV/r resistance in MT patients with virological failure. All of them regained virological suppression after restarting prior antiretroviral treatment.

Regarding the development of the immune response, although there was no statistical difference in total cell counts of CD4 cells/μl, the CD4 percentage of total lymphocytes showed a significant difference between arms (31.0 ± 8.7% [CI 24.6–37.0] vs 28.7 ± 10.0% [CI 22.0–35.0], for the MT and the TT arms, respectively; P = 0.039).

## Discussion

LPV/r monotherapy is one of the best known options of tailored therapeutic approaches for patients with persistently suppressed HIV viremia in which fewer than the recommended standard of three antiretrovirals are used [4]. The primary objective of this study was to measure the effect on HIV patients’ quality of life of simplifying conventional TT with an MT regimen. All patients reported high quality of life scores (over 80), independently of whether self-administered questionnaires were analysed as a total score or as separate items. After 24 weeks of follow-up, no statistical differences were observed in most of the quality of life indicators between patients treated with MT and those in whom TT was continued. Similar results were reported in the PIVOT trial for the mental and physical health summary scores [35].

It is well known that adherence is a determinant factor of antiretroviral treatment efficacy and, as such, it is an important variable that must be determined. High rates of adherence were observed in all patients. The GEEMA and VAS questionnaires did not show any statistical difference between the two arms.

The degree of patient satisfaction with the monotherapy regimen, measured by the dimension 1 of the CESTA questionnaire, was significantly greater than in the TT arm. This dimension registers satisfaction with general health status and treatment, disease control, absence of side effects, number of pills, dosage schedule, and dietary restrictions. This result could reflect the advantages of treatment simplification. Particularly, there was greater acceptance of the number of pills taken by the patients in the experimental arm.

In this study, there were no significant differences in adverse events and viral rebounds between treatment arms. In contrast with other studies, a low lymphocyte nadir (less than 200 or less than 100 cells/μl) was not shown to be a risk predictor for VL detection or for virological failure.

However, although the difference was not significant, TT has been shown to be more efficient than monotherapy in maintaining HIV-1 suppression, with fewer blips and viral rebounds [22]. As in other studies with PI regimens in monotherapy, detectable VL occurred in a small group of patients (less than 10%) with no consequences on the appearance of resistance to LPV/r, and in such a way that resuppression of HIV-1 replication is achieved after re-introduction of the previously withdrawn nucleoside backbone or any other additional antiretroviral treatment.

With regard to development of the immune response, the fact that the fraction of CD4/total lymphocytes increased to a greater extent in the TT arm has uncertain significance, and no conclusions can be drawn. Although an increase in the percentage of CD4 cells may be associated in some cases with a decrease in the percentage of CD8 cells, and thus with a decrease in immunological activation, no other activation parameters were measured to further evaluate this feature.

Almost 85% of ITT population in the MT arm had a VL of < 50 copies/mL at the end of the study. This outcome could be an indication that the monotherapy simplification strategy with LPV/r in stable, controlled HIV-1 patients is safe and efficient. While this conclusion is not novel, it reinforces results from previous studies [36] and supports a simplification strategy with boosted PI, as already proposed by other groups [37–41].

Previously, the MONARK trial had analysed quality of life as a secondary endpoint in antiretroviral-naive patients in MT [42], with the application of 1 questionnaire. The larger sample size and longer study period of that survey proved useful for evaluating whether MT provides a better quality of life than the TT regimen, which was shown true in case of the total number of symptoms, the number of symptoms causing discomfort, and the development in MT patients of a significant positive perception of their global health status.

The slight differences in patients’ treatment adherence, quality of life, and satisfaction shown by our study suggest that switching from standard TT to MT may be beneficial. However, a more obvious gain in quality of life was expected in the monotherapy arm, considering the simplification of the treatment, despite the twice-a-day administration. These results may also be due to limitations, such as a reduced statistical power due to sample size, the subjective nature of the assessment tools, short follow-up and cohort characteristics. Indeed, the study was carried out on patients who were already tolerating a stable antiretroviral regimen, with a high quality of life at baseline (VAS score over 80%), so the probability of showing differences was low *a priori*. Longer follow-up periods and a greater number of patients may be needed to demonstrate any change. There has also been a delay in the publication of this article due to the desire to elaborate on virological data outcomes. This prolonged the data collection period to perform a post-hoc analysis and rewrite the article for publication. This delay has led to a loss of interest in the study drug since the study was carried out. However, this study is important as it offers new data on the efficacy and safety of MT that could be extrapolated to darunavir.

In summary, we showed that both regimens exhibited high overall health scores, satisfaction and treatment adherence, and did not lead to significant differences from baseline in VL or CD4 cell counts. Our results are in general agreement with previous studies and support the effectiveness of LPV/r as a suitable LDR for some patients, in light of the evidence for maintained HIV-1 suppression and potential to avoid the side effects associated with nucleoside analogue-containing regimens. This strategy also preserves future treatment options in case of failure, and reduces the economic costs of antiretroviral therapy, while maintaining a very good quality of life.

## Supporting information

S1 Fig Members of the study group. (DOC)

S1 Table Treatment adherence, measured by the GEEMA questionnaire. Some of the questions from the last visit, for the ITT population.
(DOC)

S2 Table Treatment satisfaction, measured by the CESTA questionnaire. Questions from dimension 1 at last visit. Intent-to-treat analysis.
(DOC)

S1 File CONSORT checklist. (DOC)

S2 File QoLKAMON study protocol in Spanish. (PDF)

S3 File QoLKAMON study protocol in English. (PDF)

S4 File Dataset QoLKAMON AA. (XLSX)

S5 File Dataset QoLKAMON. (XLSX)

## References

[1] Thompson MA, Aberg JA, Hoy JF, Telenti A, Benson C, Cahn P, et al Antiretroviral treatment of adult HIV infection: 2012 recommendations of the International Antiviral Society-USA panel. JAMA. 2012;308(4):387–402. Epub 2012/07/24. doi: 10.1001/jama.2012.7961 . 22820792 10.1001/jama.2012.7961

[2] Leung GP. Iatrogenic mitochondriopathies: a recent lesson from nucleoside/nucleotide reverse transcriptase inhibitors. Adv Exp Med Biol. 2012;942:347–69. Epub 2012/03/09. doi: 10.1007/978-94-007-2869-1_16 . 22399431 10.1007/978-94-007-2869-1_16

[3] Falco V, Rodriguez D, Ribera E, Martinez E, Miro JM, Domingo P, et al Severe nucleoside-associated lactic acidosis in human immunodeficiency virus-infected patients: report of 12 cases and review of the literature. Clin Infect Dis. 2002;34(6):838–46. Epub 2002/02/19. doi: 10.1086/339041 . 11850865 10.1086/339041

[4] Ferretti F, Gianotti N, Lazzarin A, Cinque P. Central nervous system HIV infection in "less-drug regimen" antiretroviral therapy simplification strategies. Semin Neurol. 2014;34(1):78–88. Epub 2014/04/10. doi: 10.1055/s-0034-1372345 . 24715491 10.1055/s-0034-1372345

[5] Paton N SW, Arenas-Pinto A, Dunn D for the PIVOT Trial Team, editor Randomised Controlled Trial of a PI Monotherapy Switch Strategy for Long-term HIV Management (The PIVOT Trial). 21st Conference on Retroviruses and Opportunistic Infections (CROI) Paper number 550 LB; 2014 3–6 March; BOSTON.

[6] Walmsley S, Bernstein B, King M, Arribas J, Beall G, Ruane P, et al Lopinavir-ritonavir versus nelfinavir for the initial treatment of HIV infection. N Engl J Med. 2002;346(26):2039–46. Epub 2002/06/28. doi: 10.1056/NEJMoa012354 . 12087139 10.1056/NEJMoa012354

[7] Kempf DJ, King MS, Bernstein B, Cernohous P, Bauer E, Moseley J, et al Incidence of resistance in a double-blind study comparing lopinavir/ritonavir plus stavudine and lamivudine to nelfinavir plus stavudine and lamivudine. J Infect Dis. 2004;189(1):51–60. Epub 2004/01/01. doi: 10.1086/380509 . 14702153 10.1086/380509

[8] Chandwani A, Shuter J. Lopinavir/ritonavir in the treatment of HIV-1 infection: a review. Ther Clin Risk Manag. 2008;4(5):1023–33. Epub 2009/02/12. 19209283 10.2147/tcrm.s3285 PMC2621403

[9] Menendez-Arias L. Molecular basis of human immunodeficiency virus drug resistance: an update. Antiviral Res. 2010;85(1):210–31. Epub 2009/07/21. doi: 10.1016/j.antiviral.2009.07.006 . 19616029 10.1016/j.antiviral.2009.07.006

[10] Delaugerre C, Flandre P, Chaix ML, Ghosn J, Raffi F, Dellamonica P, et al Protease inhibitor resistance analysis in the MONARK trial comparing first-line lopinavir-ritonavir monotherapy to lopinavir-ritonavir plus zidovudine and lamivudine triple therapy. Antimicrob Agents Chemother. 2009;53(7):2934–9. Epub 2009/05/20. doi: 10.1128/AAC.01643-08 19451297 10.1128/AAC.01643-08 PMC2704639

[11] Arribas JR, Delgado R, Arranz A, Munoz R, Portilla J, Pasquau J, et al Lopinavir-ritonavir monotherapy versus lopinavir-ritonavir and 2 nucleosides for maintenance therapy of HIV: 96-week analysis. J Acquir Immune Defic Syndr. 2009;51(2):147–52. Epub 2009/04/08. doi: 10.1097/QAI.0b013e3181a56de5 . 19349870 10.1097/QAI.0b013e3181a56de5

[12] Wallis CL, Mellors JW, Venter WD, Sanne I, Stevens W. Protease Inhibitor Resistance Is Uncommon in HIV-1 Subtype C Infected Patients on Failing Second-Line Lopinavir/r-Containing Antiretroviral Therapy in South Africa. AIDS Res Treat. 2011;2011:769627 Epub 2011/04/15. doi: 10.1155/2011/769627 21490784 10.1155/2011/769627 PMC3066558

[13] Perez-Valero I, Arribas JR. Protease inhibitor monotherapy. Curr Opin Infect Dis. 2011;24(1):7–11. Epub 2010/12/15. doi: 10.1097/QCO.0b013e3283422cdf . 21150592 10.1097/QCO.0b013e3283422cdf

[14] Meynard JL, Bouteloup V, Landman R, Bonnard P, Baillat V, Cabie A, et al Lopinavir/ritonavir monotherapy versus current treatment continuation for maintenance therapy of HIV-1 infection: the KALESOLO trial. J Antimicrob Chemother. 2010;65(11):2436–44. Epub 2010/09/17. doi: 10.1093/jac/dkq327 . 20843990 10.1093/jac/dkq327

[15] Cameron DW, da Silva BA, Arribas JR, Myers RA, Bellos NC, Gilmore N, et al A 96-week comparison of lopinavir-ritonavir combination therapy followed by lopinavir-ritonavir monotherapy versus efavirenz combination therapy. J Infect Dis. 2008;198(2):234–40. Epub 2008/06/11. doi: 10.1086/589622 . 18540803 10.1086/589622

[16] Molto J, Santos JR, Negredo E, Miranda C, Videla S, Clotet B. Lopinavir/ritonavir monotherapy as a simplification strategy in routine clinical practice. J Antimicrob Chemother. 2007;60(2):436–9. Epub 2007/06/09. doi: 10.1093/jac/dkm198 . 17556354 10.1093/jac/dkm198

[17] Cahn P, Montaner J, Junod P, Patterson P, Krolewiecki A, Andrade-Villanueva J, et al Pilot, randomized study assessing safety, tolerability and efficacy of simplified LPV/r maintenance therapy in HIV patients on the 1 PI-based regimen. PLoS One. 2011;6(8):e23726 Epub 2011/09/03. doi: 10.1371/journal.pone.0023726 21886816 10.1371/journal.pone.0023726 PMC3158782

[18] Nunes EP, Santini de Oliveira M, Mercon M, Zajdenverg R, Faulhaber JC, Pilotto JH, et al Monotherapy with Lopinavir/Ritonavir as maintenance after HIV-1 viral suppression: results of a 96-week randomized, controlled, open-label, pilot trial (KalMo study). HIV Clin Trials. 2009;10(6):368–74. Epub 2010/02/06. doi: 10.1310/hct1006-368 . 20133267 10.1310/hct1006-368

[19] Avettand-Fenoel V, Flandre P, Chaix ML, Ghosn J, Delaugerre C, Raffi F, et al Impact of 48 week lopinavir/ritonavir monotherapy on blood cell-associated HIV-1-DNA in the MONARK trial. J Antimicrob Chemother. 2010;65(5):1005–7. Epub 2010/03/20. doi: 10.1093/jac/dkq084 . 20299496 10.1093/jac/dkq084

[20] Pulido F, Delgado R, Perez-Valero I, Gonzalez-Garcia J, Miralles P, Arranz A, et al Long-term (4 years) efficacy of lopinavir/ritonavir monotherapy for maintenance of HIV suppression. J Antimicrob Chemother. 2008;61(6):1359–61. Epub 2008/03/18. doi: 10.1093/jac/dkn103 . 18343802 10.1093/jac/dkn103

[21] Berenguer J, Pedrol PD, Polo R. Documento de consenso de Gesida/Plan Nacional sobre el Sida respecto al tratamiento antirretroviral en adultos infectados por el virus de la inmunodeficiencia humana 2002. http://www.gesida-seimc.org/pcientifica/fuentes/DcyRc/gesidadcyrc2012-Documentoconsenso-TAR-adulto-verordenador.pdf.

[22] Paton NI, Kityo C, Hoppe A, Reid A, Kambugu A, Lugemwa A, et al Assessment of second-line antiretroviral regimens for HIV therapy in Africa. N Engl J Med. 2014;371(3):234–47. Epub 2014/07/12. doi: 10.1056/NEJMoa1311274 . 25014688 10.1056/NEJMoa1311274

[23] Escobar I, Pulido F, Perez E, Arribas JR, Garcia MP, Hernando A. [Pharmacoeconomic analysis of a maintenance strategy with lopinavir/ritonavir monotherapy in HIV-infected patients]. Enferm Infecc Microbiol Clin. 2006;24(8):490–4. Epub 2006/09/22. . 16987465 10.1157/13092464

[24] Gonzalez Rivas L, Sanchez Gomez E, Sanchez del Moral R, Grutzmancher Saiz S, Pujol de la Llave E, Bocanegra Martin C. Simplification of antiretroviral therapy: a good choice for our patients and the sustainability of our health care system. Farm Hosp. 2011;35(6):317–21. Epub 2011/10/25. doi: 10.1016/j.farma.2011.01.003 . 22019116 10.1016/j.farma.2011.01.003

[25] Pasquau J, Gostkorzewicz J, Ledesma F, Anceau A, Hill A, Moecklinghoff C. Budget impact analysis of switching to darunavir/ritonavir monotherapy for HIV-infected people in Spain. Appl Health Econ Health Policy. 2012;10(2):139–41. Epub 2012/02/02. doi: 10.2165/11598380-000000000-00000 . 22293019 10.2165/11598380-000000000-00000

[26] Arribas JR, Doroana M, Turner D, Vandekerckhove L, Streinu-Cercel A. Boosted protease inhibitor monotherapy in HIV-infected adults: outputs from a pan-European expert panel meeting. AIDS Res Ther. 2013;10(1):3 Epub 2013/01/26. doi: 10.1186/1742-6405-10-3 23347595 10.1186/1742-6405-10-3 PMC3610245

[27] Wichmann BA, Hill ID. Algorithm AS 183: An efficient and portable pseudo-random number generator. Journal of the Royal Statistical Society Series C (Applied Statistics). 1982;31(2):188–90.

[28] McLeod AI. Remark AS R58: a remark on algorithm AS 183. An efficient and portable pseudo-random number generator. Journal of the Royal Statistical Society Series C (Applied Statistics). 1985;34(2):198–200.

[29] Wu AW, Hanson KA, Harding G, Haider S, Tawadrous M, Khachatryan A, et al Responsiveness of the MOS-HIV and EQ-5D in HIV-infected adults receiving antiretroviral therapies. Health Qual Life Outcomes. 2013;11:42 Epub 2013/03/19. doi: 10.1186/1477-7525-11-42 23497257 10.1186/1477-7525-11-42 PMC3602001

[30] Knobel H, Alonso J, Casado JL, Collazos J, Gonzalez J, Ruiz I, et al Validation of a simplified medication adherence questionnaire in a large cohort of HIV-infected patients: the GEEMA Study. AIDS. 2002;16(4):605–13. Epub 2002/03/02. . 11873004 10.1097/00002030-200203080-00012

[31] Giordano TP, Guzman D, Clark R, Charlebois ED, Bangsberg DR. Measuring adherence to antiretroviral therapy in a diverse population using a visual analogue scale. HIV Clin Trials. 2004;5(2):74–9. Epub 2004/04/30. doi: 10.1310/JFXH-G3X2-EYM6-D6UG . 15116282 10.1310/JFXH-G3X2-EYM6-D6UG

[32] Condes E, Aguirrebengoa K, Dalmau D, Estrada JM, Force L, Gorgolas M, et al [Validation of a questionnaire to estimate satisfaction with antiretroviral treatment: CESTA questionnaire]. Enferm Infecc Microbiol Clin. 2005;23(10):586–92. Epub 2005/12/06. . 16324547 10.1157/13081566

[33] Trotti A, Colevas AD, Setser A, Rusch V, Jaques D, Budach V, et al CTCAE v3.0: development of a comprehensive grading system for the adverse effects of cancer treatment. Semin Radiat Oncol. 2003;13(3):176–81. Epub 2003/08/07. doi: 10.1016/S1053-4296(03)00031-6 . 12903007 10.1016/S1053-4296(03)00031-6

[34] Lubeck DP, Fries JF. Changes in quality of life among persons with HIV infection. Qual Life Res. 1992;1(6):359–66. Epub 1992/12/01. . 1299468 10.1007/BF00704430

[35] Paton NI, Stohr W, Arenas-Pinto A, Fisher M, Williams I, Johnson M, et al Protease inhibitor monotherapy for long-term management of HIV infection: a randomised, controlled, open-label, non-inferiority trial. Lancet HIV. 2015;2(10):e417–26. Epub 2015/10/02. doi: 10.1016/S2352-3018(15)00176-9 . 26423649 10.1016/S2352-3018(15)00176-9 PMC4765553

[36] McKinnon JE, Arribas JR, Pulido F, Delgado R, Mellors JW. The level of persistent HIV viremia does not increase after successful simplification of maintenance therapy to lopinavir/ritonavir alone. AIDS. 2006;20(18):2331–5. Epub 2006/11/23. doi: 10.1097/QAD.0b013e32801189f6 . 17117019 10.1097/QAD.0b013e32801189f6

[37] Pulido F, Matarranz M, Rodriguez-Rivera V, Fiorante S, Hernando A. Boosted protease inhibitor monotherapy. What have we learnt after seven years of research? AIDS Rev. 2010;12(3):127–34. Epub 2010/09/16. 20842201

[38] Molto J, Clotet B. [Lopinavir/ritonavir monotherapy as a simplification strategy in antiretroviral therapy in clinical practice]. Enferm Infecc Microbiol Clin. 2008;26 Suppl 16:24–6. Epub 2009/07/03. . 19572441 10.1016/s0213-005x(08)76607-2

[39] Lopez Aldeguer J. [Lopinavir/ritonavir monotherapy. Possible indications]. Enferm Infecc Microbiol Clin. 2008;26 Suppl 16:21–3. Epub 2009/07/03. . 19572440 10.1016/s0213-005x(08)76606-0

[40] Pulido Ortega F, Llenas-Garcia J. [Lopinavir/ritonavir monotherapy as a simplification strategy in the treatment of HIV-1 infection]. Enferm Infecc Microbiol Clin. 2008;26 Suppl 16:12–20. Epub 2009/07/03. . 19572439 10.1016/s0213-005x(08)76605-9

[41] Calza L, Manfredi R. Protease inhibitor monotherapy as maintenance regimen in patients with HIV infection. Curr HIV Res. 2012;10(8):661–72. Epub 2012/09/29. . 23016538 10.2174/157016212803901419

[42] Spire B, Marcellin F, Cohen-Codar I, Flandre P, Boue F, Dellamonica P, et al Effect of lopinavir/ritonavir monotherapy on quality of life and self-reported symptoms among antiretroviral-naive patients: results of the MONARK trial. Antivir Ther. 2008;13(4):591–9. Epub 2008/08/05. . 18672538

