# Supplementary material for: High quality of life, treatment tolerability, safety and efficacy in HIV patients switching from triple therapy to lopinavir/ritonavir monotherapy: A randomized clinical trial
Source: PLoS One. 2018 Apr 12;13(4):e0195068. doi: 10.1371/journal.pone.0195068 (PMC5896909; doi:10.1371/journal.pone.0195068)
Supplement: S1 Fig — (DOC) [file pone.0195068.s001.doc]

Other members of **The Qolkamon Study Group** are:

Antonio Vergara Campos (Hospital Universitario de Puerto Real, Infectious Diseases, Cádiz, Spain); Daniel Podczamcer (Hospital de Bellvitge, Barcelona); Jesús Rodríguez Baño (Hospital Universitario Virgen de Macarena, Sevilla), Julio César Blázquez (Hospital de Torrevieja, Alicante, Spain); Rafael Torres (Hospital Universitario Severo Ochoa, Leganés, Madrid, Spain); José María Cuadrado (Hospital Universitario San Juan, Alicante, Spain); Carmen Gálvez (Hospital Torrecárdenas, Almería, Spain); María del Mar Alonso (Hospital Universitario de Canarias, Tenerife, Spain); Jesús Santos (Hospital Universitario Virgen de la Victoria, Málaga, Spain); Juan Berenguer (Hospital General Univesitario Gregorio Marañón, Madrid, Spain); Francisco Pasquau (Hospital Marina Baixa, Villajoyosa, Alicante, Spain); Jaime Locutura (Hospital General Yagüe, Burgos, Spain); Juan Miguel Santamaría (Hospital Universitario Basurto, Bilbao, Spain), Carlos Armiñanzas (Hospital Universitario Marqués de Valdecillas, Santander, Spain), Manuel Gutiérrez-Cuadra (Hospital Universitario Marqués de Valdecillas, Santander, Spain), Arturo Prieto (Complejo Hospitalario Universitario de Santiago, La Coruña, Spain) and Miguel Ángel Cárdenes (Hospital Universitario de Gran Canaria Doctor Negrín, Las Palmas de Gran Canaria, Spain).
