# Supplementary material for: High quality of life, treatment tolerability, safety and efficacy in HIV patients switching from triple therapy to lopinavir/ritonavir monotherapy: A randomized clinical trial
Source: PLoS One. 2018 Apr 12;13(4):e0195068. doi: 10.1371/journal.pone.0195068 (PMC5896909; doi:10.1371/journal.pone.0195068)
Supplement: S1 Table — Some of the questions from the last visit, for the ITT population. (DOC) [file pone.0195068.s002.doc]

**S1 Table.** Treatment adherence, measured by the GEEMA questionnaire. Some of the questions from the last visit, for the ITT population.

|  |  | **MT (N=144) %** | **TT (N=75)%** | **P (Fisher’s exact test)** |
| --- | --- | --- | --- | --- |
| **Do you forget to take the pills?** | **Yes** | 17.8 | 18.9 | 0.855 |
|  | **No** | 82.2 | 81.1 |  |
| **Do you take the pills at the stipulated time?** | **Yes** | 91.8 | 97.3 | 0.147 |
|  | **No** | 8.2 | 2.7 |  |
| **Do you stop taking your medication if you feel sick?** | **Yes** | 2.1 | 6.9 | 0.124 |
|  | **No** | 97.9 | 93.1 |  |
| **Number of missed doses during last week** | **0** | 85.4 | 77.3 | 0.05 |
|  | **1-2** | 13.9 | 14.7 |  |
|  | **3-5** | 0.7 | 2.7 |  |
|  | **6-10** | 0 | 4 |  |
|  | **>10** | 0 | 1.3 |  |
| **Do you forget to take the pills during the weekend?** | **Yes**  **No** | 6.1  93.9 | 8.8  91.2 | 0.561 |

ITT, intent-to-treat; MT, monotherapy; TT, triple therapy.
