# Supplementary material for: High quality of life, treatment tolerability, safety and efficacy in HIV patients switching from triple therapy to lopinavir/ritonavir monotherapy: A randomized clinical trial
Source: PLoS One. 2018 Apr 12;13(4):e0195068. doi: 10.1371/journal.pone.0195068 (PMC5896909; doi:10.1371/journal.pone.0195068)
Supplement: S2 Table — Questions from dimension 1 at last visit. Intent-to-treat analysis. (DOC) [file pone.0195068.s003.doc]

**S2 Table.** Treatment satisfaction, measured by the CESTA questionnaire. Questions from Dimension 1 at last visit. Intent-to-treat analysis.

| **Dimension 1** |  | **MT** | | **TT** | | **P (Fisher’s exact test)** |
| --- | --- | --- | --- | --- | --- | --- |
| Degree of satisfaction concerning: |  | N | % | N | % |  |
| General health condition | Deeply unsatisfied | 2 | 1.5 | 0 | 0 | 0.767 |
|  | Quite unsatisfied | 1 | 0.7 | 0 | 0 |  |
|  | Regular | 6 | 4.4 | 4 | 5.4 |  |
|  | Quite satisfied | 52 | 38 | 30 | 40.5 |  |
|  | Deeply satisfied | 76 | 55.5 | 40 | 54.1 |  |
| Disease control | Deeply unsatisfied | 2 | 1.5 | 0 | 0 | 0.444 |
|  | Quite unsatisfied | 0 | 0 | 0 | 0 |  |
|  | Regular | 4 | 2.9 | 4 | 5.5 |  |
|  | Quite satisfied | 43 | 31.4 | 27 | 37 |  |
|  | Deeply satisfied | 88 | 64.2 | 42 | 57.5 |  |
| Lack of secondary effects or discomfort | Deeply unsatisfied | 3 | 2.2 | 0 | 0 | 0.663 |
|  | Quite unsatisfied | 1 | 0.7 | 1 | 1.4 |  |
|  | Regular | 12 | 8.8 | 7 | 9.6 |  |
|  | Quite satisfied | 49 | 35.8 | 30 | 41.1 |  |
|  | Deeply satisfied | 72 | 52.6 | 35 | 47.9 |  |
| Amount of pills taken daily | Deeply unsatisfied | 0 | 0 | 5 | 6.8 | <0.001 |
|  | Quite unsatisfied | 0 | 0 | 1 | 1.4 |  |
|  | Regular | 23 | 16.8 | 30 | 41.1 |  |
|  | Quite satisfied | 64 | 46.7 | 28 | 38.4 |  |
|  | Deeply satisfied | 50 | 36.5 | 9 | 12.3 |  |
| Number of times per day that you take medication | Deeply unsatisfied | 1 | 0.7 | 0 | 0 | 0.483 |
|  | Quite unsatisfied | 0 | 0 | 1 | 1.4 |  |
|  | Regular | 24 | 17.5 | 17 | 23.3 |  |
|  | Quite satisfied | 69 | 50.4 | 34 | 46.6 |  |
|  | Deeply satisfied | 43 | 31.4 | 21 | 28.8 |  |
| Dietary changes or restrictions related to treatment | Deeply unsatisfied | 0 | 0 | 0 | 0 | 0.802 |
|  | Quite unsatisfied | 0 | 0 | 0 | 0 |  |
|  | Regular | 8 | 5.8 | 6 | 8.1 |  |
|  | Quite satisfied | 57 | 41.6 | 29 | 39.2 |  |
|  | Deeply satisfied | 72 | 52.6 | 39 | 52.7 |  |
| General degree of satisfaction with treatment received | Deeply unsatisfied | 2 | 1.5 | 0 | 0 | 0.246 |
|  | Quite unsatisfied | 0 | 0 | 0 | 0 |  |
|  | Regular | 5 | 3.7 | 6 | 8.1 |  |
|  | Quite satisfied | 57 | 42.2 | 36 | 48.6 |  |
|  | Deeply satisfied | 71 | 52.6 | 32 | 43.2 |  |

MT, monotherapy; TT, triple therapy.
