# Supplementary material for: High quality of life, treatment tolerability, safety and efficacy in HIV patients switching from triple therapy to lopinavir/ritonavir monotherapy: A randomized clinical trial
Source: PLoS One. 2018 Apr 12;13(4):e0195068. doi: 10.1371/journal.pone.0195068 (PMC5896909; doi:10.1371/journal.pone.0195068)
Supplement: S2 File — (PDF) [file pone.0195068.s005.pdf]

PROTOCOLO DE ENSAYO CLINICO

Ensayo clínico, abierto, aleatorizado para comparar la calidad de vida de los pacientes VIH+ que inician monoterapia con comprimidos de LPV/r vs triple terapia que contenga un IP potenciado. Estudio QoLKAMON.

Promotor: **SAEI**

Codigo de Protocolo: **SAI-CDV-2009-01**

Código EudraCT: **2009-014430-25**

Versión Final: **1.1 (modificación de la versión inicial del 24 de Julio de 2009)**

RESUMEN

Identificación del promotor:	SAEI<br>Sociedad Andaluza de Enfermedades Infecciosas
Título del ensayo clínico:	Ensayo clínico abierto, aleatorizado, para comparar la calidad de vida de los pacientes VIH+ que inician monoterapia con comprimidos de LPV/r vs triple terapia que contengan un IP potenciado.
Código del protocolo:	SAI-CDV-2009-01
Código EudraCT:	2009-014430-25
Monitorización del Estudio:	Effice spi S.L.<br>c/ Capitán Haya, 51 4º5<br>28020 Madrid
Fase del ensayo clínico:	Fase IV
Objetivos	Objetivo principal: comparar la calida de vida de pacientes que inicien monoterapia con comprimidos de lopinavir/ritonavir® (LPV/r) vs aquellos en triple terapia que contengan cualquier Inhibidor de la Proteasa potenciado (IP).<br>Objetivos secundarios:<br>1. Evaluación de la eficacia virológica de la monoterapia basada en comprimidos de LPV/r vs triple terapia que contengan cualquier IP.<br>2. Evaluación de los cambios en la respuesta inmune en los pacientes VIH+ que inician monoterapia con comprimidos de LPV/r vs triple terapia que contengan cualquier IP.<br>3. Evaluación de la satisfacción del paciente con el

tratamiento de los pacientes VIH+ que inician monoterapia con comprimidos de LPV/r vs triple terapia que contengan cualquier IP.

4. Evaluación de la adherencia al tratamiento de los pacientes VIH+ que inician monoterapia con comprimidos de LPV/r vs triple terapia que contengan cualquier IP.
5. Evaluación de la tolerabilidad y seguridad de las pautas . en los pacientes VIH+ que inician monoterapia con comprimidos de LPV/r vs triple terapia que contengan cualquier IP.

Diseño:	Ensayo clínico Fase IV, nacional, multicéntrico, controlado, aleatorizado, abierto, comparativo de grupos paralelos
Proceso de aleatorización	Se realizará de forma centralizada
Grupos de tratamiento	Tratamiento:<br>Los pacientes se aleatorizarán, en relación 1:2, en dos grupos A y B, respectivamente.<br><br>Grupo control (A): Los pacientes continuarán la triple terapia antirretroviral basada en cualquier IP potenciado con ritonavir<br><br>Grupo experimental (B): Cambiar la triple terapia antirretroviral basada en cualquier IP potenciado con ritonavir a LPV/r monoterapia.<br><br>La aleatorización se realizará centralizadamente.
Enfermedad en estudio:	Infección por VIH.
Variable principal de valoración:	Calida de vida, medida por los cuestionarios MOS-VIH y EQ-5D, de pacientes que inicien monoterapia con comprimidos de lopinavir/ritonavir (LPV/r) vs aquellos en triple terapia que contengan cualquier Inhibidor de la

	Proteasa potenciado (IP).
Número de sujetos:	390 (260 en el grupo experimental B y 130 en el grupo control A)
Criterios de selección	Criterios de Inclusión:

- Pacientes infectados por VIH-1 documentada por prueba de anticuerpos frente a VIH-1 positiva y/o PCR positiva confirmadas para ARN del VIH-1
- Pacientes en triple terapia antirretroviral que contenga cualquier IP potenciado.
- Pacientes con una carga viral ARN VIH indetectable, definida como < 50 copias/mL, en los últimos 6 meses.
- Varones o mujeres de edad ≥ 18 años.
- Para mujeres en edad fértil, prueba de embarazo en orina negativa en la visita de selección.
- Pacientes que hayan otorgado el consentimiento informado por escrito antes de realizar cualquier procedimiento de selección específico del estudio.

Criterios de exclusión:

- Pacientes con constancia documentada, en el genotipado acumulado, de mutaciones de resistencia que confieran pérdida de sensibilidad a LPV/r® ó, en ausencia de genotipado, la existencia de pruebas de haber fracasado a una terapia con Inhibidores de la Proteasa (IP).
- Pacientes con un nadir de células CD4 <100 cel/μL.
- Pacientes que por cualquier motivo no puedan ser tratados con LPV/r®.
- Antecedentes de desórdenes psiquiátricos como Síndrome Depresivo, Esquizofrenia o enfermedad Psicótica.
- Antecedentes conocidos de drogadicción o consumo crónico de alcohol que, en opinión del investigador,

contraindican su participación en el estudio.

- Mujeres embarazadas o en período de lactancia o mujeres en edad fértil que no utilizan un método anticonceptivo adecuado según el criterio del investigador.
- Infección oportunista activa actual o infección documentada en el plazo de 4 semanas anteriores a la selección.
- Pacientes a los que por toxicidades graves relacionados con cualquier componente de la triple terapia antirretroviral actual se tenga previsto modificar o retirar cualquiera de los componentes de dicha terapia.
- Pacientes en los que los investigadores consideren que se debe cambiar la medicación antirretroviral, por cualquier causa o motivo, en los próximos 6 meses.
- Enfermedad renal con aclaración de creatinina <60 mL/min.
- Uso concomitante de fármacos contraindicados con LPV/r® como rifampicina, dihidroergotamina, ergotamina, metilergonovina, cisapride, hipericum perforatum, lovastatina, simvastatina, pimozide, midazolam y triazolam.
- Uso concomitante de fármacos nefrotóxicos o inmunosupresores.
- Paciente en tratamiento actual con corticoesteroides sistémicos, interleucina-2 o quimioterapia.
- Pacientes en tratamiento con otros agentes en investigación.
- Pacientes con hepatitis aguda y con enfermedad hepática avanzada (cirrosis descompensada).
- Cualquier enfermedad que, a juicio del investigador, contraindique la participación del paciente en el estudio.

Duración del tratamiento:	24 semanas.
Calendario y fecha prevista de finalización:	La duración aproximada del reclutamiento es de 6 meses. La duración del tratamiento es de 24 semanas<br>Inicio del reclutamiento: Noviembre 2009.<br>Fin de reclutamiento: Junio 2010.<br>Fin de seguimiento del último paciente: Diciembre 2010.<br>Informe final: Marzo 2011.

GLOSARIO ABREVIATURAS

AA	Acontecimientos Adversos
AAG	Acontecimientos Adversos Graves
AEMPS	Agencia Española del Medicamento y Productos Sanitarios
ART	Tratamientos Antirretrovirales
AZT	Zidovudina
AZT/3TC	Combivir
CV	Calidad de Vida
CEIC	Comité Ético de Investigación Clínica
CRD	Cuaderno de Recogida de Datos
CRO	Clinical Research Organization
EVA	Escala Visual Analógica
EQ-5D	EuroQol 5-D
3TC	Emtricitabina
IP	Inhibidores de la Proteasa
ITIAN	Inhibidores de la transcriptasa inversa análogos de nucleósidos
ITINN	Inhibidores de la transcriptasa inversa no análogos de nucleósidos
LPV/r	Lopinavir/ritonavir
MOS_HIV	Medical Outcome Study in HIV
PCR	Reacción en cadena de polimerasa
RTV	Ritonavir
TARGA	Tratamiento antirretroviral de gran actividad
TDF	Tenofovir
TDF+3TC	Truvada
VIH-1	Virus de Inmunodeficiencia Adquirida

ÍNDICE

1 RESUMEN 2

2 GLOSARIO ABREVIATURAS 7

3 ÍNDICE 8

4 INFORMACIÓN GENERAL 10

5 JUSTIFICACIÓN Y OBJETIVOS 11

5.1 JUSTIFICACIÓN 11
5.2 OBJETIVOS DEL ESTUDIO: 13
5.2.1 OBJETIVO PRIMARIO: 13
5.2.2 OBJETIVOS SECUNDARIOS: 13

6 TIPO DE ENSAYO CLÍNICO Y DISEÑO DEL MISMO 14

6.1 DISEÑO 14
6.2 REGISTRO DE LOS PACIENTES 14
6.3 ALEATORIZACIÓN DE LOS PACIENTES 14

7 SELECCIÓN DE LOS SUJETOS 15

7.1 NÚMERO DE SUJETOS PREVISTOS 15
7.2 CRITERIOS DE SELECCIÓN 15
7.3 INCUMPLIMIENTO DE CRITERIOS DE SELECCION 17
7.4 SUSPENSIÓN DEL TRATAMIENTO. RETIRADA DEL ESTUDIO. 17
7.5 DURACIÓN ESTIMADA DEL PERÍODO DE RECLUTAMIENTO 17

8 DESCRIPCIÓN DEL TRATAMIENTO 18

8.1 ESQUEMA DE ADMINISTRACIÓN DE LOS FÁRMACOS 18
8.2 MEDICACIÓN ANTERIOR Y CONCOMITANTE 19
8.3 DESCRIPCIÓN DE LOS FÁRMACOS DEL ESTUDIO 20
8.4 SUMINISTRO DE MEDICACIÓN DEL ESTUDIO 21

9 DESARROLLO DEL ENSAYO Y EVALUACIÓN DE LA EFICACIA 22

9.1 VARIABLES EN EL ESTUDIO E INSTRUMENTOS DE MEDIDA 22
9.1.1 VARIABLE PRINCIPAL DE VALORACIÓN: 22
9.1.2 VARIABLES SECUNDARIAS DE VALORACIÓN: 22
9.2 DESARROLLO DEL ENSAYO 23
9.2.1 CALENDARIO Y PROCEDIMIENTOS DEL ESTUDIO. LOS PROCEDIMIENTOS SE PRESENTAN EN LA SIGUIENTE TABLA Y SE DETALLA A CONTINUACIÓN: 23

9.2.2 INCLUSIÓN DEL SUJETO Y ASIGNACIÓN DEL TRATAMIENTO 24
9.2.3 EVALUACIONES DEL TRATAMIENTO 24
9.2.4 EVALUACIONES TRAS EL TRATAMIENTO 27
9.2.5 INTERRUPCIÓN PREMATURA DEL ESTUDIO 28
CRITERIOS PARA LA SUSPENSIÓN O CAMBIO DEL TRATAMIENTO 28

10 ACONTECIMIENTOS ADVERSOS Y TRATAMIENTO DE LA TOXICIDAD 29

10.1 ACONTECIMIENTOS ADVERSOS 29
10.2 TOXICIDADES 33

11 ASPECTOS ÉTICOS 34

11.1 CONSIDERACIONES GENERALES 34
11.2 OBTENCION DE CONSENTIMIENTO INFORMADO 34

12 CONSIDERACIONES PRÁCTICAS 34

12.1 RESPONSABILIDADES DE LOS PARTICIPANTES EN EL ESTUDIO 34
12.2 MANEJO Y ARCHIVO DE DATOS 35
12.3 CONDICIONES DE PUBLICACIÓN 35
12.4 PROCEDIMIENTO PARA LAS MODIFICACIONES DEL PROTOCOLO 36
12.5 COMITÉ ÉTICO DE INVESTIGACIÓN CLÍNICA (CEIC) 36

13 ANALISIS ESTADÍSTICO 36

13.1 TAMAÑO DE LA MUESTRA Y JUSTIFICACIÓN 37
13.2 POBLACIÓN DEL ANÁLISIS 38
13.3 CRITERIO DE VALORACIÓN PRIMARIO 38
13.4 CRITERIO DE VALORACIÓN SECUNDARIO 38
13.5 MÉTODOS ESTADÍSTICOS. 39

14 BIBLIOGRAFÍA 40

15 APENDICES AL PROTOCOLO 45

15.1 APENDICE 1 46
15.2 APÉNDICE 2 47
15.3 APÉNDICE 3 54
15.4 APÉNDICE 4 69
15.5 APÉNDICE 5 70

INFORMACIÓN GENERAL

A. Identificación del ensayo

1.- Código de protocolo:	SAI-CDV-2009-01
2.- Código EudraCT:	2009-014430-25
3.- Título:	Ensayo clínico abierto, aleatorizado para comparar la calidad de vida de los pacientes VIH+ que inician monoterapia con comprimidos de LPV/r vs triple terapia que contengan un IP potenciado.

B. Tipo de ensayo clínico:	Estudio aleatorizado, fase IV, abierto, prospectivo, nacional y multicéntrico.
C. Descripción de los productos en estudio:	Kaletra®: (Lopinavir/ritonavir)<br>Comprimidos recubiertos con película que contienen 200 mg de lopinavir co-formulado con 50 mg de ritonavir.<br>Vía de administración: Oral.<br>Grupo Terapéutico: Inhibidor de la proteasa (J05AE06)
D. Datos relativos al promotor:	Sociedad Andaluza de Enfermedades Infecciosas. SAEI<br>C/ Reposo 6, bajo 6 (41002).Sevilla<br>Teléfono: 954389553<br>e-mail:secretariatecnica@saei.e.telefonica.net
E. Identificación del responsable del proceso de monitorización:	Effice spi S.L.<br>c/ Capitán Haya, 51 4º5<br>28020 Madrid

JUSTIFICACIÓN Y OBJETIVOS

JUSTIFICACIÓN

El tratamiento antirretroviral actual en adultos infectados por el VIH se basa en la combinación de dos Inhibidores de la transcriptasa inversa análogos de nucleósidos (ITIAN) combinado con un Inhibidor de la transcriptasa inversa no análogo de nucleósidos (ITINN) ó con un Inhibidor de la proteasa (IP) potenciados con una baja dosis de ritonavir (RTV) **[1].**

A pesar del gran éxito del tratamiento antirretroviral de gran actividad (TARGA), los regímenes de triple terapia se asocian a toxicidades a largo plazo, altos costes y una complejidad de administración que podría tener consecuencias directas en el paciente infectado por el VIH por una disminución de la adherencia al tratamiento, por la insatisfacción con el mismo, por provocar un impacto negativo en la Calidad de Vida (CV), y sobre todo, porque podría afectar a la eficacia del tratamiento. **[1-5]**

La triple terapia basada en análogos y un IP se ha asociado a una serie de efectos adversos metabólicos importantes tales como la redistribución de las grasas corporales (lipoatrofial facial y en miembros, obesidad central) e hiperlipidemia (hipercolesterolemia e hipertrigliceridemia.) Aunque inicialmente la gran mayoría de los efectos adversos metabólicos fueron atribuidos al uso de IP, con el pasar de los años ha ganado peso la hipótesis de que efectos adversos como la lipoatrofia están más relacionados con terapias antirretrovirales que contengan ITIAN que con los IP. En el estudio Prometheus **[6]** la incidencia de lipoatrofia fue mayor en los pacientes que recibieron tratamiento con nucleósidos que en los que sólo fueron tratados con doble IP (ritonavir y saquinavir combinados.)

Otro efecto adverso importante del TARGA es una forma de acidosis láctica causante de una elevada mortalidad **[7].** Se acepta actualmente que la acidosis láctica grave en pacientes que reciben dos nucléosidos y un IP es debido a la toxicidad mitocondrial inducida por nucléosidos, en particular el d4T.

También el uso de un IP potenciado con RTV puede complicar el régimen de tratamiento para algunos pacientes. Este régimen basado en un IP requiere, no solo la administración adicional de cápsulas de RTV, sino también especiales condiciones de almacenaje, debido a la recomendación de mantener las cápsulas refrigeradas **[8].** Kaletra® es una coformulación de LPV/r con una gran potencia antiviral y que ha demostrado una eficacia y seguridad a largo plazo en combinación con otros agentes antirretrovirales **[9].**

LPV/r se caracteriza por una barrera farmacológica y genética muy elevada. Las concentraciones plasmáticas valle se encuentran más de 70 veces por encima de la EC_{50} del VIH salvaje. La aparición de resistencias a LPV/r requiere la acumulación de mutaciones

en el gen de la proteasa. A partir de 5 mutaciones empieza a aumentar de forma clínicamente significativa la EC_{50} del VIH salvaje **[10-11]** En el estudio 863 ninguno de los pacientes tratados con LPV/r en los que la viremia rebotó a más de 400 copias/mL desarrolló mutaciones en el gen de la proteasa **[12]**

Además, la nueva formulación de LPV/r en comprimidos reduce la sobrecarga de pastillas y también elimina el requisito de refrigeración. Tampoco requiere la administración de la medicación acompañada de una ingesta alimenticia, **[9]** por tanto, sumando estos beneficios, promueve una simplificación del tratamiento antirretroviral y potencialmente mejora la adherencia.

En los últimos años se han venido publicando datos del empleo de LPV/r en monoterapia: de inicio **[13],** de mantenimiento tras inicio **[14**] y de mantenimiento tras tratamiento TARGA con indetectabilidad sostenida de al menos 6 meses **[15-17].**

En concreto, la estrategia que hasta el momento se ha presentado como más segura para el empleo de LPV/r en monoterapia es la tercera, habiéndose publicado datos del estudio OK04 (aleatorizado, comparativo de 200 pacientes) a 48 semanas **[15]** y recientemente a 96s **[18]**

En otro estudio abierto (MONARK) en el que los pacientes naïve fueron asignados aleatoriamente a recibir una monoterapia basada en LPV/r® o triple terapia antirretroviral estándar, utilizando LPV/r (Kaletra®)+ AZT/3TC (Combivir®) la eficacia virológica fue similar en ambos grupos a las 48 semanas de tratamiento, aún en aquellos pacientes en el grupo de monoterapia que tuvieron episodios de niveles más bajos de viremia.**[5]** Además, la media del total de los síntomas reportados y el número de síntomas que provocaron malestar fueron significativamente mayores en el grupo de triple terapia comparado con el grupo en monoterapia (riesgo relativo 1.3 y 1.4; P= 0.001 y P= 0.0003 respectivamente) y la proporción de pacientes con una percepción positiva de su calidad de vida general aumentó, en el grupo de monoterapia, de 32% al momento basal hasta el 68% a las 48 semanas de estudio (P<0.0001), y en el grupo de triple terapia desde 46% al 59% (P= 0.38)**[5]**

Finalmente, en un estudio a 48 semanas, aleatorizado, abierto, en pacientes VIH+ con una replicación viral inhibida, que cambiaban de triple terapia antirretroviral basada en LPV/r a monoterapia basada en LPV/r, la proporción de pacientes que permaneció con una carga viral indetectable en el grupo en monoterapia no fue estadísticamente diferente de los pacientes del grupo que mantenían la triple terapia (81% Vs. 95%, P=0.34)**[5]**

<u>Fundamento del estudio actual:</u>

A lo largo de estos últimos años la estrategia de simplificación de LPV/r en monoterapia ha demostrado ser no inferior en eficacia virológica e inmunológica que la triple terapia.

En España desde enero de 2008 se ha incluido en las guías de tratamiento, el uso de la simplificación a monoterapia con LPV/r como opción terapéutica para aquellos pacientes, sin historia de fracaso previo a IP, con una carga viral indetectable al menos 6 meses y signos y/o síntomas de toxicidad por los ITIAN **[19].**

El objetivo de éste estudio a 24 semanas, aleatorizado, abierto es el de comparar la Calidad de Vida de aquellos pacientes que inician monoterapia con comprimidos de LPV/r coformulado versus pacientes con TARGA basada en cualquier IP potenciado.

OBJETIVOS DEL ESTUDIO:

1.1.1 Objetivo primario:

- comparar la calidad de vida de pacientes que inicien simplificación a monoterapia con comprimidos de lopinavir/ritonavir (LPV/r) vs aquellos que continúan en triple terapia que contengan cualquier Inhibidor de la Proteasa potenciado (IP).

1.1.2 Objetivos secundarios:

- Evaluación de la satisfacción del paciente con el tratamiento de los pacientes VIH+ que inician simplificación a monoterapia con comprimidos de LPV/r vs triple terapia que contengan cualquier IP potenciado.
- Evaluación de la adherencia al tratamiento de los pacientes VIH+ que inician simplificación a monoterapia con comprimidos de LPV/r vs triple terapia que contengan cualquier IP potenciado.
- Evaluación de la tolerabilidad y seguridad de las pautas en los pacientes VIH+ que inician simplificación a monoterapia con comprimidos de LPV/r vs triple terapia que contengan cualquier IP potenciado.
- Evaluación de la eficacia virológica de la simplificación a monoterapia basada en comprimidos de LPV/r vs triple terapia que contengan cualquier IP potenciado.
- Evaluación de los cambios en la respuesta inmune en los pacientes VIH+ que inician la simplificación a monoterapia con comprimidos de LPV/r vs triple terapia que contengan cualquier IP potenciado.

TIPO DE ENSAYO CLÍNICO Y DISEÑO DEL MISMO

DISEÑO

Estudio aleatorizado, fase IV, abierto, prospectivo, nacional y multicéntrico en pacientes VIH+ en triple terapia antirretroviral que contengan cualquier Inhibidor de la Proteasa potenciado (IP) versus pacientes que inicien simplificación a monoterapia con comprimidos de LPV/r.

REGISTRO DE LOS PACIENTES

Cuando se considere que un paciente es susceptible de participar en el estudio, se le ofrecerá participar en el estudio, para lo cual se le proporcionará la Hoja de Información al Paciente (Apéndice 3) y se le facilitará toda la información adicional que precise.

Una vez obtenido el Consentimiento informado del paciente, se enviará por fax al **Centro de Datos del estudio** el formulario de inclusión del paciente con el fin de obtener la asignación del grupo de tratamiento y nº de selección del paciente. Para más información, diríjanse a la sección 5.3. Aleatorización de los pacientes.

En el formulario de inclusión se detallaran los siguientes datos:

- Número de orden de selección en el centro del paciente.
- Centro investigador
- Investigador

ALEATORIZACIÓN DE LOS PACIENTES

La aleatorización será centralizada y se realizará desde el Centro de Datos del estudio, tras la recepción de los datos.

Antes de que los pacientes inicien el Estudio, serán asignados aleatoriamente según un listado de randomización, dicho listado será creado mediante el generador de números pseudos-aleatorios de Wichmann and Hill (1982) y modificado por McLeod (1985). El tamaño de los bloques será de 4 para intentar balancear las ramas de tratamiento en el total de la muestra y dentro de cada centro. No se ha planeado estratificar por ningún otro factor.

El listado de randomización estará centralizado y oculto a los investigadores, quienes accederán al tratamiento asignado mediante consulta telefónica en el momento de la aleatorización con el Promotor o con quien éste asigne en su nombre. Se enviará, previamente a la llamada telefónica, por fax al Promotor la página del CRD correspondiente,

en la que se confirma que el paciente reúne todos los criterios de inclusión y ninguno de exclusión, a fin de conocer la rama del Estudio que se le asigna y el número de paciente a emplear durante el mismo. Esta información se devolverá escrita al investigador en el mismo formato recibido para su solicitud e incluyendo el número asignado al paciente y la rama del Estudio.
La asignación aleatoria será según la relación 1:2 a uno de las siguientes dos opciones:

Grupo control (A): Los pacientes continuarán la triple terapia antirretroviral basada en cualquier IP potenciado con ritonavir.

Grupo experimental (B): Cambiar la triple terapia antirretroviral basada en cualquier IP potenciado con ritonavir a LPV/r monoterapia (comprimidos co-formulados.)

SELECCIÓN DE LOS SUJETOS

NÚMERO DE SUJETOS PREVISTOS

El número de pacientes a incluir en este estudio será de **390 pacientes** (130 pacientes para el grupo control A y 260 para el grupo experimental B.) La inclusión será competitiva y se espera que haya entre 12 y 13 pacientes por centro.

CRITERIOS DE SELECCIÓN

Antes del comienzo de los procedimientos específicos del protocolo, deberá obtenerse y documentarse el consentimiento informado del paciente por escrito.

Criterios de Inclusión:

- Pacientes infectados por VIH-1 documentada por prueba de anticuerpos frente a VIH-1 positiva y/o PCR positiva confirmadas para ARN del VIH-1
- Pacientes en triple terapia antirretroviral que contenga cualquier IP potenciado.
- Pacientes con una carga viral indetectable definida como < 50 copias/mL en los últimos 6 meses.
- Varones o mujeres de edad ≥ 18 años.
- Para mujeres en edad fértil, prueba de embarazo en orina negativa en la visita de selección.
- Pacientes que hayan otorgado el consentimiento informado por escrito antes de realizar cualquier procedimiento de selección específico del estudio.

Criterios de exclusión:

- Pacientes con constancia documentada, en el genotipado acumulado, de mutaciones de resistencia que confieran pérdida de sensibilidad a LPV/r® ó, en ausencia de genotipado, la existencia de pruebas de haber fracasado a una terapia con Inhibidores de la Proteasa (IP).
- Pacientes con un nadir de células CD4 <100 cel/μL.
- Pacientes que por cualquier motivo no puedan ser tratados con LPV/r.
- Antecedentes de desórdenes psiquiátricos como Síndrome Depresivo, Esquizofrenia o enfermedad Psicótica.
- Antecedentes conocidos de drogadicción o consumo crónico de alcohol que, en opinión del investigador, contraindican su participación en el estudio.
- Mujeres embarazadas o en período de lactancia o mujeres en edad fértil que no utilizan un método anticonceptivo adecuado según el criterio del investigador.
- Infección oportunista activa actual o infección documentada en el plazo de 4 semanas anteriores a la selección.
- Pacientes a los que por toxicidades graves relacionados con cualquier componente de la triple terapia antirretroviral actual se tenga previsto modificar o retirar cualquiera de los componentes de dicha terapia.
- Pacientes en los que los investigadores consideren que se debe cambiar la medicación antirretroviral, por cualquier causa o motivo, en los próximos 6 meses.
- Enfermedad renal con aclaración de creatinina <60 mL/min.
- Uso concomitante de fármacos contraindicados con LPV/r como rifampicina, dihidroergotamina, ergotamina, metilergonovina, cisapride, hipericum perforatum, lovastatina, simvastatina, pimozide, midazolam y triazolam.
- Uso concomitante de fármacos nefrotóxicos o inmunosupresores.
- Paciente en tratamiento actual con corticoesteroides sistémicos, interleucina-2 o quimioterapia.
- Pacientes en tratamiento con otros agentes en investigación.
- Pacientes con hepatitis aguda.
- Cualquier enfermedad que, a juicio del investigador, contraindique la participación del paciente en el estudio.

INCUMPLIMIENTO DE CRITERIOS DE SELECCION

Todos los pacientes deben cumplir todos los criterios de inclusión y ninguno de exclusión. Para evitar la inclusión de pacientes no elegibles, se recomienda consultar cualquier duda con el Promotor.

Si un paciente no cumpliera los criterios de elegibilidad y fuera incluido inadvertidamente en el estudio se deberá comunicar al Promotor del estudio a fin de que éste valore la continuidad del paciente en el estudio, evaluando los riesgos y beneficios para la paciente y garantizando su máxima seguridad.

SUSPENSIÓN DEL TRATAMIENTO. RETIRADA DEL ESTUDIO.

Cualquier paciente será retirado del tratamiento del estudio en estas circunstancias:

- A solicitud del paciente.
- Evento adverso grave o toxicidad inaceptable.
- Embarazo.
- Cuando el investigador considere que para mayor beneficio del paciente es necesario un cambio de tratamiento no contemplado en el protocolo.

La fecha y los motivos de cualquier interrupción prematura se anotarán en el Cuaderno de Recogida de Datos (CRD) y serán tenidas en cuenta en la valoración final.

DURACIÓN ESTIMADA DEL PERÍODO DE RECLUTAMIENTO

El reclutamiento comenzará una vez se hayan obtenido las aprobaciones oportunas. La duración del reclutamiento se prevé en 8 meses. La duración total del estudio, contando con período de reclutamiento y las 24 semanas que dura el tratamiento, se prevé en 12 meses.

DESCRIPCIÓN DEL TRATAMIENTO

ESQUEMA DE ADMINISTRACIÓN DE LOS FÁRMACOS

Los pacientes se aleatorizarán a uno de los siguientes grupos de tratamiento:

Grupo control (A): Los pacientes continuarán la triple terapia antirretroviral basada en cualquier IP potenciado con ritonavir.
La medicación será administrada según indica su propia ficha técnica.

Grupo experimental (B): Cambio a LPV/r en monoterapia.
Solamente, Kaletra® 2 comprimidos, dos veces al día.

CRITERIO DE RETIRADA DEL PACIENTE

Fracaso terapéutico

Aquellos pacientes que cumplan criterios de fracaso terapéutico serán retirados del estudio. Estos criterios se definen a continuación:

1. En pacientes que reciben triple terapia a la que se le aleatorizó en la basal, se considera fracaso terapéutico el fracaso virológico confirmado, es decir, la presencia de una carga viral mayor de 500 copias/ml en 2 ocasiones separadas 15 días.
2. Los pacientes asignados al mantenimiento sólo con LPV/r que alcancen el criterio de fracaso virológico se considerarán fracaso terapéutico si existe presencia de mutaciones en el gen de la proteasa, por lo que el paciente no puede ser reinducido con nucleósidos y abandonaría el estudio al necesitar una terapia no basada en LPV/r.
3. Sin embargo, los pacientes asignados al mantenimiento sólo con LPV/r podrán ser reinducidos con los mismos nucleósidos que se retiraron si no existe constancia de resistencia a LPV/r. Por tanto, específicamente NO será considerada como fracaso terapéutico ni supondrá discontinuación del estudio la siguiente situación (a+b+c):
    a) Un paciente en la rama de mantenimiento con sólo LPV/r presente una carga viral mayor de 500 copias/ml en 2 ocasiones separadas 15 días.
    b) El test de resistencia genotípicas no muestre resistencias a LPV/r.
    c) El paciente sea reinducido con los mismos nucleósidos que recibía antes de entrar en el estudio y finaliza el período de seguimiento con CV indetectable.

Dada esta situación, y una vez reinducidos, sí se considerarán como fracaso terapéutico cuando se cumpla que:

- a las 4 semanas tras la reinducción la carga viral no haya descendido al menos una unidad logarítmica decimal
- y/o no vuelva a quedarse indetectable (<50 copias/ml) a las 16 semanas de la reinducción
- o si una vez en terapia triple vuelven a tener fracaso virológico.

Aquellos pacientes en los que se obtengan carga viral entre 50-500 copias/ml se procederá según queda explicado anteriormente.

Tras confirmar esta carga viral en 3 ocasiones (2 semanas, 2 semanas y 4 semanas) se procederá de la siguiente manera:

- Pacientes en el grupo de monoterapia y sin resistencias a Lopinavir se reinduce con los mismos nucleósidos y se procede según los párrafos anteriores.
- Pacientes en triple terapia o en el grupo de monoterapia pero con resistencia a Lopinavir: se considera fracaso terapéutico y tras diseñar tratamiento de rescate se programa una visita de seguimiento 4 semanas después de la visita fin de estudio.

En el anexo 1 del protocolo se presenta el manejo de los pacientes con fallo virológico de modo esquemático.

Intolerancia

Efecto adverso grave según se define en el apartado 7.7 (Criterios de modificación de pauta por toxicidad).

MEDICACIÓN ANTERIOR Y CONCOMITANTE

Los pacientes continuarán con sus medicaciones concomitantes según se receten. Toda la medicación concomitante se deberá registrar en el Cuaderno de Recogida de Datos (CRD). Además, se deberá registrar cualquier procedimiento diagnóstico, terapéutico o quirúrgico que se realice durante el período de estudio, incluyendo la fecha, indicación, descripción del(los) procedimiento(s) y cualquier hallazgo clínico.

A efectos de garantizar la seguridad del paciente, se excluirán las siguientes medicaciones no recomendadas concomitantemente con el de Kaletra®:

Kaletra®. Contiene lopinavir/ritonavir, 2 inhibidores de la isoforma CYP3A del P450 por lo que no debería administrarse conjuntamente con medicamentos cuyo aclaramiento dependa en gran medida del CYP3A y para los que un aumento de las concentraciones plasmáticas esté asociado con efectos graves y/o que supongan una amenaza para la vida. Estos medicamentos incluyen astemizol, terfenadina, midazolam oral, triazolam, cisaprida, pimozida, amiodarona, alcaloides ergotamínicos (ej: ergotamina, dihidroergotamina, ergonovina y metilergonovina) y vardenafilo.

Los medicamentos a base de plantas que contengan hierba de San Juan (Hypericum perforatum) no deben utilizarse mientras se esté tomando lopinavir y ritonavir, debido al riesgo de que reduzcan las concentraciones plasmáticas y los efectos clínicos de lopinavir y ritonavir. La rifampicina no debe utilizarse en combinación con Kaletra®, debido a que puede reducir notablemente las concentraciones de lopinavir y, por consiguiente, disminuir significativamente su efecto terapéutico.

Si los pacientes necesitan comenzar el tratamiento con cualquiera de las medicaciones excluidas, debe consultarse con el Coordinador del estudio antes del inicio de la misma.

DESCRIPCIÓN DE LOS FÁRMACOS DEL ESTUDIO

Descripción y Manipulación de Kaletra®:

Formulación:

Cada comprimido de Kaletra® recubierto con película contiene 200 mg de lopinavir y 50 mg de ritonavir como potenciador. Los comprimidos de Kaletra® 200/50 mg son comprimidos recubiertos amarillos, marcados en relieve con el logo de Abbott y “KA”.

Excipientes: cada comprimido contiene copovidona, laureato de sorbitán, sílice coloidal anhidra y estearil fumarato de sodio. Véase el etiquetado del producto para más información.

Acondicionamiento y Etiquetado:

Blíster de lámina de PVC/ fluoropolímero. Cada cartón contiene 5 blisters de aluminio con 8 comprimidos recubiertos con película cada uno (40 comprimidos.) Cada embalaje contiene 3 cajas (120 comprimidos.)

Almacenamiento y Manipulación:

Este producto no requiere condiciones especiales de conservación.

SUMINISTRO DE MEDICACIÓN DEL ESTUDIO

Durante la realización del estudio se proveerá del fármaco Kaletra (sin coste para el centro hospitalario) a todo paciente que, por motivo de participar en el mismo, precise iniciar el tratamiento con esta especialidad farmacéutica. La medicación será identificada como "medicación de ensayo" y, por tanto, el etiquetado no será el comercial.

En el anexo 2 del protocolo se explica el proceso de solicitud de medicación y, el de re-etiquetado de la medicación para uso exclusivo de ensayo clínico, de modo esquemático.

DESARROLLO DEL ENSAYO Y EVALUACIÓN DE LA EFICACIA

VARIABLES EN EL ESTUDIO E INSTRUMENTOS DE MEDIDA

1.1.3 Variable Principal de valoración:

- Cambios a las 24 semanas (desde la visita basal) en la calida de vida en función de los parámetros del Medical Outcomes Study HIV Health Survey (MOS-HIV) y la escala visual analógica EuroQol 5D (EQ-5D.)**[20]**

1.1.4 Variables Secundarias de valoración:

- Evaluación de la satisfacción del paciente con el tratamiento de los pacientes VIH+ que inician monoterapia con comprimidos de LPV/r vs triple terapia que contengan cualquier IP utilizando el cuestionario CESTA (Cuestionario Español de Satisfacción con el tratamiento antirretroviral)**[21]** desde la visita basal a la semana 24 de estudio.
- Evaluación de la adherencia al tratamiento de los pacientes VIH+ que inician monoterapia con comprimidos de LPV/r vs triple terapia que contengan cualquier IP a través de los cuestionarios GEEMA **[22]** y una escala visual analógica (EVA)**[23]**.
- Evaluación de la tolerabilidad y seguridad de las pautas en los pacientes VIH+ que inician monoterapia con comprimidos de LPV/r vs triple terapia que contengan cualquier IP.
- Porcentaje de pacientes con una carga viral ARN-VIH <50 copias/mL a las 24 semanas, en los pacientes VIH+ que inician monoterapia con comprimidos de LPV/r vs triple terapia que contengan cualquier IP. Se considerará fallo virológico a una carga viral >500 copias/mL de ARN Viral (HIV1) confirmada a la semana 24 de estudio.
- Cambios en la respuesta inmune en los pacientes VIH+ que inician monoterapia con comprimidos de LPV/r vs triple terapia que contengan cualquier IP medido a través del cambio del recuento de células CD4+ desde la visita basal a la semana 24 de estudio

1.1.5 Calendario y procedimientos del estudio. Los procedimientos se presentan en la siguiente tabla y se detalla a continuación:

	Visita Selección	Visita inicial	Visita semana 4	Visita semana 12	Visita semana 24	Visita interrupción prematura
Aleatorización		X (24 hs antes)				
Consentimiento Informado	X	X				
Historia Clínica-TARV previo	X	X				
Examen Físico completo (1)	X	X		X	X	X
Test de embarazo (2)	X	X				
ARN del VIH-1 en plasma	X	X	X*	X	X	X
Recuento CD4	X	X	X*	X	X	X
Hepatología**	X	X		X	X	X
Bioquímica (2)**	X	X		X	X	X
**Cuestionario MOS-HIV**		X			X	X
**Cuestionario EQ-5D**		X			X	X
Cuestionario CESTA		X	X*	X	X	X
Cuestionario GEEMA		X	X*	X	X	X
EVA adherencia		X	X*	X	X	x
Medicación Concomitante		X	X*	X	X	X
Acontecimientos Adversos		X	X*	X	X	X

(1) incluye signos vitales y medida de peso. Al tratarse de pacientes adultos, la altura será evaluada en la visita de inicio.

*Solamente para los pacientes del brazo experimental.

** Recoger los datos relevantes en relación a los acontecimientos adversos.

(2) Test de embarazo en orina para mujeres en edad fértil.

Cualquier desviación de los procedimientos del protocolo debe notificarse al Promotor y recogerse en los Cuadernos de Recogida de Datos (CRD).

Todas las muestras de laboratorio se enviarán a laboratorios locales para su análisis. Debe utilizarse el mismo laboratorio local durante la totalidad de la participación de un paciente en el ensayo.

1.1.6 Inclusión del sujeto y asignación del tratamiento

Es responsabilidad del investigador garantizar que cada sujeto es elegible para el estudio antes de su inclusión. Una vez que se ha asignado a un sujeto un número de sujeto, no se volverá a asignar a ningún otro sujeto.

1.1.7 Evaluaciones del tratamiento

Medida de Calidad de Vida:

El manejo de los cuestionarios vendrá específicamente detallado en el Anexo 3. Como carácter general, se deberá presentar cada cuestionario al paciente basándose en las siguientes premisas:

- En los cuestionarios siguientes se interroga al paciente sobre numerosos aspectos de su estado de salud y asistencia sanitaria. Deben facilitarse al paciente antes de la exploración física, preferiblemente en un ambiente tranquilo (p. ej., en la sala de exploración o en otro consultorio).

- Es importante familiarizarse con el contenido y el formato de los cuestionarios antes de entregárselos a los participantes en el estudio. En la primera visita, indique al paciente lo siguiente:

“Le rogamos que responda a algunas preguntas sobre cómo se siente y lo que es capaz de hacer. Sus respuestas nos ayudarán a comprender los efectos de la medicación que está tomando. Le agradeceríamos que rellenara estos cuestionarios.”

- A continuación, debe explicar brevemente el formato de las preguntas y cómo cumplimentarlas. El paciente deberá cumplimentar los cuestionarios antes de que se realicen la historia clínica y la exploración física y se determinen las constantes vitales.

- Los cuestionarios son muy breves y no deben tardarse más de 15-30 minutos en cumplimentarlos. Antes de entregárselos al paciente, rellene el encabezado y SEPARE LA PÁGINA destinada al investigador que viene correctamente indicada.

- Recoja los cuestionarios cumplimentados antes de la exploración clínica. Antes de continuar, revíselos en busca de posibles omisiones. Si el participante ha dejado alguna pregunta sin contestar, indíqueselo y haga que subsane las omisiones.

Otros procedimientos a realizar en el estudio:

Independientemente de las medidas de Calidad de Vida (apartado 8.2 que se realizarán con los cuestionarios correspondientes, seguidamente se describen, además, los procedimientos adicionales que se permitirán cubrir los objetivos secundarios del estudio.

Visita de Selección:

Se determinará la elegibilidad de cada sujeto en un plazo máximo de 3 semanas previas a la aleatorización en el estudio. Se deberá realizar en esta visita y se deberá documentar lo siguiente:

- Obtención del Consentimiento Informado por escrito.
- Historia Clínica que incluya historial de acontecimientos relacionados con la enfermedad y fármacos utilizados en los 30 días anteriores.
- Exploración física (signos vitales, peso corporal y altura)
- Prueba de embarazo en orina (mujeres en edad fértil).
- Hemograma completo (Recuento de Glóbulos rojos, Glóbulos blancos, Fórmula leucocitaria y Recuento de plaquetas)
- Perfil bioquímico en ayunas (Fosfatasa alcalina, ALT, AST, GGT, Bilirrubina total, Creatinina, Glucosa, Potasio, Sodio, Acido Urico y Perfil Lipídico (Colesterol total, HDLc, LDLc y Triglicéridos)
- Recuento de células CD4
- ARN del VIH-1 en plasma

Visita Inicial:

Si los datos de laboratorio para confirmar los criterios de selección se han obtenido en un plazo de dos meses antes de la inclusión en el estudio y se dispone de los mismos, la visita de selección puede coincidir con la visita inicial.

Los fármacos del estudio deben comenzar a tomarse en el plazo de 24 horas tras la visita inicial.

En la visita inicial deben estar disponibles las siguientes evaluaciones:

- Exploración física (signos vitales, peso corporal y altura)
- Cuestionarios para la evaluación de la calidad de vida (MOS-HIV y EQ-5D)
- Test de embarazo en orina negativo para mujeres en edad fértil.
- Hemograma completo (Recuento de Glóbulos rojos, Glóbulos blancos, Fórmula leucocitaria y Recuento de plaquetas)
- Perfil bioquímico en ayunas (Fosfatasa alcalina, ALT, AST, GGT, Bilirrubina total, Creatinina, Glucosa, Potasio, Sodio, Acido Úrico, Perfil Lipídico (Colesterol total, HDLc, LDLc y Triglicéridos).
- Recuento de células CD4
- ARN del VIH-1 en plasma: Se realizará en tiempo real en el centro utilizando la metodología virológica habitual en cada centro. Para cada sujeto se deberá utilizar la misma metodología a lo largo de todo el estudio.
- Cuestionario CESTA para evaluar la satisfacción con el tratamiento antirretroviral.
- Cuestionario GEEMA para evaluar la adherencia al tratamiento
- EVA para evaluar la adherencia al tratamiento
- Registro de medicación concomitante
- Acontecimientos adversos

Una vez revisados los procedimientos y evaluaciones de la visita inicial, se aleatorizará al sujeto de manera centralizada en el Centro de Datos del estudio, tras la recepción de los datos.

Los pacientes aleatorizados para el grupo B, volverán para un control del ARN-VIH en la semana 4 de tratamiento (+/- 2 días)
Las siguientes evaluaciones deben completarse en las **visitas de las semanas 12 y 24 o visita de interrupción del estudio**. Las visitas del estudio se deben programar en el plazo de ± 1 semana de la fecha especificada para la semana 12 y semana 24, calculando las fechas en función de la visita inicial:

- Exploración física (signos vitales, peso corporal)
- Hemograma completo (Recuento de Glóbulos rojos, Glóbulos blancos, Fórmula leucocitaria y Recuento de plaquetas.)

- Perfil bioquímico en ayunas (Fosfatasa alcalina, ALT, AST, GGT, Bilirrubina total, Creatinina, Glucosa, Potasio, Sodio, Acido Úrico, Perfil Lipídico (Colesterol total, HDLc, LDLc y Triglicéridos)
- Recuento de células CD4
- ARN del VIH-1 en plasma: En caso de que en algún momento el paciente muestre una carga viral detectable (> 50 copias/ml) el paciente se volverá a someter a dicha prueba en el plazo de 2 semanas para su confirmación. En el anexo "1" se pueden ver las sugerencias de manejo de estos casos.
- Medicación concomitante
- Acontecimientos adversos

Las siguientes evaluaciones deben completarse al final de las **visitas de las semanas 12, 24 o en la visita de interrupción prematura del estudio**.

- Cuestionario para la valoración de la adherencia al tratamiento (GEEMA y EVA)
- Cuestionario para la valoración de la satisfacción con el tratamiento (CESTA)
- Medicación concomitante
- Acontecimientos adversos

Las siguientes evaluaciones deben completarse al final de las **visitas de visita basal y las semanas 24 o en la visita de interrupción prematura del estudio**.

- Cuestionario para la valoración de la calidad de vida (MOS-HIV y EQ-5D)
- Cuestionario para la valoración de la adherencia al tratamiento (GEEMA y EVA)
- Cuestionario para la valoración de la satisfacción con el tratamiento (CESTA)
- Medicación concomitante
- Acontecimientos adversos

1.1.8 Evaluaciones tras el tratamiento

Sólo en caso de que se haya reportado cualquier acontecimiento adverso que pueda estar en curso de la última visita ó, si se hubiera presentado una carga viral >50 copias/mL en aquella, Se realizará un seguimiento telefónico, tras 30 días de la última visita del estudio, para hacer un seguimiento de seguridad,

1.1.9 Interrupción prematura del estudio

En caso de retirada prematura, el sujeto debe acudir al centro lo antes posible (preferiblemente en el plazo de 72 horas después de dejar de tomar la medicación del estudio) para una visita de Interrupción prematura del estudio. En dicha visita deben completarse las siguientes evaluaciones:

- Exploración física (signos vitales, peso corporal)
- Hemograma completo (Recuento de Glóbulos rojos, Glóbulos blancos, Fórmula leucocitaria y Recuento de plaquetas)
- Perfil bioquímico en ayunas (Fosfatasa alcalina, ALT, AST, GGT, Bilirrubina total, Creatinina, Glucosa, Potasio, Sodio, Acido Úrico, Perfil Lipídico (Colesterol total, HDLc, LDLc y Triglicéridos)
- Recuento de células CD4
- ARN del VIH-1 en plasma: En caso de que en algún momento el paciente muestre una carga viral detectable (> 50 copias/ml) el paciente se volverá a someter a dicha prueba en el plazo de 2 semanas para su confirmación.
- Cuestionario para la valoración de la calidad de vida (MOS-HIV y EQ-5D)
- Cuestionario para la valoración de la adherencia al tratamiento (GEEMA y EVA)
- Cuestionario para la valoración de la satisfacción con el tratamiento (CESTA)
- Medicaciones Concomitantes
- Acontecimientos adversos

Criterios para la suspensión o cambio del tratamiento

La medicación del estudio debe interrumpirse en los siguientes casos:

- Enfermedad subyacente que a criterio del investigador pueda afectar a las evaluaciones de estado clínico de manera significativa.
- Toxicidad inaceptable (toxicidad que compromete la capacidad de continuar con los procedimientos específicos del Protocolo)
- Solicitud del sujeto.
- Embarazo
- Fracaso terapéutico definido por 2 determinaciones de CV> 500 copias/ml separadas por al menos 2 semanas en el grupo experimental y/o genotipado con mutaciones a IP que impida continuar el tratamiento de monoterapia con LPV/r.

ACONTECIMIENTOS ADVERSOS Y TRATAMIENTO DE LA TOXICIDAD

ACONTECIMIENTOS ADVERSOS

Definición de acontecimiento adverso y categorías de acontecimientos adversos graves

El investigador vigilará de forma sistemática durante el estudio a todos los sujetos para detectar AAG.

Se define como AA toda incidencia médica no deseada que aparece en un paciente durante el tratamiento pero que no tiene por qué tener necesariamente una relación causal con su tratamiento. Un AA puede ser, por tanto, cualquier signo desfavorable y no deseado (como un hallazgo anormal de laboratorio), un síntoma o enfermedad asociados temporalmente al uso de un medicamento, se considere o no que el acontecimiento está relacionado causalmente con el uso del producto.

Este acontecimiento puede producirse por el uso del fármaco tal como se estipula en el protocolo o por sobredosis accidental o intencionada, abuso o suspensión del fármaco. Todo empeoramiento de una afección o enfermedad preexistente se considera un AA.

Todos los acontecimientos adversos se anotaran el Cuaderno de Recogida de Datos (CRD.)

Acontecimiento adverso grave (AAG): definición y procedimiento de notificación

Un acontecimiento adverso grave (AAG) es aquel que causa la muerte del paciente, es amenazante para la vida, requiere o prolonga la hospitalización del paciente, da lugar a incapacidad o discapacidad persistente o significativa, se trata de una anomalía congénita o un defecto de nacimiento o un acontecimiento clínicamente importante.

Los acontecimientos clínicamente importantes son aquellos que pueden no ser inmediatamente amenazantes para la vida, pero claramente tienen una significación clínica relevante. Pueden poner en peligro al sujeto o requerir una intervención para evitar cualquier resultado grave. La sobredosificación o el abuso de un fármaco se considerarán, normalmente, como graves.

**Fallecimiento**	Acontecimiento que causa la muerte de un paciente.
**Potencialmente mortal**	Acontecimiento que, en opinión del investigador, podría haber ocasionado la muerte inmediata del paciente de no haberse producido la intervención médica. No se incluyen los acontecimientos que pudieran haber sido mortales de haber sido más intensos.
**Hospitalización**	Acontecimiento que exige el ingreso hospitalario, independientemente del tiempo de hospitalización. No incluye las visitas al servicio de urgencias ni la estancia en un centro ambulatorio.
**Prolongación de la hospitalización**	Acontecimiento que tiene lugar mientras el sujeto del estudio está hospitalizado y prolonga su estancia en el hospital.
**Anomalía congénita**	Anomalía detectada en el momento del parto o después, o cualquier anomalía que produzca la pérdida del feto.
**Discapacidad o incapacidad persistente o importante**	Acontecimiento que ocasiona una alteración que dificulta de forma importante las actividades cotidianas de un paciente. La discapacidad no incluye experiencias que tengan relativamente poca importancia médica, como cefaleas, vómitos, diarrea, gripe o traumatismo accidental (p. ej., un esguince de tobillo).
**Acontecimiento de importancia médica que exija una intervención médica o quirúrgica para evitar un desenlace grave**	Acontecimiento de importancia médica que no cause la muerte, ponga en peligro la vida de manera inmediata ni exija la hospitalización, pero que, a criterio médico, suponga un riesgo para el paciente y exija una intervención médica o quirúrgica para evitar uno de los desenlaces mencionados anteriormente (fallecimiento del paciente, potencialmente mortal, hospitalización o prolongación de la hospitalización, anomalía congénita, o discapacidad o incapacidad persistente o importante). Ejemplos de estos acontecimientos podrían ser el tratamiento intensivo de un broncospasmo alérgico en un servicio de urgencias o en el domicilio del paciente, las discrasias sanguíneas o las convulsiones que no motiven el ingreso hospitalario del paciente, o el desarrollo de dependencia o abuso del fármaco.

Intensidad

El médico utilizará las definiciones siguientes para calificar la intensidad de cada AA de especial interés registrado como criterio de valoración en el estudio y en todos los AAG.

Leve	El acontecimiento adverso es pasajero y el paciente lo tolera fácilmente.
Moderado	El acontecimiento adverso causa molestias al paciente e interrumpe sus actividades habituales.
Intenso	El acontecimiento adverso dificulta considerablemente las actividades habituales del paciente y puede ser incapacitante o potencialmente mortal.

Relación con el fármaco

El médico utilizará las siguientes definiciones en cada AA para establecer la relación de causalidad:

Probablemente relacionado	El acontecimiento adverso guarda una clara relación temporal con el fármaco o reaparece al reanudar la exposición, y es poco probable o significativamente menos probable otra causa.
Posiblemente relacionado	El acontecimiento adverso tiene una marcada relación temporal con el fármaco y es igualmente probable o menos probable que exista otra causa en comparación con la posible relación con el fármaco del estudio.
Probablemente no relacionado	El acontecimiento adverso guarda una relación temporal mínima o nula con el fármaco o existe otra causa posible más probable o ambas cosas.
No relacionado	El acontecimiento adverso se debe a una enfermedad subyacente o simultánea o al efecto de otro medicamento y no está relacionado con el fármaco (por ejemplo: no tiene relación temporal con el fármaco o hay otra causa Muchísimo más probable).

Período de recopilación de acontecimientos adversos

Se notificarán los AAG desde la firma del consentimiento informado y hasta los 30 días posteriores a la última toma de medicación según el procedimiento descrito a continuación. Para cualquier AAG tardío (que se manifieste después de este período de 30 días) que esté posible o probablemente relacionado con la medicación del estudio, se debe seguir el mismo procedimiento para su comunicación.

Notificación de acontecimientos adversos graves

Si se produce un AAG, esté o no relacionado con el fármaco en investigación, El investigador comunicará al Promotor por teléfono o fax, todos los acontecimientos adversos graves, independientemente de su imputabilidad, ocurridos durante el estudio, en las 24 h. siguientes a tener conocimiento de los mismos o en el siguiente día laborable. En el caso de AAG con resultado de muerte o amenazante para la vida, el investigador deberá notificarlo inmediatamente al Promotor:

Nº de Fax: ***915791580***

Nº de teléfono: ***912948910***

Todas las comunicaciones de AAG por vía telefónica deberán ir seguidas del correspondiente informe de notificación por escrito, en las 48 horas siguientes. Para las notificaciones por fax el investigador recogerá la información referente al AAG en el impreso correspondiente.

La información <u>mínima inicial</u> para la notificación de un acontecimiento adverso debe incluir lo siguiente:

- descripción del acontecimiento adverso y fecha de comienzo del mismo.
- código del paciente, sexo y edad.
- Información sobre el tratamiento recibido.
- Nombre y dirección del médico que realiza la notificación.
- Si considera que existe o no relación de causalidad con los fármacos en estudio.

En caso de que haya comunicado un fallecimiento de un sujeto participante, el investigador proporcionará al Promotor y a los CEICs implicados toda la información complementaria que soliciten.

El formulario para la notificación de AAG al Promotor se incluye en el Apéndice 4.

El Promotor se responsabiliza de la notificación de todas las sospechas de Reacciones Adversas Graves e Inesperadas en los plazos de tiempo establecidos por la normativa española vigente, a:

- La Agencia Española del Medicamento y Productos Sanitarios (AEMPS)
- El Comité de Ensayos Clínicos (CEIC) implicado en el ensayo, para las RAGI ocurridas a sujetos participantes pertenecientes a los centros de su área de influencia.
- Los órganos competentes de las Comunidades Autónomas, cuando la sospecha de RAGI haya ocurrido en los centros sanitarios de la Comunidad correspondiente
- A los investigadores principales.

Por otra parte, el Promotor acuerda que notificará al Departamento de Farmacovigilancia de los laboratorios titulares de la Autorización de Comercialización en España de los fármacos en el estudio, cualquier acontecimiento adverso grave o inesperado, de acuerdo con sus procedimientos de notificación.

Para la notificación de las sospechas de RAGI a los CEICs y Autoridades Sanitarias se utilizará en Anexo D proporcionado por las AEMPS en las as Aclaraciones sobre la aplicación de la normativa de ensayos clínicos a partir del 1 de mayo de 2004 (versión núm.4, de 30 de noviembre de 2006)

TOXICIDADES

Todas las toxicidades de laboratorio clínicamente significativas se tratarán según la Clasificación del ACTG de la intensidad de los Acontecimientos Adversos (versión 1.0 Diciembre 2004) (Apéndice 5).

ASPECTOS ÉTICOS

CONSIDERACIONES GENERALES

El presente estudio se llevará a cabo en consonancia con los principios establecidos en la declaración de Helsinki y sus revisiones posteriores (Apéndice 2) y la legislación española vigente sobre ensayos clínicos.

El estudio se conducirá de acuerdo con las normas de Buena Práctica Clínica/ICH.

Existe una póliza de seguro para el estudio contratada con la compañía HDI Hannover Internacional (España) Seguros y Reaseguros, S.A. (póliza número 130/001/006201), que se ajusta a lo establecido en el Real Decreto 223/2004.

OBTENCION DE CONSENTIMIENTO INFORMADO

Antes de realizar ningún procedimiento específico del estudio, se informará al paciente acerca de la naturaleza del tratamiento del estudio y se le facilitará la información pertinente sobre los objetivos buscados, los posibles beneficios y los posibles eventos adversos. Se explicarán al paciente los procedimientos a seguir y los posibles riesgos a los que podría estar expuesto.

El paciente deberá leer y firmar el documento de consentimiento informado que ha sido aprobado. Una vez firmado y fechado por el paciente y el investigador, le facilitará al paciente una copia del consentimiento informado firmado. El paciente podrá retirarse del estudio en cualquier momento, sin que ello afecte a su tratamiento médico futuro.

La Hoja de Información al Paciente y Consentimiento Informado para el estudio se encuentra en el Apéndice 3.

CONSIDERACIONES PRÁCTICAS

RESPONSABILIDADES DE LOS PARTICIPANTES EN EL ESTUDIO

Normas para el paciente

El paciente deberá seguir las indicaciones de los investigadores y comunicar cualquier eventualidad a los mismos.

El paciente será debidamente informado de las prohibiciones o restricciones a las que

deberá atenerse durante la realización del ensayo. El incumplimiento de estas recomendaciones implicará el abandono del estudio.

Los pacientes podrán dar por finalizada su participación en el estudio en cualquier momento.

Normas para el personal investigador

El investigador se compromete a cumplir con las normas establecidas en la legislación vigente en materia de ensayos clínicos: Ley del Medicamento 29/2006 (BOE nº 178, 27.07.06) y Real Decreto 223/2004 de Ensayos Clínicos.

MANEJO Y ARCHIVO DE DATOS

El tratamiento, la comunicación y la cesión de los datos de carácter personal de todos los sujetos participantes se ajustará a lo dispuesto en la Ley Orgánica 15/1999, de 13 de diciembre, de protección de datos de carácter personal.

En el momento de su registro se asignará a cada paciente un Número de Paciente, que se anotará en el CRD.

Las copias de toda la información pertinente serán conservadas por el investigador y el Promotor, de acuerdo a lo establecido en el Articulo 6 de la Orden SCO/256/2007, del 5 de Febrero, por la que se establecen los principios y las directrices detalladas de buena práctica clínica y los requisitos para autorizar la fabricación o importación de medicamentos en investigación de uso humano, durante un período de mínimo 5 años tras la finalización del estudio o durante un periodo mayor si así lo estableciera la normativa local vigente o cualquier acuerdo entre el Promotor y el Investigador.

El investigador será responsable de conservar la información adecuada acerca de cada paciente de forma que las autoridades sanitarias puedan tener acceso a dicha información si así fuera preciso. Estos registros deberán conservarse de manera confidencial durante el período de tiempo legalmente ordenado por la normativa vigente.

CONDICIONES DE PUBLICACIÓN

El Promotor revisará todas las peticiones de publicación de resultados del presente estudio. El Promotor reconoce la importancia de la difusión de los resultados, y, por tanto, acepta que los Investigadores Principales transmitan los mismos en parte o en su totalidad, siempre y cuando el manuscrito haya sido aprobado antes por él. El Promotor podrá requerir cambios que se consideren necesarios para garantizar la calidad y proteger los derechos de propiedad intelectual. El Investigador Principal entiende y acepta que en determinadas

condiciones, la publicación de resultados deberá retrasarse para garantizar la calidad científica e integridad de los datos, por ejemplo, hasta que se disponga de los resultados en un número de centros que sea representativo del estudio. El Investigador Principal entiende y acepta que el Promotor ostenta la potestad de decidir el momento preciso en que los datos pueden ser difundidos. Mediante la firma del presente protocolo, el Investigador Principal acepta los términos de la política de publicaciones del Promotor y se compromete a respetarlos.

PROCEDIMIENTO PARA LAS MODIFICACIONES DEL PROTOCOLO

Cualquier modificación al protocolo debe documentarse por escrito en forma de enmienda.

Las enmiendas estarán debidamente identificadas por su número de orden cronológico, fechadas y firmadas por el promotor y el investigador.

Si las modificaciones son relevantes, el Promotor deberá solicitar autorización al CEIC de Referencia y a la AEMPS, según establece la normativa vigente.

El Promotor se responsabilizará de llevar un registro de las modificaciones que no cumplan los criterios de modificación relevante, según establece la normativa vigente, y realizar el seguimiento de su aplicación en los centros del ensayo. El registro deberá estar disponible en caso de inspección ó auditoria.

COMITÉ ÉTICO DE INVESTIGACIÓN CLÍNICA (CEIC)

El protocolo y el documento de consentimiento informado serán revisados por un CEIC de referencia adecuadamente constituido. La decisión del CEIC referente al desarrollo del estudio se le facilitará por escrito al investigador; una copia de dicha decisión deberá ser remitida al promotor. Así mismo, los CEICs de los centros participantes evaluarán la Hoja de Información al Paciente y la idoneidad del equipo investigador de su propio hospital.

El promotor presentará los informes requeridos de progreso del estudio al CEIC, y comunicará las sospechas de reacción adversa graves e inesperadas. A la terminación del estudio, el promotor deberá informar de ello al CEIC.

ANALISIS ESTADÍSTICO

El análisis principal del estudio se realizará al finalizar los 6 meses de seguimiento de todos los pacientes.

Tamaño de la muestra y justificación

En una población de estudio similar (ej. Pacientes VIH+ con una carga viral indetectable, es decir < 50copias/mL de ARN viral VIH-1) la puntuación media a nivel basal de la calidad de vida medida con el cuestionario MOS-VIH fue de 61.84 **[24]** Por carecer de la desviación estándar, asumimos para todos los cálculos que la desviación estándar será la puntuación máxima menos la puntuación mínima en la dimensión de Calidad de Vida (ej. 100) dividida por 5, de lo que resulta 20. Esta desviación estándar de 20 es la misma que fue encontrada en pacientes asintomáticos infectados por el VIH, pacientes con síntomas relacionados con SIDA ó pacientes con SIDA y un recuento de células CD4 <100 **[25].** Asumiendo pues, que la Calidad de vida de los pacientes asignados al grupo de control (mantener la terapia antirretroviral actual) se mantendrá sin sufrir modificaciones y que los pacientes asignados al grupo experimental (LPV/r® en monoterapia) mejorará en un 10% (por ej: alcanzarán puntuaciones de 68.02), con una distribución de 1:2, un poder estadístico del 80% y un alfa de dos caras de 0.05, se necesitarían 372 pacientes, 248 aleatorizados en el grupo experimental y 124 aleatorizados para continuar con la terapia antirretroviral actual.
Considerando un 7% de pérdidas de seguimiento, el tamaño muestral final requerido será de 260 pacientes para el grupo experimental y 130 para el grupo control. Un total de 390 pacientes.

Debido a que la triple terapia antirretroviral basada en un IP potenciado con ritonavir (tratamiento control) ya ha sido ampliamente estudiada, por un sentido práctico, se ha considerado asignar el doble de muestra para el tratamiento experimental (1:2, respectivamente) **[24], [26].**

Esta distribución de la muestra (1:2) comparada con la distribución de grupos iguales (1:1) garantiza una mayor seguridad en el contraste principal del estudio ante una posible mayor variabilidad de la respuesta en la calidad de vida en el grupo experimental, ya que en éste grupo se espera un mayor número de pacientes con diarrea al iniciar la nueva terapia **[27]**

Así mismo, va a proporcionará un aumento de la precisión en la estimación de medias y proporciones reduciendo los intervalos de confianza, por ejemplo, más de un 1% en la estimación de proporciones próximas al 50%.

Población del análisis

-

A) Poblaciones por intención de tratar

a.1. Población para el análisis principal

Esta población incluirá a todos los sujetos del grupo control que han sido incluidos en el estudio y a todos los sujetos del grupo experimental que hayan llegado a tomar, al menos, una dosis de Kaletra®. Este análisis va a proporcionar estimaciones del efecto del tratamiento que van a reflejar la práctica habitual con mayor veracidad.

a.2. Población para el análisis secundario

Esta población incluirá a todos los sujetos incluidos en el apartado “a.1.”, pero excluyendo a aquellos pacientes que hayan abandonado el estudio por padecer el evento adverso diarreas. Este análisis va a evaluar el posible aumento de la diferencia de calidad de vida que se sospecha al prever un mayor nivel de abandono en el grupo experimental debido a un evento adverso esperado, por lo que va a proporcionar estimaciones más optimistas de la diferencia entre tratamientos.

B) Población por protocolo (PP):

Esta población incluirá a todos los pacientes que finalicen las 24 semanas de seguimiento.

C) Población de seguridad:

Esta población incluirá a todos los sujetos que hayan recibido al menos una dosis del medicamento del estudio.

Criterio de valoración primario

- Comparar la Calidad de Vida de pacientes desde la situación basal hasta las 24 semanas en los pacientes que inicien monoterapia con comprimidos de LPV/r vs aquellos en triple terapia que contengan cualquier Inhibidor de la Proteasa potenciado (IP) a través del Medical Outcomes Study HIV Health Survey (MOS-HIV) y la escala visual analógica EuroQol 5D (EQ-5D).

Criterio de valoración secundario

- Evaluación de la satisfacción del paciente con el tratamiento de los pacientes VIH+ que inician monoterapia con comprimidos de LPV/r vs triple terapia que contengan

cualquier IP utilizando el cuestionario CESTA (Cuestionario Español de Satisfacción con el tratamiento antirretroviral) desde la visita basal a la semana 24 de estudio.

- Evaluación de la adherencia al tratamiento de los pacientes VIH+ que inician monoterapia con comprimidos de LPV/r vs triple terapia que contengan cualquier IP a través de los cuestionarios GEEMA y una escala visual analógica (EVA).
- Evaluación de la tolerabilidad y seguridad de las pautas en los pacientes VIH+ que inician monoterapia con comprimidos de LPV/r vs triple terapia que contengan cualquier IP.
- Porcentaje de pacientes con una carga viral ARN-VIH <50 copias/mL a las 24 semanas, en los pacientes VIH+ que inician monoterapia con comprimidos de LPV/r vs triple terapia que contengan cualquier IP. Se considerará fallo virológico a una carga viral >500 copias/mL de ARN Viral (HIV1) confirmada a la semana 24 de estudio.
- Cambios en la respuesta inmune en los pacientes VIH+ que inician monoterapia con comprimidos de LPV/r vs triple terapia que contengan cualquier IP medido a través del cambio del recuento de células CD4+ desde la visita basal a la semana 24 de estudio

Métodos estadísticos.

Las pruebas estadísticas a utilizar y el estudio concreto de cada una de las variables incluidas, se describirán detalladamente en el Plan de Análisis Estadístico, que se realizará antes del cierre de la base de datos. No obstante, a continuación se hace una breve descripción del mismo.
Todos los análisis se realizarán con el paquete estadístico SPSS version 15.0 o posterior. Todas las pruebas estadísticas serán bilaterales y con un nivel de significación del 0.05.

Análisis estadísticos.

Las características basales de los grupos en estudio serán comparados utilizando un análisis de varianza (ANOVA) para las variables continuas, proporcionándose la media, mediana, desviación típica y rango, y en caso de variables discretas se presentara la distribución de frecuencias absolutas y porcentajes. En los casos en que fuera preciso, se calcularán los intervalos de confianza al 95%. Las variables discretas se compararán mediante el estadístico Chi-cuadrado.
Para el análisis de la Calidad de vida, la población principal incluirá todos los pacientes de los que se dispongan datos desde el momento basal hasta, como mínimo, una visita posterior a la basal (Ej: la población por intención de tratar).

En caso de abandono del paciente se tomará como medida post-tratamiento la que se obtenga en el momento del abandono. Si no se dispone de valoración post-tratamiento como medida neutral se derivará el valor basal hasta la semana 24 o el momento de abandono (en inglés, Basal Observation Carried Forward).

El principal plazo a estudiar los resultados será a la semana 24 del estudio. Análisis de Covarianza (ANCOVA) será utilizado para la comparación de la Calidad de Vida. El modelo incluirá los valores de las visitas basales de los ítems del cuestionario MOS-HIV, edad, sexo como co-variables, del grupo de control y del de estudio.

Para el análisis de la variable principal se proporcionara el valor de los cuestionarios MOS-HIV y EQ-5D en las diferentes semanas con respecto a basal, para cada grupo de tratamiento.

Se realizaran comparaciones entre los dos grupos de tratamiento, mediante un análisis de covarianza (ANCOVA).

Para el análisis de las variables secundarias se realizará un análisis descriptivo de acuerdo a la distribución de la variable.

Se proporcionará la media, mediana, desviación típica, mínimo, máximo y el intervalo de confianza (95%) para la media de la puntuación de la escala analógica visual (EVA) para y del GEEMA y para cada grupo de tratamiento referentes a la adherencia al tratamiento.

Se proporcionará además la distribución de frecuencias absolutas y porcentaje del grado de percepción en cada una las preguntas para cada grupo de pacientes, según el cuestionario de satisfacción del paciente con el tratamiento (CESTA).

BIBLIOGRAFÍA

1- Hammer SM, Saag MS, Schechter M, Montaner JS, Schooley RT, Jacobsen DM, Thompson MA, Carpenter CC, Fischl MA, Gazzard BG, Gatell JM, Hirsch MS, Katzenstein DA, Richman DD, Vella S, Yeni PG, Volberding PA; International AIDS Society-USA panel.

Treatment for adult HIV infection: 2006 recommendations of the International AIDS Society-USA panel. JAMA. 2006 Aug 16;296(7):827-43.

2- Ammassari A, Trotta MP, Murri R, Castelli F, Narciso P, Noto P, Vecchiet J, D'Arminio Monforte A, Wu AW, Antinori A; AdICoNA Study Group. Correlates and predictors of adherence to highly active antiretroviral therapy: overview of published literature. J Acquir Immune Defic Syndr. 2002 Dec 15;31 Suppl 3:S123-7

3- Van der Valk M, Gisolf EH, Reiss P, Wit FW, Japour A, Weverling GJ, Danner SA; Prometheus study group. Increased risk of lipodystrophy when nucleoside analogue reverse transcriptase inhibitors are included with protease inhibitors in the treatment of HIV-1 infection. AIDS. 2001 May 4;15(7):847-55.

4- Giordano TP, Suarez-Almazor ME, Grimes RM. The population effectiveness of highly active antiretroviral therapy: are good drugs good enough? Curr HIV/AIDS Rep. 2005 Nov;2(4):177-83.

5- Spire B, Marcellin F, Cohen Codar I, et al. Impact of a lopinavir/ritonavir (LPV/r) monotherapy on self-reported side effects and global health perception among antiretroviral-naive patients: 48-week analysis of the MONARK Trial. 8th Annual International Congress on Drug Therapy in HIV Infection (HIV8). Glasgow, UK. November 12-16, 2006. Abstract PL13.3

6-Van der Valk M, Gisolf EH, Reiss P et al. Increased risk of lipodystrophy when nucleoside analogue reverse transcriptase inhibitors are included with protease inhibitors in the treatment of HIV infection. AIDS 2001;15:847-55

7-Falco V, Rodriguez D, Ribera E et al. Severe nucleoside-associated lactic acidosis in human immunodeficiency virus-infected patients: report of 12 cases and review of the literature. Clin Infect Dis. 2002;34:838-46.

8- Norvir Package Insert 2007.

9- Tam and Walmsley 2007.

10- Kempf DJ, Isaacson JD, King MS, Brun SC, Xu Y, Real K, Bernstein BM, Japour AJ, Sun E, Rode RA. Identification of genotypic changes in human immunodeficiency virus protease that correlate with reduced susceptibility to the protease inhibitor lopinavir among viral isolates from protease inhibitor-experienced patients. J Virol 2001; 75: 7462-7469.

11-King MS, Rode R, Cohen-Codar I, Calvez V, Marcelin AG, Hanna GJ, Kempf DJ. Predictive genotypic algorithm for virologic response to lopinavir-ritonavir in protease inhibitor-experienced patients. Antimicrob Agents Chemother 2007; 51: 3067-74.

12- Walmsley S, Bernstein B, King M, Arribas J, Beall G, Ruane P, Johnson M, Johnson D, Lalonde R, Japour A, Brun S, Sun E; M98-863 Study Team. Lopinavir-ritonavir versus nelfinavir for the initial treatment of HIV infection. N Engl J Med. 2002 Jun 27;346 (26):2039-46.

13-JF Delfraissy, P Flandre, C Delaugerre, et al. “MONARK Trial (MONotherapy AntiRetroviral Kaletra): 48-Week Interim Analysis of LPV/r Monotherapy compared to LPV/r + AZT/3TC in Antiretroviral-Naïve Patients” THLB0202. Oral LB. 16th IAC 2006. Toronto (Canada)

14-Cameron DW, da Silva B, Arribas JR, Pulido F, Katner H, Wikstrom K, et al. Significant sparing of peripheral lipoatrophy by HIV treatment with LPV/r + ZDV/3TC induction followed by LPV/r monotherapy compared with EFV + ZDV/3TC. Program and abstracts of the 14th Conference on Retrovirus and Opportunistic Infections; February 25-28, 2007; Los Angeles, California. Abstract 44.

15-Pulido F, Arribas JR, Delgado R, Cabrero E, González-García J, Pérez-Elias MJ, Arranz A, Portilla J, Pasquau J, Iribarren JA, Rubio R, Norton M; OK04 Study Group. Lopinavir-ritonavir monotherapy versus lopinavir-ritonavir and two nucleosides for maintenance therapy of HIV. AIDS 2008; 22: F1-F9.

16-Arribas JR, Pulido F, Delgado R, Lorenzo A, Miralles P, Arranz A, González-García JJ, Cepeda C, Hervás R, Paño JR, Gaya F, Carcas A, Montes ML, Costa JR, Peña JM. Lopinavir/ritonavir as single-drug therapy for maintenance of HIV-1 viral suppression: 48-week results of a randomized, controlled, open-label, proof-of-concept pilot clinical trial (OK Study). J Acquir Immune Defic Syndr. 2005;40: 280-7.

17-Nunes EP, Oliveira MS, Almeida MMTB, Pilotto JH, Ribeiro JE, Faulhaber JC et al. 48-week efficacy and safety results of simplification to single agent lopinavir/ritonavir regimen in patients suppressed below 80 copies/ml on HAART - the KalMo study. Sixteenth International AIDS Conference, Toronto, abstract TuAb0103, 2006.

18-Arribas JR, Pulido F, Delgado R, González-García J, Pérez-Elias MJ, Arranz A, Portilla J, et al OK04 Study Group. Lopinavir-ritonavir monotherapy versus lopinavir-ritonavir and two nucleosides for maintenance therapy of HIV. Ninety-six Week Results of a Randomized, Controlled, Open Label, Clinical Trial (OK04 Study). 11th European AIDS Conference/EACS; October 24-27. 2007. Abstract PS3/1.

19- Recomendaciones de Gesida y Plan Nacional sobre el Sida sobre el tratamiento antirretroviral del adulto (enero, 2008).

20- Badia X, Roset M, Montserrat S, Herdman M, Segura A. [The Spanish version of EuroQol: a description and its applications. European Quality of Life scale]. Med Clin (Barc). 1999;112 Suppl 1:79-85.

21- Condes E, Aguirrebengoa K, Dalmau D, Estrada JM, Force L, Górgolas M, Badia X, Podzamczer D. [Validation of a questionnaire to estimate satisfaction with antiretroviral treatment: CESTA questionnaire] Enferm Infecc Microbiol Clin. 2005 Dec;23(10):586-92.

22- Giordano TP, Guzman D, Clark R, Charlebois ED, Bangsberg DR. Measuring adherence to antiretroviral therapy in a diverse population using a visual analogue scale. HIV Clin Trials. 2004 Mar-Apr;5(2):74-9.

23- Knobel H, Alonso J, Casado JL, Collazos J, Gonzalez J, Ruiz I, Kindelan JM, Carmona A, Juega J, Ocampo A; GEEMA Study Group. Validation of a simplified medication adherence questionnaire in a large cohort of HIV-infected patients: the GEEMA Study. AIDS. 2002 Mar 8;16(4):605-13.

24- Sprinz E, Neto AJ, Bargman E, Green SL, Luo MP, Sylte JR, McMillan FI, King KR, Rode RA, Brun SC, Hanna GJ, Podsadecki TJ. Substitution with lopinavir/ritonavir improves patient-reported outcomes including quality of life in patients who were intolerant to their antiretroviral therapy. HIV Clin Trials. 2006 Nov-Dec;7(6):291-308.

25- Wu AW, Revicki DA, Jacobson D, Malitz FE. Evidence for reliability, validity and usefulness of the Medical Outcomes Study HIV Health Survey (MOS-HIV). Qual Life Res. 1997 Aug;6(6):481-93.

26- Johnson MA, Gathe JC Jr, Podzamczer D, Molina JM, Naylor CT, Chiu YL, King MS, Podsadecki TJ, Hanna GJ, Brun SC. A once-daily lopinavir/ritonavir-based regimen provides noninferior antiviral activity compared with a twice-daily regimen. J Acquir Immune Defic Syndr. 2006 Oct 1;43(2):153-60.

27- Martín Andrés A. y Luna del Castillo J. de D. (1999), Bioestadística para las Ciencias de la Salud, Ediciones Norma.).

APENDICES AL PROTOCOLO

Apéndice 1 - Documento de firma del protocolo

Apéndice 2 - Declaración de Helsinki de la Asociación Médica Mundial

Apéndice 3 - Hoja de Información al Paciente y Consentimiento Informado

Apéndice 4 - Formulario de notificación acontecimiento adversos graves

Apéndice 5 - Clasificación del ACTG de la intensidad de los acontecimientos adversos en adultos

Apéndice 6 – Cuestionarios MOS-HIV, EQ-5D, CESTA; EVA; GEEMA.

APENDICE 1

Documento de firma del protocolo por el investigador principal

Don:

Servicio:

Centro:

Hace constar:

Que ha evaluado el protocolo del ensayo clínico titulado: **“Ensayo clínico, abierto, aleatorizado para comparar la calidad de vida de los pacientes HIV+ que inician monoterapia con comprimidos de LPV/r vs triple terapia que contenga un IP potenciado”**

Código del Protocolo: **SAI-CDV-2009-01**

Nº EudraCT: **2009-014430-25**

Versión 1 del 24 de Julio 2009.

Cuyo promotor es la Sociedad Andaluza de Enfermedades Infecciosas (SAEI)

Que el ensayo clínico respeta las normas éticas aplicables a este tipo de estudios.

Que acepta participar como investigador principal en este ensayo clínico.

Que cuenta con los recursos materiales y humanos necesarios para llevar a cabo el ensayo clínico, sin que ello interfiera en la realización de otro tipo de estudios ni en otras tareas que tiene habitualmente encomendadas.

Que se compromete a que cada sujeto sea tratado y controlado siguiendo lo establecido en el protocolo con dictamen favorable por el Comité Ético de Investigación Clínica y autorizado por la Agencia Española de Medicamentos y Productos Sanitarios.

Que respetará las normas éticas y legales aplicables a este tipo de estudios y seguirá las normas de buena práctica clínica en su realización.

Que los colaboradores que necesita para realizar el ensayo clínico propuesto son idóneos.

En______________________ a_____ de______________ de________

Firmado:

Don/Dña_____________________

Investigador Principal

APÉNDICE 2

DECLARACION DE HELSINKI DE LA ASOCIACION MEDICA MUNDIAL

Principios éticos para las investigaciones médicas en seres humanos

Adoptada por la

18ª Asamblea Médica Mundial, Helsinki, Finlandia, junio 1964

y enmendada por la

29ª Asamblea Médica Mundial, Tokio, Japón, octubre 1975

35ª Asamblea Médica Mundial, Venecia, Italia, octubre 1983

41ª Asamblea Médica Mundial, Hong Kong, septiembre 1989

48ª Asamblea GeneralSomerset West, Sudáfrica, octubre 1996

52ª Asamblea General, Edimburgo, Escocia, octubre 2000

Nota de Clarificación del Párrafo 29, agregada por la Asamblea General de la AMM, Washington 2002

Nota de Clarificación del Párrafo 30, agregada por la Asamblea General de la AMM, Tokio 2004

59ª Asamblea General, Seúl, Corea, octubre 2008

A. INTRODUCCION

1. La Asociación Médica Mundial (AMM) ha promulgado la Declaración de Helsinki como una propuesta de principios éticos para investigación médica en seres humanos, incluida la investigación del material humano y de información identificables.

La Declaración debe ser considerada como un todo y un párrafo no debe ser aplicado sin considerar todos los otros párrafos pertinentes.

2. Aunque la Declaración está destinada principalmente a los médicos, la AMM insta a otros participantes en la investigación médica en seres humanos a adoptar estos principios.

3. El deber del médico es promover y velar por la salud de los pacientes, incluidos los que participan en investigación médica. Los conocimientos y la conciencia del médico han de subordinarse al cumplimiento de ese deber.

4. La Declaración de Ginebra de la Asociación Médica Mundial vincula al médico con la fórmula "velar solícitamente y ante todo por la salud de mi paciente", y el Código Internacional de Ética Médica afirma que: "El médico debe considerar lo mejor para el paciente cuando preste atención médica".

5. El progreso de la medicina se basa en la investigación que, en ultimo término, debe incluir

estudios en seres humanos. Las poblaciones que están sub-representadas en la investigación médica deben tener un acceso apropiado a la participación en la investigación.

6. En investigación médica en seres humanos, el bienestar de la persona que participa en la investigación debe tener siempre primacía sobre todos los otros intereses.

7. El propósito principal de la investigación médica en seres humanos es comprender las causas, evolución y efectos de las enfermedades y mejorar las intervenciones preventivas, diagnósticas y terapéuticas (métodos, procedimientos y tratamientos). Incluso, las mejores intervenciones actuales deben ser evaluadas continuamente a través de la investigación para que sean seguras, eficaces, efectivas, accesibles y de calidad.

8. En la práctica de la medicina y de la investigación médica, la mayoría de las intervenciones implican algunos riesgos y costos.

9. La investigación médica está sujeta a normas éticas que sirven para promover el respeto a todos los seres humanos y para proteger su salud y sus derechos individuales. Algunas poblaciones sometidas a la investigación son particularmente vulnerables y necesitan protección especial. Estas incluyen a los que no pueden otorgar o rechazar el consentimiento por sí mismos y a los que pueden ser vulnerables a coerción o influencia indebida.

10. Los médicos deben considerar las normas y estándares éticos, legales y jurídicos para la investigación en seres humanos en sus propios países, al igual que las normas y estándares internacionales vigentes. No se debe permitir que un requisito ético, legal o jurídico nacional o internacional disminuya o elimine cualquiera medida de protección para las personas que participan en la investigación establecida en esta Declaración.

B. PRINCIPIOS PARA TODA INVESTIGACION MEDICA

11. En la investigación médica, es deber del médico proteger la vida, la salud, la dignidad, la integridad, el derecho a la autodeterminación, la intimidad y la confidencialidad de la información personal de las personas que participan en investigación.

12. La investigación médica en seres humanos debe conformarse con los principios científicos generalmente aceptados y debe apoyarse en un profundo conocimiento de la bibliografía científica, en otras fuentes de información pertinentes, así como en experimentos de laboratorio correctamente realizados y en animales, cuando sea oportuno. Se debe cuidar también del bienestar de los animales utilizados en los experimentos.

13. Al realizar una investigación médica, hay que prestar atención adecuada a los factores que puedan dañar el medio ambiente.

14. El proyecto y el método de todo estudio en seres humanos debe describirse claramente en un protocolo de investigación. Este debe hacer referencia siempre a las consideraciones éticas que fueran del caso y debe indicar cómo se han considerado los principios enunciados en esta Declaración. El protocolo debe incluir información sobre financiamiento, patrocinadores, afiliaciones institucionales, otros posibles conflictos de interés e incentivos para las personas del estudio y estipulaciones para tratar o compensar a las personas que han sufrido daños como consecuencia de su participación en la investigación. El protocolo debe describir los arreglos para el acceso después del ensayo a intervenciones identificadas como beneficiosas en el estudio o el acceso a otra atención o beneficios apropiadas.

15. El protocolo de la investigación debe enviarse, para consideración, comentario, consejo y aprobación, a un comité de ética de investigación antes de comenzar el estudio. Este comité debe ser independiente del investigador, del patrocinador o de cualquier otro tipo de influencia indebida.

El comité debe considerar las leyes y reglamentos vigentes en el país donde se realiza la

investigación, como también las normas internacionales vigentes, pero no se debe permitir que éstas disminuyan o eliminen ninguna de las protecciones para las personas que participan en la investigación establecidas en esta Declaración. El comité tiene el derecho de controlar los ensayos en curso. El investigador tiene la obligación de proporcionar información del control al comité, en especial sobre todo incidente adverso grave. No se debe hacer ningún cambio en el protocolo sin la consideración y aprobación del comité.

16. La investigación médica en seres humanos debe ser llevada a cabo sólo por personas con la formación y calificaciones científicas apropiadas. La investigación en pacientes o voluntarios sanos necesita la supervisión de un médico u otro profesional de la salud competente y calificado apropiadamente. La responsabilidad de la protección de las personas que toman parte en la investigación debe recaer siempre en un médico u otro profesional de la salud y nunca en los participantes en la investigación, aunque hayan otorgado su consentimiento.

17. La investigación médica en una población o comunidad con desventajas o vulnerable sólo se justifica si la investigación responde a las necesidades y prioridades de salud de esta población o comunidad y si existen posibilidades razonables de que la población o comunidad, sobre la que la investigación se realiza, podrá beneficiarse de sus resultados.

18. Todo proyecto de investigación médica en seres humanos debe ser precedido de una cuidadosa comparación de los riesgos y los costos para las personas y las comunidades que participan en la investigación, en comparación con los beneficios previsibles para ellos y para otras personas o comunidades afectadas por la enfermedad que se investiga.

19. Todo ensayo clínico debe ser inscrito en una base de datos disponible al público antes de aceptar a la primera persona.

20. Los médicos no deben participar en estudios de investigación en seres humanos a menos de que estén seguros de que los riesgos inherentes han sido adecuadamente evaluados y de que es posible hacerles frente de manera satisfactoria. Deben suspender inmediatamente el experimento en marcha si observan que los riesgos que implican son más importantes que los beneficios esperados o si existen pruebas concluyentes de resultados positivos o beneficiosos.

21. La investigación médica en seres humanos sólo debe realizarse cuando la importancia de su objetivo es mayor que el riesgo inherente y los costos para la persona que participa en la investigación.

22. La participación de personas competentes en la investigación médica debe ser voluntaria. Aunque puede ser apropiado consultar a familiares o líderes de la comunidad, ninguna persona competente debe ser incluida en un estudio, a menos que ella acepte libremente.

23. Deben tomarse toda clase de precauciones para resguardar la intimidad de la persona que participa en la investigación y la confidencialidad de su información personal y para reducir al mínimo las consecuencias de la investigación sobre su integridad física, mental y social.

24. En la investigación médica en seres humanos competentes, cada individuo potencial debe recibir información adecuada acerca de los objetivos, métodos, fuentes de financiamiento, posibles conflictos de intereses, afiliaciones institucionales del investigador, beneficios calculados, riesgos previsibles e incomodidades derivadas del experimento y todo otro aspecto pertinente de la investigación. La persona potencial debe ser informada del derecho de participar o no en la investigación y de retirar su consentimiento en cualquier momento, sin exponerse a represalias. Se debe prestar especial atención a las necesidades específicas de información de cada individuo potencial, como también a los métodos utilizados para entregar la información. Después de asegurarse de que el individuo ha comprendido la información, el médico u otra persona calificada apropiadamente debe pedir

entonces, preferiblemente por escrito, el consentimiento informado y voluntario de la persona. Si el consentimiento no se puede otorgar por escrito, el proceso para

lograrlo debe ser documentado y atestiguado formalmente.

25.Para la investigación médica en que se utilice material o datos humanos identificables, el médico debe pedir normalmente el consentimiento para la recolección, análisis, almacenamiento y reutilización. Podrá haber situaciones en las que será imposible o impracticable obtener el consentimiento para dicha investigación o podría ser una amenaza para su validez. En esta situación, la investigación sólo puede ser realizada después de ser considerada y aprobada por un comité de ética de investigación.

26. Al pedir el consentimiento informado para la participación en la investigación, el médico debe poner especial cuidado cuando el individuo potencial está vinculado con él por una relación de dependencia o si consiente bajo presión. En una situación así, el consentimiento informado debe ser pedido por una persona calificada adecuadamente y que nada tenga que ver con aquella relación.

27. Cuando el individuo potencial sea incapaz, el médico debe pedir el consentimiento informado del representante legal. Estas personas no deben ser incluidas en la investigación que no tenga posibilidades de beneficio para ellas, a menos que ésta tenga como objetivo promover la salud de la población representada por el individuo potencial y esta investigación no puede realizarse en personas competentes y la investigación implica sólo un riesgo y costo mínimos.

28. Si un individuo potencial que participa en la investigación considerado incompetente es capaz de dar su asentimiento a participar o no en la investigación, el médico debe pedirlo, además del consentimiento del representante legal. El desacuerdo del individuo potencial debe ser respetado.

29. La investigación en individuos que no son capaces física o mentalmente de otorgar

consentimiento, por ejemplo los pacientes inconscientes, se puede realizar sólo si la condición física/mental que impide otorgar el consentimiento informado es una característica necesaria de la población investigada. En estas circunstancias, el médico debe pedir el consentimiento informado al representante legal. Si dicho representante no está disponible y si no se puede retrasar la investigación, el estudio puede llevarse a cabo sin consentimiento informado, siempre que las razones específicas para incluir a individuos con una enfermedad que no les permite otorgar consentimiento informado hayan sido estipuladas en el protocolo de la investigación y el estudio haya sido aprobado por un comité de ética de

investigación. El consentimiento para mantenerse en la investigación debe obtenerse a la brevedad posible del individuo o de un representante legal.

30. Los autores, directores y editores todos tienen obligaciones éticas con respecto a la publicación de los resultados de su investigación. Los autores tienen el deber de tener a la disposición del público los resultados de su investigación en seres humanos y son responsables de la integridad y exactitud de sus informes. Deben aceptar las normas éticas de entrega de información. Se deben publicar tanto los resultados negativos e inconclusos como los positivos o de lo contrario deben estar a la disposición del público..En la publicación se debe citar la fuente de financiamiento, afiliaciones institucionales y conflictos de intereses. Los informes sobre investigaciones que no se ciñan a los principios descritos en esta Declaración no deben ser aceptados para su publicación.

C. PRINCIPIOS APLICABLES CUANDO LA INVESTIGACION MEDICA SE COMBINA CON LA ATENCION MEDICA

31. El médico puede combinar la investigación médica con la atención médica, sólo en la medida en que tal investigación acredite un justificado valor potencial preventivo, diagnóstico o terapéutico y si el médico tiene buenas razones para creer que la participación en el estudio no afectará de manera adversa la salud de los pacientes que toman parte en la investigación.

32. Los posibles beneficios, riesgos, costos y eficacia de toda intervención nueva deben ser

evaluados mediante su comparación con la mejor intervención probada existente, excepto en las siguientes circunstancias:

- El uso de un placebo, o ningún tratamiento, es aceptable en estudios para los que no hay una intervención probada existente.

- Cuando por razones metodológicas, científicas y apremiantes, el uso de un placebo es necesario para determinar la eficacia y la seguridad de una intervención que no implique un riesgo, efectos adversos graves o daño irreversible para los pacientes que reciben el placebo o ningún tratamiento.

Se debe tener muchísimo cuidado para evitar abusar de esta opción.

33. Al final de la investigación, todos los pacientes que participan en el estudio tienen derecho a ser informados sobre sus resultados y compartir cualquier beneficio, por ejemplo, acceso a intervenciones identificadas como beneficiosas en el estudio o a otra atención apropiada o beneficios.

34. El médico debe informar cabalmente al paciente los aspectos de la atención que tienen relación con la investigación. La negativa del paciente a participar en una investigación o su decisión de retirarse nunca debe perturbar la relación médico-paciente.

35. Cuando en la atención de un enfermo las intervenciones probadas han resultado ineficaces o no existen, el médico, después de pedir consejo de experto, con el consentimiento informado del paciente o de un representante legal autorizado, puede permitirse usar intervenciones no comprobadas, si, a su juicio, ello da alguna esperanza de salvar la vida, restituir la salud o aliviar el sufrimiento. Siempre que sea posible, tales intervenciones deben ser investigadas a fin de evaluar su seguridad y eficacia. En todos los casos, esa información nueva debe ser registrada y, cuando sea oportuno, puesta a disposición del público.

APÉNDICE 3

HOJA DE INFORMACIÓN AL PACIENTE Y CONSENTIMIENTO INFORMADO

Hoja de información del paciente

"Ensayo clínico, abierto, aleatorizado para comparar la calidad de vida de los pacientes VIH+ que inician monoterapia con comprimidos de LPV/r vs triple terapia que contenga un IP potenciado"

Promotor: Sociedad Andaluza de Enfermedades Infecciosas (SAEI).

1. OBJETIVO Y PROPÓSITO DEL ESTUDIO

Usted está infectado con el virus de la inmunodeficiencia humana (VIH-1). Su médico le propone participar en un ensayo de investigación clínica en el que se ha planeado incluir aproximadamente 390 pacientes diagnosticados con VIH-1 en España.

En este ensayo, cuyo promotor es la Sociedad Andaluza de Enfermedades Infecciosas (SAEI) le ofrecemos la posibilidad de evaluar como afecta en la calidad de vida mantener un tratamiento de triple terapia (el que Ud. tome actualmente) ó pasar a un único tratamiento antirretroviral (LPV/r, Kaletra®)

Antes de tomar una decisión, debe saber qué se espera de usted durante el ensayo. Lea con atención esta Hoja de Información del Paciente y pregunte a su médico si hay cualquier cosa que le preocupa. Tómese el tiempo que necesite para decidir si le gustaría o no participar en este ensayo.

El propósito de este ensayo es documentar la evolución de la calidad de vida. Se pretende comprobar si existe la posibilidad de mejorar la evolución de los cambios que se producen sobre su calidad de vida debido a su enfermedad y los dos tratamientos que se comparan en el ensayo. Este ensayo investigará un tratamiento basado en cambiar de su tratamiento actual al uso en monoterapia de Kaletra® (LPV/r)

Esta posibilidad de pasar a Kaletra® estando, desde al menos hace 6 meses, con una carga viral indetectable y sin presentar resistencias al gen de la proteasa, ha sido estudiada con anterioridad y ha demostrado ser no inferior a la triple terapia con Kaletra® en cuanto a eficacia virológica e inmunológica.

Nadie puede obligarle a tomar parte en este ensayo. Si desea participar, guarde esta Hoja de Información del Paciente por si la necesitase para hacer una consulta en el futuro. Además, tal como requiere la ley, debe firmar y fechar el formulario de consentimiento informado adjunto.

La participación en este ensayo clínico es voluntaria. Aun cuando acceda a participar ahora, usted es libre de cambiar de opinión y abandonarlo posteriormente sin ninguna penalización ni pérdida de los beneficios a los que, en cualquier caso, tiene derecho. Por otra parte, su médico puede decidir retirarle del estudio en cualquier momento si, de acuerdo con su experiencia y criterio médico, considera que se beneficiaría de la interrupción del tratamiento del ensayo.

El tratamiento de su enfermedad o la continuación del tratamiento una vez que este ensayo haya finalizado son aspectos que usted tratará con su médico, quien le informará en detalle sobre las opciones de tratamiento disponibles (otros fármacos con diferentes mecanismos de acción).

2. MEDICACIÓN DEL ENSAYO

Este ensayo clínico sigue lo que se conoce como un diseño de ensayo aleatorizado y comparativo. Ensayo comparativo significa que se compararán dos grupos de tratamiento. Con el fin de determinar si la pauta posológica basada en su tratamiento actual o Kaletra® en monoterapia muestran diferencias en cuanto a su calidad de vida. Antes del comienzo del ensayo, los participantes se asignarán al azar (una acción similar a lanzar una moneda al aire) al grupo de tratamiento con la pauta posológica que recibe actualmente o al grupo de tratamiento con Kaletra® en monoterapia.

Si usted cumple todos los requisitos necesarios para ser incluido en el presente ensayo y accede a participar, puede recibir una de las siguientes pautas posológicas:

- ☞ **Cambiar de su tratamiento antirretroviral actual a Kaletra® en monoterapia.**
  Kaletra contiene 200 mg de Lopinavir y 50 mg de Ritonavir, dos fármacos antirretrovirales que ya están autorizados. La dosis de Kaletra es 2 comprimidos dos veces al día.

- ☞ **Permanecer con la pauta posológica actual sin cambios.**
  La medicación será administrada según indica su propia ficha técnica.

Posibles riesgos

Riesgos relacionados con las intervenciones a estudio:

En el momento actual el tratamiento de la infección por VIH requiere la administración de al menos tres medicamentos antirretrovirales. Cuando usted sea asignado al grupo de simplificación de tratamiento y cambie los fármacos que está tomando actualmente existe riesgo de perder el control de la infección por VIH y que su carga viral pueda volver a hacerse detectable. Asimismo existe el riesgo de que su virus se haga resistente al tratamiento con Lopinavir/ritonavir. Por este motivo en el caso de hallarse una carga viral detectable, se le repetirá la determinación de carga viral y si se confirma una subida a más de 500 copias se realizará un test de resistencia que permita aconsejarle el mejor tratamiento para usted.

Otros posibles riesgos relacionados con la medicación de estudio:

- Lopinavir/ritonavir: Los efectos secundarios más frecuentemente descritos en pacientes tratados con Lopinavir/ritonavir (Kaletra®) son: diarrea, náuseas, aumento de los lípidos (colesterol y triglicéridos) plasmáticos, posible aumento de enzimas hepáticas y riesgo de hepatitis.

Durante el tratamiento con Kaletra no debe tomar ninguno de los medicamentos siguientes porque pueden causar problemas graves o incluso la muerte:

Antihistamínicos (medicamentos para tratar la alergia): terfenadina (Cyater®, Rapidal®, Ternadin®)

Sedantes/hipnóticos (medicamentos para tratar alteraciones del sueño): midazolam (Dormicum®) o triazolam (Halcion®)

Antimicrobianos (antibióticos, medicamentos para tratar infecciones bacterianas): rifampicina (Rifaldin®, Rifater®)

Fármacos para la migraña (enfermedad del sistema nervioso caracterizada por jaquecas intensas, acompañada de otros sintomas, como trastornos en la visión, etc.): ergotamina (Cafergot®,Hemicraneal®) dihidroergotamina (Tonopan®), y metilergonavina (Methergin®)

Neurolépticos (medicamentos para tratar la psicosis y otras alteraciones mentales): pimozida (Orap®)

Antieméticos (medicamentos para evitar el vómito)/antidiarreicos: cisaprida (Prepulsid®, Arcasin®)

Fármacos hipolipemiantes (medicamentos para disminuir los niveles de lípidos (colesterol) en sangre): lovastatina (Mevacor®) o simvastatina (Zocor®)

Preparados herbales (preparados a base de hierbas): hierba de San Juan (hipérico)

Antiarrítmicos (medicamentos para tratar las alteraciones del ritmo cardiaco): propafenona (Rytmonorm®) y flecainida (Apocard®)

Es importante que comente con el médico o un miembro de su personal los otros medicamentos que está tomando, porque en algunos casos hay que cambiar la dosis de esos medicamentos si se toman con lopinavir/ritonavir.

Se compromete usted a no utilizar fármacos (incluidos el alcohol y los medicamentos de venta con y sin receta médica, preparados de herbolario, suplementos vitamínicos o drogas ilegales) distintos de lopinavir/r sin la autorización del médico del estudio. El tratamiento con fármacos distintos de Lopinavir/ritonavir puede provocar reacciones graves e incluso potencialmente letales.

--Abacavir/Lamivudina: Aunque usted esté tolerando el tratamiento actual, los fármacos nucleósidos (abacavir y lamivudina) podrían originar efectos tóxicos de varios tipos: anemia, inflamación de los nervios y pérdida de grasa corporal entre otros.

También podría sufrir efectos secundarios desconocidos o no comunicados hasta ahora.

Debido a su efecto sobre la carga viral, debe comunicar a su médico o personal del estudio si ha recibido una vacuna en el mes previo al estudio o si le vacunan en cualquier momento durante el estudio.

Riesgos en poblaciones especiales: Mujeres en edad fértil:

En la actualidad el uso de Lopinavir/ritonavir es el IP de elección durante el embarazo, pero su uso en monoterapia no es seguro durante el embarazo. Por tanto, si usted es una mujer en edad fértil, sólo se le incluirá en el estudio si no está embarazada y utiliza un método anticonceptivo de barrera fiable (a juicio del médico del estudio), o si no mantiene actividad sexual. Se realizará una prueba de embarazo en el momento basal/día 1. No recibirá el tratamiento del estudio hasta disponer de una prueba de embarazo en orina negativa. A criterio del médico del estudio, podrán realizarse pruebas de embarazo adicionales durante el estudio. Si se queda embarazada durante este estudio, deberá comunicarlo al médico del estudio inmediatamente y podría suspenderse su participación en él.

En el caso de que se produzca un embarazo, se pondrán a su disposición la atención médica y los medios necesarios en consonancia con la decisión que Vd. adopte sobre su embarazo. Además, posteriormente se asegurará el seguimiento del bebé hasta 6 meses

después del parto, aunque parte de este seguimiento ya se realizaría fuera del contexto del ensayo clínico.

Otros riesgos:

Los riesgos asociados a la extracción de sangre son: dolor, hematomas, sangrado u otras molestias en el lugar de extracción de la sangre. Anemia, desmayo o infección en el punto de extracción de la sangre pueden producirse en raras ocasiones. Se tomarán las precauciones adecuadas para reducir al mínimo estos riesgos.

Información nueva:

Se le comunicará por escrito cualquier novedad importante en relación con el estudio que pueda afectar a su seguridad o modificar su decisión de participar en el estudio. Si después de recibir la información decide continuar en este estudio, se le pedirá que firme un consentimiento informado nuevo (revisado) para documentar que ha recibido esta nueva información.

3. CRITERIOS DE ELEGIBILIDAD

Para poder participar en el ensayo, su médico verificará todos los criterios de inclusión / exclusión y le comunicará si puede o no participar de acuerdo con el punto de vista clínico. Después, usted decidirá libremente si desea participar una vez facilitada toda la información referente al ensayo.

4. PROCEDIMIENTOS DEL ENSAYO

La duración total del ensayo es de 24 semanas. Si acepta tomar parte en el ensayo y da su consentimiento informado por escrito (con su firma y la fecha del día de aceptación), asistirá a una visita de selección, una visita inicial (en la que se inicia el seguimiento y comenzará con la medicación asignada ó continuará con la que está tomando actualmente) y una visita en las semanas 12, y 24 desde la visita de inicial. En caso de haber iniciado Kaletra® en monoterapia volverá a la 4ª semana tras la visita inicial para control virológico y de tolerancia. Estas visitas no representan un cambio en la frecuencia del seguimiento que normalmente lleva a cabo su médico, exceptuando la visita de la semana 4.

En las visitas mencionadas anteriormente, se realizarán las siguientes evaluaciones:

Visita de selección

En esta visita, si ha decidido tomar parte en el ensayo, debe firmar un documento llamado Formulario de Consentimiento Informado que indica que usted quiere participar en el ensayo voluntariamente una vez que se le ha facilitado toda la información solicitada. A continuación, se realizarán algunas preguntas referentes a su historia clínica y se realizará una exploración física y análisis de sangre para confirmar que cumple todos los criterios requeridos para participar en el ensayo.

Si usted es mujer en edad fértil, se someterá a una prueba de embarazo (análisis de orina.)

Visita inicial

Una vez disponibles los resultados de la visita de selección, se le citará para la visita inicial del estudio. En esta visita, se realizarán de nuevo una exploración física y análisis de sangre y se le informará del tratamiento anti-VIH asignado. Si se dispone de resultados recientes de análisis de sangre (en el plazo de dos meses anteriores) su médico puede decidir realizar las visitas de selección e inicial en una única visita.

En esta visita además, deberá cumplimentar un cuestionario sobre la calidad de vida que Ud. percibe actualmente, la sensación de satisfacción que tiene con el tratamiento que recibe y su nivel de adherencia al tratamiento.

Visitas de seguimiento y final

Acudirá a una visita de seguimiento a la semana 12, y a una visita final, a las 24 semanas tras la visita inicial. . En caso de haber iniciado Kaletra® en monoterapia volverá a la 4ª semana tras la visita inicial para control virológico y de tolerancia.

En algunas de estas visitas tendrá que rellenar varios cuestionarios sobre la calidad de vida que Ud. percibe actualmente, la sensación de satisfacción que tiene con el tratamiento que recibe y su nivel de adherencia al tratamiento.

Además, en dichas visitas serán necesarias muestras de sangre para análisis de laboratorio habituales. Se especifica que la cantidad de sangre por visita será aproximadamente de 15 ml y todas las muestras que se obtengan a raíz de este Ensayo Clínico sólo se usarán para los fines específicos del estudio.

En cada visita, su médico le preguntará si ha aparecido algún signo o síntoma inusual desde la última visita. Además, se le preguntará si ha tomado alguna medicación que sea diferente de la medicación del ensayo desde la última visita.

Su médico le recetará su medicación y le citará para la siguiente visita.

Adicionalmente en la visita final se someterá a una evaluación final y su médico decidirá si debe continuar con la medicación utilizada durante el ensayo, o si es necesario un cambio de tratamiento.

Debe usted saber que hay algunos medicamentos que pueden estar contraindicados con el tratamiento del ensayo y **no debe iniciar ningún tratamiento** sin consultarlo previamente con su médico del ensayo.

5. RIESGOS Y BENEFICIOS

Antes de diseñar este ensayo clínico, médicos e investigadores han sopesado cuidadosa y meticulosamente los beneficios del tratamiento del estudio frente a los riesgos potenciales. Para proteger al paciente, este ensayo se realizará de total conformidad con los principios de la Asociación Médica Mundial, de acuerdo con las normas, directivas y reglas de la Unión Europea y en estricto cumplimiento de las leyes españolas sobre Especialidades Farmacéuticas y la Ley Española de Protección de Datos.

Comunique a su médico cualquier efecto secundario:
Seguidamente le resumimos los más efectos secundarios más frecuentes relacionados con el fármaco en estudio:

Efectos secundarios muy frecuentes
(Pueden afectar a más de 1 de cada 10 pacientes tratados)

- Dolor de cabeza, diarrea, sensación de mareo (náuseas).

Efectos secundarios frecuentes
(Pueden afectar a de 1 a 10 de cada 100 pacientes tratados)

- Vómitos, dolor abdominal, dolor de estómago, deposiciones anormales.
- Dificultad para dormir, sueños anómalos, fiebre, malestar general.

- Sensación de hormigueo.
- Tos, síntomas nasales.
- Problemas con la digestión que dan como resultado molestias tras las comidas, flatulencia.
- Exantemas, que pueden ser reacciones alérgicas, picor, cambios en el color de la piel.
- Otras reacciones alérgicas.
- Sensación de debilidad

Las pruebas también pueden mostrar:

- bajo recuento de glóbulos blancos (un reducido recuento de glóbulos blancos puede hacerle más propenso a la infección), bajo recuento de glóbulos rojos (anemia)
- aumento azúcar en la sangre
- problemas hepáticos y pancreáticos

Efectos secundarios poco frecuentes
(Pueden afectar a menos de 1 en cada 1.000 pacientes tratados)

- dolor en el abdomen (estómago) producido por inflamación del páncreas
- cambios en la orina y dolor de espalda producido por problemas renales

Las pruebas también pueden mostrar:

- acidosis láctica (exceso de ácido láctico en la sangre, un efecto secundario grave que puede ser mortal). Los siguientes efectos secundarios pueden ser signos de acidosis láctica:
    - respiración profunda y rápida
    - somnolencia
    - sentirse mareado (náuseas), vómitos y dolor de estómago

Si cree que tiene los síntomas anteriores, póngase en contacto con su médico inmediatamente.

Otros posibles efectos

El tratamiento antirretroviral de combinación puede cambiar la forma de su cuerpo, al cambiar la forma en que se distribuye la grasa corporal. Puede perder grasa de las piernas, brazos y cara; aumentar grasa alrededor del abdomen (estómago) y órganos internos; aumentar el tamaño de los pechos o bultos de grasa en la parte posterior del cuello

(“joroba de búfalo”). La causa y los efectos a largo plazo de estos cambios todavía no se conocen.

El tratamiento antirretroviral de combinación también puede producir hiperlipidemia (aumento de grasas en la sangre) y resistencia a la insulina. Su médico realizará pruebas para determinar estos cambios.

Recuerde, si observa cualquier otro efecto secundario, comuníqueselo a su médico del ensayo.

En cualquier momento durante el ensayo, puede formular cualquier pregunta con respecto a los riesgos potenciales y/o conocidos del ensayo.

Los pacientes tratados con los medicamentos que se van a utilizar en el ensayo o cualquier otro tratamiento antirretroviral pueden continuar desarrollando infecciones oportunistas y otras complicaciones relacionadas con el VIH.

No se ha demostrado que las pautas posológicas antirretrovirales actuales, que incluyen los medicamentos utilizados en este estudio, eviten el riesgo de transmisión del VIH a otras personas por la vía sexual o por contaminación sanguínea y, por tanto, es obligatorio el uso continuo de precauciones adecuadas.

En caso de embarazo, debe informar a su médico inmediatamente.

En caso de tener cualquier problema tras comenzar a recibir la medicación del ensayo, debe ponerse en contacto con su médico inmediatamente.

Si tiene alguna duda con respecto a los efectos adversos de la medicación del ensayo, no dude en ponerse en contacto con su médico o en revisar los prospectos correspondientes.

Se informará a su médico de medicina general / médico de familia de su participación en el estudio con su consentimiento.

Su participación en el ensayo puede beneficiarle porque podría experimentar una mejoría en cuanto a su calidad de vida por la posibilidad de tener menos acontecimientos adversos por el tratamiento que se le asigne, pero debe saber que su participación puede no

aportarle ningún beneficio, aunque sí puede ayudar a que otros pacientes VIH se beneficien del mismo. En total conformidad con las normas y reglas aplicables a los estudios de investigación clínica, su médico monitorizará su estado cuidadosamente con respecto a la eficacia del tratamiento y a los posibles efectos secundarios.

6. TRATAMIENTOS ALTERNATIVOS

Hay otras opciones de tratamiento antirretroviral que puede tratar con su médico. En la actualidad se dispone de un gran número de medicamentos que están autorizados para el tratamiento de su enfermedad, que pertenecen principalmente a cuatro familias:

- Análogos nucleótidos de la transcriptasa inversa (ANITI): Zidovudina (AZT), Didanosina (ddI), Zalcitabina (ddC), Estavudina (d4T), Lamivudina (3tC), Abacavir (ABA)
- Análogos nucleótidos de la transcriptasa inversa (ANTITI) : Tenofovir (PMPA)
- No análogos de los nucleósidos de la transcriptasa inversa (NNITI): Nevirapina, Delavirdina, Efavirenz
- Inhibidores de la proteasa (IP): Saquinavir, Ritonavir, Indinavir, Nelfinavir, Amprenavir, Lopinavir.

Muchos de estos fármacos tienen numerosas interacciones y esto hace que sea importante conocer sus características farmacológicas y contraindicaciones. Por este motivo, en el caso de participación en el ensayo y necesidad de retirada debido a fracaso del tratamiento, intolerancia o toxicidad, su médico le informará sobre los tratamientos alternativos que se pueden utilizar para el tratamiento de su enfermedad.

7. ASPECTOS ÉTICOS

Este ensayo se ha aprobado por un Comité Ético de Investigación Clínica de referencia, y se ha revisado también por los Comités Éticos de todos los hospitales participantes. Además, el estudio se ha presentado y ha sido revisado y aprobado por las autoridades sanitarias competentes.

8. CONFIDENCIALIDAD DE LOS DATOS

Los datos que se recojan en este estudio son confidenciales, salvo que la ley requiera su relevación. La información de este estudio será revisada y remitida a los organizadores del estudio, las autoridades sanitarias y al Comité Ético de Investigación Clínica del centro correspondiente para llevar a cabo sus obligaciones tal y como la ley lo permita. Estas entidades podrán también inspeccionar su historia clínica, que le identifica a usted, y el consentimiento informado que ha firmado. Si es necesario, podrán comunicar la información (exceptuando los datos de identificación de los pacientes) a sus socios o autoridades sanitarias de otros países de la UE, Estados Unidos u otros países que no pertenecen a la UE. Sin embargo, su confidencialidad será protegida de acuerdo con las normas estipuladas en la Ley Orgánica 15/1999 de Protección de Datos y el RD 994/99, y la cesión de los datos se realizará cumpliendo la legislación vigente. Los resultados de este estudio de investigación pueden presentarse en congresos o publicarse; sin embargo, no se incluirá su identidad en esas presentaciones.

Como medida de seguridad para garantizar su confidencialidad, el tratamiento, comunicación y cesión de los datos de carácter personal de los sujetos participantes se ajustará a lo dispuesto en la citada Ley 15/1999. Según dicha Ley, el consentimiento para el tratamiento de sus datos personales y para su cesión es revocable. Ud. puede ejercer en cualquier momento el derecho de acceso, rectificación y cancelación de los datos, dirigiéndose al médico del estudio, quien informará al promotor y representantes. Si decide retirar el consentimiento, sólo se guardará la información obtenida hasta ese momento pero no se obtendrá nueva información.

Además, este estudio se llevará a cabo de acuerdo con la Ley 29/2006, con el Real Decreto 223/2004 y demás legislación aplicable relativa a ensayos clínicos con medicamentos y siguiendo la Declaración de Helsinki y la Guía de Buenas Prácticas Clínicas (CPMP/ICH/135/95) internacionales.

9. BASES PARA LA PARTICIPACIÓN

Su participación en este ensayo es totalmente voluntaria. Usted es libre de cambiar de opinión y abandonarlo en un momento posterior sin ninguna penalización ni pérdida de los beneficios a los que, en cualquier caso, tiene derecho. Si finalmente Ud. decide participar

en el estudio, el equipo investigador recibirá una compensación económica por el trabajo adicional que supone su seguimiento y control.

El Dr………………………….. le dará a conocer las alternativas de tratamiento que considere apropiadas para usted y le informará del procedimiento para la interrupción del tratamiento del ensayo sin problemas.

Debe saber que el Dr …………………. puede detener su participación en el ensayo clínico en caso de que usted no haya seguido las instrucciones del estudio, en caso de que la retirada sea lo más beneficioso para usted o si el promotor cancela el estudio. El promotor se compromete a comunicar cualquier información importante que se descubriera sobre la medicación del estudio o la enfermedad durante el transcurso del ensayo clínico, la cuál se le informará inmediatamente y se tomará la decisión de si desea o no continuar o retirarse del estudio.

10. SEGURO DEL ESTUDIO

Debe saber que hay un seguro de responsabilidad civil contratado por el promotor con la compañía HDI Hannover Internacional (España) Seguros y Reaseguros, S.A., Delegación para España, con domicilio en c/ Luchana, 23 3ª 28010 (póliza de seguros con número 130/001/006201) para la cobertura de posibles lesiones y daños, tal como requiere la ley en vigor (Real Decreto 223/04).

11. INFORMACIÓN ADICIONAL

Este ensayo se lleva a cabo bajo la dirección del Dr ……………………………..

Puede consultar con él/ella cuando lo desee. Si siente cualquier efecto secundario, si tiene dudas acerca de cualquier aspecto del estudio o de sus derechos como paciente, no dude en llamar a este número de teléfono …………………… o al número …………………………………

FORMULARIO DE CONSENTIMIENTO INFORMADO POR ESCRITO

"Ensayo clínico, abierto, aleatorizado para comparar la calidad de vida de los pacientes HIV+ que inician monoterapia con comprimidos de LPV/r vs triple terapia que contenga un IP potenciado"

Yo, (escriba el nombre completo)____-

al firmar esta hoja en la parte inferior confirmo que:

1. He leído la hoja de información al paciente del estudio de investigación arriba indicado. También he recibido por parte del Dr. ________________________ una explicación de lo que trata el estudio su propósito y su duración; los posibles efectos y riesgos de la medicación, y en qué consistirá mi participación.

   Si ☐ **No** ☐

2. Se me ha permitido hacer preguntas sobre el estudio y estoy enteramente satisfecho/a con las respuestas y explicaciones que me han proporcionado.

   Si ☐ **No** ☐

3. Me han dado tiempo suficiente para leer la información de este formulario de consentimiento, poder consultarlo con otras personas y decidir si quiero o no participar en el estudio.

   Si ☐ **No** ☐

4. Sé que mi participación en este estudio es voluntaria y puedo abandonar el estudio en cualquier momento sin que mi cuidado médico o derechos legales se vean afectados.

   Si ☐ **No** ☐

5. Me pondré en contacto inmediatamente con el investigador del estudio si padezco cualquier síntoma inesperado o raro durante el estudio.

   Si ☐ **No** ☐

6. Entiendo que podría retirarme del ensayo cuándo lo desee, sin explicar el motivo y sin que afecte a mi atención médica.

   Si ☐ **No** ☐

7. He leído que tengo derecho a acceso, rectificación y cancelación de los datos, en cualquier momento del ensayo.

   Si ☐ **No** ☐

Por la presente, doy libremente mi consentimiento para tomar parte en el ensayo.

Firma del participante **Fecha**

Firma del Investigador **Fecha**

FORMULARIO DE CONSENTIMIENTO ORAL ANTE TESTIGOS

"Ensayo clínico, abierto, aleatorizado para comparar la calidad de vida de los pacientes HIV+ que inician monoterapia con comprimidos de LPV/r vs triple terapia que contenga un IP potenciado"

Yo, (nombre completo del testigo) .... en mi condición de... (relación con el participante), declaro bajo mi responsabilidad que .... (nombre completo del participante)

- Ha leído la hoja de información que le han facilitado.
- Ha podido formular preguntas acerca del ensayo.
- Ha recibido respuestas satisfactorias a todas sus preguntas.
- Ha recibido suficiente información sobre el ensayo.
- Ha hablado con el Dr. .... (nombre del investigador)
- Ha entendido que su participación es voluntaria.
- Ha entendido que puede retirarse del ensayo:
  1. Cuando lo desee.
  2. Sin explicar el motivo.
  3. Sin que afecte a su atención médica.

Y ha expresado libremente su conformidad de participar en este estudio.

Firma del testigo **Fecha**

Firma del Investigador **Fecha**

APÉNDICE 4

Formulario de notificación acontecimiento adversos graves

Se adjunta documento aparte

APÉNDICE 5

CLASIFICACIÓN DEL ACTG DE LA INTENSIDAD DE LOS ACONTECIMIENTOS ADVERSOS EN ADULTOS

Se adjunta documento aparte.

ANEXO 1

GRAFICA DE MANEJO DE PACIENTES CON FALLO VIROLOGICO

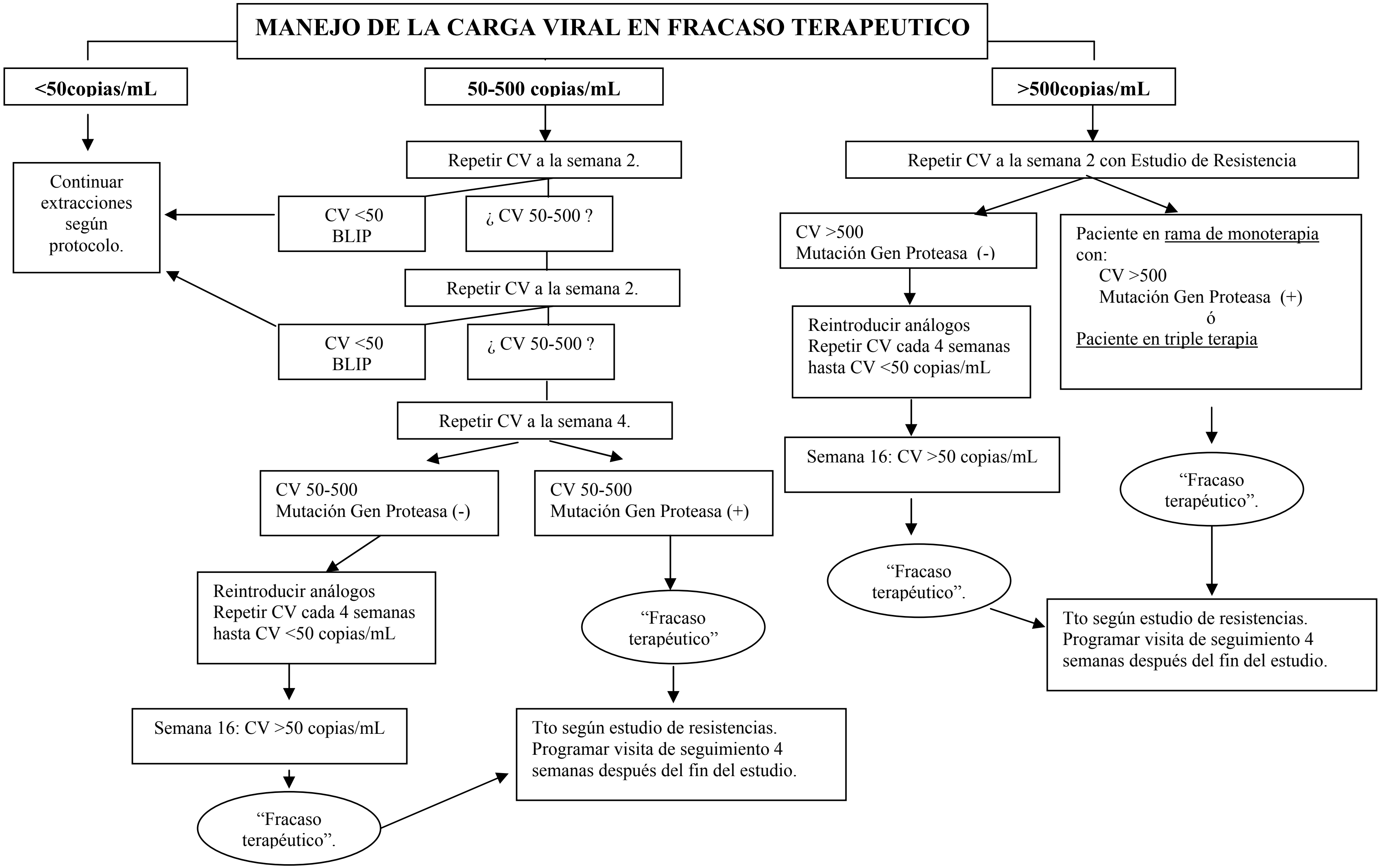
MANEJO DE LA CARGA VIRAL EN FRACASO TERAPEUTICO
<50copias/mL
50-500 copias/mL
>500copias/mL
Continuar extracciones según protocolo.
Repetir CV a la semana 2.
CV <50 BLIP
¿ CV 50-500 ?
Repetir CV a la semana 2.
CV <50 BLIP
¿ CV 50-500 ?
Repetir CV a la semana 4.
CV 50-500 Mutación Gen Proteasa (-)
CV 50-500 Mutación Gen Proteasa (+)
Reintroducir análogos Repetir CV cada 4 semanas hasta CV <50 copias/mL
"Fracaso terapéutico"
Semana 16: CV >50 copias/mL
Tto según estudio de resistencias. Programar visita de seguimiento 4 semanas después del fin del estudio.
"Fracaso terapéutico".
Repetir CV a la semana 2 con Estudio de Resistencia
CV >500 Mutación Gen Proteasa (-)
Paciente en rama de monoterapia con: CV >500 Mutación Gen Proteasa (+) ó Paciente en triple terapia
Reintroducir análogos Repetir CV cada 4 semanas hasta CV <50 copias/mL
Semana 16: CV >50 copias/mL
"Fracaso terapéutico".
"Fracaso terapéutico".
Tto según estudio de resistencias. Programar visita de seguimiento 4 semanas después del fin del estudio.

ANEXO 2:
PROCEDIMIENTO DE RE-ETIQUETADO DE MEDICACIÓN PARA USO EXCLUSIVO DE ENSAYO CLÍNICO QoLKAMON

Se adjunta documento aparte.

ANEXO 3:

CUESTIONARIOS DE EVALUACIÓN DE CALIDAD DE VIDA, ADHERENCIA AL TRATAMIENTO Y SATISFACCIÓN CON EL TRATAMIENTO

CUESTIONARIO PARA LA EVALUACIÓN DE LA CALIDAD DE VIDA

Encuesta de Salud MOS-VIH

INSTRUCCIONES PARA El COORDINADOR DEL ESTUDIO:

En los cuestionarios siguientes se interroga al paciente sobre numerosos aspectos de su estado de salud y asistencia sanitaria. Deben facilitarse al paciente antes de la exploración física, preferiblemente en un ambiente tranquilo (p. ej., en la sala de exploración o en otro consultorio).

Es importante familiarizarse con el contenido y el formato de los cuestionarios antes de entregárselos a los participantes en el estudio. En la primera visita, indique al paciente lo siguiente:

> "Le rogamos que responda a algunas preguntas sobre cómo se siente y lo que es capaz de hacer. Sus respuestas nos ayudarán a comprender los efectos de la medicación que está tomando. Le agradeceríamos que rellenara estos cuestionarios."

A continuación, debe explicar brevemente el formato de las preguntas y cómo cumplimentarlas. El paciente deberá cumplimentar los cuestionarios antes de que se realicen la historia clínica y la exploración física y se determinen las constantes vitales.

Los cuestionarios son muy breves y no deben tardarse más de 15-30 minutos en cumplimentarlos. Antes de entregárselos al paciente, rellene el encabezado y SEPARE ESTA PÁGINA.

Recoja los cuestionarios cumplimentados antes de la exploración clínica. Antes de continuar, revíselos en busca de posibles omisiones. Si el participante ha dejado alguna pregunta sin contestar, indíqueselo y haga que subsane las omisiones.

COMPLETE, POR FAVOR, LOS APARTADOS SIGUIENTES DESPUÉS DE QUE EL PACIENTE HAYA CUMPLIMENTADO LOS CUESTIONARIOS, O DESPUÉS DE COMPROBAR QUE ELLO NO ES POSIBLE:

1. ¿Cómo se cumplimentaron los cuestionarios?................1 - Los cumplimentó el participante en el estudio ☐
   2 - Mediante entrevista personal con usted
   3 - Mediante entrevista telefónica
   4 - No se cumplimentaron
   5 - Otros

   Si la respuesta es Otros, especifique ____________________

2. Si la respuesta es 2 ó 4, indique el motivo (1 = Sí, 2 = No)

   Rechazo inicial del paciente .... ☐

   Nivel de lectura del paciente insuficiente.............................. ☐

   Falta de tiempo .... ☐

   El paciente no disponía de sus gafas .... ☐

   Otro motivo.. ☐

   Si la respuesta es Otro motivo, especifique cuál ____________________

Encuesta de Salud MOS-VIH (continuación)

<u>INSTRUCCIONES PARA EL PACIENTE</u>: Conteste a las siguientes preguntas marcando con una "X" la casilla que proceda.

1. En general, diría usted que su estado de salud es: (Marque sólo una respuesta)

Excelente	1 ☐
Muy bueno	2 ☐
Bueno	3 ☐
Regular	4 ☐
Malo	5 ☐

2. ¿Cuánto dolor corporal ha sentido en general durante las últimas 4 semanas? (Marque sólo una respuesta)

Ninguno	1 ☐
Muy leve	2 ☐
Leve	3 ☐
Moderado	4 ☐
Intenso	5 ☐
Muy intenso	6 ☐

3. Durante las últimas 4 semanas, ¿en qué medida interfirió el dolor en su trabajo normal (o en sus actividades normales, incluidos el trabajo fuera de casa y las tareas domésticas)? (Marque sólo una respuesta)

Nada en absoluto	1 ☐
Algo	2 ☐
Moderadamente	3 ☐
Bastante	4 ☐
Muchísimo	5 ☐

Encuesta de Salud MOS-VIH (continuación)

4. Las preguntas siguientes se refieren a actividades que podría hacer durante un día normal. ¿Le limita su salud actual para estas actividades? Si es así, ¿en qué medida?

	**(Marque sólo una casilla en cada línea)**	**SÍ, me limita mucho (1)**	**SÍ, me limita algo (2)**	**NO, no me limita nada (3)**
a.	Actividades enérgicas, como levantar objetos pesados, correr o participar en deportes que exijan esfuerzo intenso	☐ (1)	☐ (2)	☐ (3)
b.	Actividades moderadas, como mover una mesa, cargar con la compra o jugar a los bolos	☐ (1)	☐ (2)	☐ (3)
c.	Caminar cuesta arriba o subir (varios tramos de escaleras)	☐ (1)	☐ (2)	☐ (3)
d.	Agacharse, levantarse o inclinarse	☐ (1)	☐ (2)	☐ (3)
e.	Caminar una manzana	☐ (1)	☐ (2)	☐ (3)
f.	Comer, vestirse, bañarse o utilizar el inodoro	☐ (1)	☐ (2)	☐ (3)

5. ¿Le impide su estado de salud trabajar, hacer tareas domésticas o ir a la escuela?

(Marque sólo una respuesta)

Sí 1 ☐

No 2 ☐

6. ¿Ha sido incapaz de realizar determinados tipos o cantidades de trabajo, tareas domésticas o escolares debido a su estado de salud?

(Marque sólo una respuesta)

Sí 1 ☐

No 2 ☐

Encuesta de Salud MOS-VIH (continuación)

En cada una de las siguientes preguntas, marque la casilla de la respuesta que <u>mejor refleje</u> cómo se ha sentido <u>durante las últimas 4 semanas</u>.

	Todo el tiempo<br>1	La mayor parte del tiempo<br>2	Gran parte del tiempo<br>3	Parte del tiempo<br>4	En algún momento<br>5	En ningún momento<br>6
7. Durante las últimas 4 semanas, ¿qué parte del tiempo ha limitado su estado de salud sus actividades sociales (como visitar a los amigos o familiares)?	☐	☐	☐	☐	☐	☐
8. Durante las últimas 4 semanas, ¿qué parte del tiempo:						
a. Se ha sentido muy nervioso?	☐	☐	☐	☐	☐	☐
b. Se ha sentido tranquilo y calmado?	☐	☐	☐	☐	☐	☐
c. Se ha sentido triste y desanimado?	☐	☐	☐	☐	☐	☐
d. Se ha sentido feliz?	☐	☐	☐	☐	☐	☐
e. Se ha sentido tan decaído que nada podía animarle?	☐	☐	☐	☐	☐	☐

Encuesta de Salud MOS-VIH (continuación)

En cada una de las siguientes preguntas, marque la casilla de la respuesta que mejor refleje cómo se ha sentido durante las últimas 4 semanas.

	Todo el tiempo<br>1	La mayor parte del tiempo<br>2	Gran parte del tiempo<br>3	Parte del tiempo<br>4	En algún momento<br>5	En ningún momento<br>6
9. Durante las últimas 4 semanas, ¿con qué frecuencia:						
a. Se sintió muy animado?	☐	☐	☐	☐	☐	☐
b. Se sintió agotado?	☐	☐	☐	☐	☐	☐
c. Se sintió cansado?	☐	☐	☐	☐	☐	☐
d. Tuvo energía suficiente para hacer lo que quería hacer?	☐	☐	☐	☐	☐	☐
e. Se sintió abrumado por sus problemas de salud?	☐	☐	☐	☐	☐	☐
f. Se sintió desanimado por sus problemas de salud?	☐	☐	☐	☐	☐	☐
g. Sintió desesperación por sus problemas de salud?	☐	☐	☐	☐	☐	☐
h. Sintió temor debido a su estado de salud?	☐	☐	☐	☐	☐	☐

Encuesta de Salud MOS-VIH (continuación)

	Todo el tiempo<br>1	La mayor parte del tiempo<br>2	Gran parte del tiempo<br>3	Parte del tiempo<br>4	En algún momento<br>5	En ningún momento<br>6
10. Durante las últimas 4 semanas, ¿qué parte del tiempo:						
a. Tuvo dificultades para razonar y resolver problemas, como hacer planes, tomar decisiones, aprender cosas nuevas?	☐	☐	☐	☐	☐	☐
b. Olvidó cosas ocurridas recientemente, como dónde había puesto cosas y las citas que tenía?	☐	☐	☐	☐	☐	☐
c. Tuvo dificultades para mantener la atención centrada en alguna actividad durante un período prolongado?	☐	☐	☐	☐	☐	☐
d. Tuvo dificultades para realizar actividades que exigían concentración y reflexión?	☐	☐	☐	☐	☐	☐

11. Por favor, marque la casilla que mejor describa si cada una de las siguientes afirmaciones es cierta o falsa para usted

(Marque una casilla en cada línea)

	Totalmente cierta<br>1	Esencialment e cierta<br>2	No estoy seguro<br>3	Esencialment e falsa<br>4	Totalmente falsa<br>5
a. Estoy algo enfermo	☐	☐	☐	☐	☐
b. Estoy tan sano como cualquier persona que conozco	☐	☐	☐	☐	☐
c. Mi salud es excelente	☐	☐	☐	☐	☐
d. Últimamente me he sentido mal	☐	☐	☐	☐	☐

Encuesta de Salud MOS-VIH (continuación)

12. ¿Cómo ha sido su calidad de vida durante las últimas 4 semanas? Es decir, ¿cómo le han ido las cosas? (Marque sólo una respuesta)

Muy buena; difícilmente podría ser mejor	1	☐
Bastante buena	2	☐
Buena y mala a partes iguales	3	☐
Bastante mala	4	☐
Muy mala, difícilmente podría ser peor	5	☐

13. En comparación con hace 4 semanas ¿cómo calificaría su salud física y su estado emocional actuales? (Marque sólo una respuesta)

Mucho mejor	1	☐
Algo mejor	2	☐
Aproximadamente igual	3	☐
Algo peor	4	☐
Mucho peor	5	☐

MUCHAS GRACIAS POR SU COLABORACIÓN

Cuestionario de Salud EQ-5D

Versión Española

(Spanish version)

Marque con una cruz la respuesta de cada apartado que mejor describa su estado de salud el día de HOY.

Movilidad

No tengo problemas para caminar ❑

Tengo algunos problemas para caminar ❑

Tengo que estar en la cama ❑

Cuidado Personal

No tengo problemas con el cuidado personal ❑

Tengo algunos problemas para lavarme o vestirme ❑

Soy incapaz de lavarme o vestirme ❑

Actividades Cotidianas *(ej, trabajar, estudiar, hacer las tareas domésticas, actividades familiares o actividades durante el tiempo libre)*

No tengo problemas para realizar mis actividades cotidianas ❑

Tengo algunos problemas para realizar mis actividades cotidianas ❑

Soy incapaz de realizar mis actividades cotidianas ❑

Dolor/Malestar

No tengo dolor ni malestar ❑

Tengo moderado dolor o malestar ❑

Tengo mucho dolor o malestar ❑

Ansiedad/Depresión

No estoy ansioso ni deprimido ❑

Estoy moderadamente ansioso o deprimido ❑

Estoy muy ansioso o deprimido ❑

El mejor estado
de salud
imaginable

Para ayudar a la gente a describir lo bueno o malo que es su estado de salud hemos dibujado una escala parecida a un termómetro en el cual se marca con un 100 el mejor estado de salud que pueda imaginarse y con un 0 el peor estado de salud que pueda imaginarse.

Nos gustaría que nos indicara en esta escala, en su opinión, lo bueno o malo que es su estado de salud en el día de HOY. Por favor, dibuje una línea desde el casillero donde dice "Su estado de salud hoy" hasta el punto del termómetro que en su opinión indique lo bueno o malo que es su estado de salud en el día de HOY.

**Su estado
de salud
hoy**

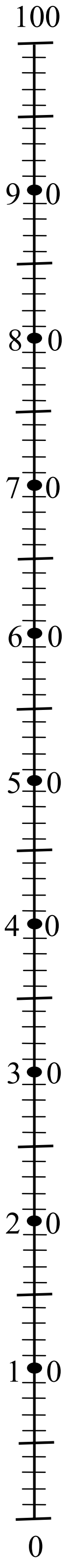

El peor estado
de salud
imaginable

Cuestionario CESTA: SATISFACCIÓN CON TARV

Por favor conteste a todas las preguntas marcando con una cruz la respuesta que mejor refleje su opinión, respecto al tratamiento antirretroviral que está recibiendo actualmente.

1. Respecto al tratamiento antirretroviral que está tomando actualmente: ¿cuál ha sido su grado de satisfacción sobre su estado
general de salud con el tratamiento que recibe?

	Muy Satisfecho
	Bastante Satisfecho
	Regular
	Bastante Insatisfecho
	Muy insatisfecho

2. Respecto al tratamiento antirretroviral que está tomando actualmente: ¿cuál ha sido su grado de satisfacción con el control de la
enfermedad producido por el tratamiento que recibe?

	Muy Satisfecho
	Bastante Satisfecho
	Regular
	Bastante Insatisfecho
	Muy insatisfecho

3. Respecto al tratamiento antirretroviral que está tomando actualmente: ¿cuál ha sido su grado de satisfacción con la ausencia de
efectos secundarios o molestias asociadas al tratamiento?

	Muy Satisfecho
	Bastante Satisfecho
	Regular
	Bastante Insatisfecho
	Muy insatisfecho

4. Respecto al tratamiento antirretroviral que está tomando actualmente: ¿cuál ha sido su grado de satisfacción con la cantidad de
pastillas que toma diariamente?

	Muy Satisfecho
	Bastante Satisfecho
	Regular
	Bastante Insatisfecho
	Muy insatisfecho

5. Respecto al tratamiento antirretroviral que está tomando actualmente: ¿cuál ha sido su grado de satisfacción con la cantidad de
veces que debe tomar el tratamiento al día?

	Muy Satisfecho
	Bastante Satisfecho
	Regular
	Bastante Insatisfecho
	Muy insatisfecho

6. Respecto al tratamiento antirretroviral que está tomando actualmente: ¿cuál ha sido su grado de satisfacción con los cambios
o restricciones en su alimentación producidas por la toma del tratamiento?

	Muy Satisfecho
	Bastante Satisfecho
	Regular
	Bastante Insatisfecho
	Muy insatisfecho

7. En general, ¿está usted satisfecho con el tratamiento antirretroviral que recibe actualmente?

	Muy Satisfecho
	Bastante Satisfecho
	Regular
	Bastante Insatisfecho
	Muy insatisfecho

8. ¿Qué importancia tienen para usted los siguientes aspectos para que esté satisfecho con un determinado tratamiento?
Marque con una cruz la casilla correspondiente para cada uno de los aspectos.

	Nada importante 0	Un poco importante 1	Regular 2	Bastante Importante 3	Muy importante 4
Control de la enfermedad					
Efectos secundarios					
Frecuencia de las tomas por día (1, 2 o más tomas al día)					
Número de pastillas que toma					
Cambio o restricción en la alimentación					

Referencias:

Condes E, et al. Validación del cuestionario de satisfacción con el tratamiento antirretroviral: cuestionario CESTA Enferm Infecc Microbiol Clin 2005;23(10):586-92

Vigilancia del cumplimiento del tratamiento

Cuestionario Estructurado GEEMA

¿Se olvida alguna vez de tomar los medicamentos?	SI □	NO □
¿Toma los fármacos a la hora indicada?	SI □	NO □
Si alguna vez se siente mal, ¿Deja de tomar la medicación?	SI □	NO □

Durante la última semana cuantas veces ha dejado de tomar la medicación

0 □ 1-2 □ 3-5 □ 6-10 □ Más de 10 □

¿Ha tenido algún olvido durante el fin de semana?	SI □	NO □
¿Cuántos días completos no ha tomado la medicación desde la última visita?	______ días	

¿Por qué no ha tomado la medicación?

- □ Olvido
- □ Efecto adverso
- □ Dificultades de horario
- □ Vacaciones o salidas de fin de semana
- □ Instrucciones confusas del médico
- □ Ninguna
- □ Otras ___________________________

Knobel H, Alonso J et al. Validation of a simplified medication adherence questionnaire in a large cohort of HIV-infected patients: the GEEMA Study. *AIDS 2002; 16: 605-613.]*

La mejor adherencia imaginable

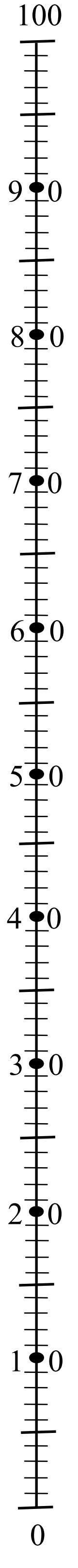

La peor adherencia imaginable

La adherencia a su tratamiento este último mes
