# Supplementary material for: High quality of life, treatment tolerability, safety and efficacy in HIV patients switching from triple therapy to lopinavir/ritonavir monotherapy: A randomized clinical trial
Source: PLoS One. 2018 Apr 12;13(4):e0195068. doi: 10.1371/journal.pone.0195068 (PMC5896909; doi:10.1371/journal.pone.0195068)
Supplement: S3 File — (PDF) [file pone.0195068.s006.pdf]

CLINICAL TRIAL PROTOCOL

A randomized, open label clinical trial to compare the quality of life of HIV+ patients who start monotherapy with LPV/r tablets versus triple therapy containing a boosted PI. QoLKAMON Study.

Sponsor: SAEI

Protocol code: SAI-CDV-2009-01

EudraCT code: 2009-014430-25

Final version: July 24th 2009

1. SYNOPSIS

1.	Sponsor identification:	SAEI<br>Sociedad Andaluza de Enfermedades Infecciosas<br>(Andalusian Society of Infectious Diseases)
2.	Title of clinical trial:	A randomized, open label clinical trial to compare the quality of life of HIV+ patients who start monotherapy with LPV/r tablets versus triple therapy containing a boosted PI.
3.	Protocol code:	SAI-CDV-2009-01
4.	EudraCT code:	2009-014430-25
5.	Name and qualification of monitors:	Effice spi S.L.<br>c/ Capitán Haya, 51 4º5<br>28020 Madrid
6.	Phase of clinical trial:	Phase IV
7.	Objectives	Primary objective: To compare the quality of life of patients who start monotherapy with lopinavir/ritonavir® (LPV/r) tablets versus patients on triple therapy containing any boosted protease inhibitor (PI).<br>Secondary objectives:<br>1. Assessment of virological efficacy of monotherapy based on LPV/r tablets versus triple therapy containing any PI.<br>2. Assessment of changes in immune response in HIV+ patients who start monotherapy with LPV/r tablets versus triple therapy containing any PI.<br>3. Assessment of treatment satisfaction of HIV+ patients who start monotherapy with LPV/r tablets versus triple therapy containing any PI.<br>4. Assessment of treatment adherence of HIV+ patients who start monotherapy with LPV/r tablets versus triple therapy

containing any PI.

5. Assessment of regimen tolerability and safety in HIV+ patients who start monotherapy with LPV/r tablets versus triple therapy containing any PI.

8. Design:	A Phase IV national, multicenter, controlled, randomized, open label, parallel-group, comparative clinical trial
9. Randomization process:	Randomization will be centrally performed.
10. Treatment groups:	Treatment:<br>Patients will be randomized in a 1:2 ratio to two groups, A and B respectively.<br><br>**Control group (A)**: Patients will continue on triple antiretroviral therapy based on any PI boosted with ritonavir.<br><br>**Experimental group (B)**: Triple antiretroviral therapy based on any PI boosted with ritonavir will be switched to LPV/r monotherapy.<br><br>Central randomization will be performed.
11. Disease under study:	HIV infection
12. Primary endpoint:	Quality of life, as measured by the MOS-HIV and EQ-5D questionnaires, of patients who start monotherapy with lopinavir/ritonavir (LPV/r) tablets versus patients on triple therapy containing any boosted protease inhibitor (PI).
13. Number of subjects:	390 (260 in experimental group B and 130 in control group A)

14. Selection criteria:

Inclusion criteria:

- Patients infected with HIV-1, as documented by a positive HIV-1 antibody test and/or a documented positive PCR for HIV-1 RNA.
- Patients on antiretroviral triple therapy containing any boosted PI.
- Patients with an undetectable viral load, defined as < 50 copies/mL of HIV RNA in the past 6 months.
- Males or females aged ≥ 18 years.
- For women of childbearing age, a negative urine pregnancy test at the screening visit.
- Patients who have given written informed consent before undergoing any study-specific screening procedure.

Exclusion criteria:

- Patients with documented record, in the resistance accumulated genotyping, of mutations that confers loss of susceptibility to LPV/r. Or, in the absence of genotyping, evidence of having failed to therapy with protease inhibitors (PI)
- Patients with a CD4 cell nadir <100 cel/μL.
- Patients who cannot be treated with LPV/r® for any reason.
- History of psychiatric disorders such as depressive syndrome, schizophrenia, or psychotic disease.
- Known history of drug addiction or chronic alcohol consumption that contraindicates participation in the study in the investigator’s criterion.
- Pregnant or nursing women or women of childbearing age not using an adequate contraceptive method according in the investigator’s criterion.
- Current active opportunistic infection or documented

infection within 4 weeks of screening.

- Patients in whom modification or withdrawal of any component of the current antiretroviral triple therapy is planned due to serious toxicities related to any component of such therapy.
- Patients in whom investigators consider that antiretroviral medication should be changed for any reason in the next 6 months.
- Renal disease with creatinine clearance < 60 mL/min.
- Concomitant use of drugs contraindicated with LPV/r® such as rifampin, dihydroergotamine, ergotamine, methylergonovine, cisapride, St. John's wort, lovastatin, simvastatin, pimozide, midazolam, and triazolam.
- Concomitant use of nephrotoxic or immunosuppressive agents.
- Patient on current treatment with systemic corticosteroids, interleukine-2, or chemotherapy.
- Patients treated with other investigational agents.
- Patients with acute hepatitis.
- Any disease that, in the investigator's judgment, contraindicates patient participation in the study.

15. Treatment duration: 24 weeks.

16. Timetable and expected completion date: The approximate recruitment time will be 6 weeks. Treatment duration will be 24 weeks.
Start of recruitment: November 2009.
End of recruitment: June 2010.
End of follow-up of last patient: December 2010.
Final report: March 2011.

2. LIST OF ABBREVIATIONS

3TC	Emtricitabine
AE	Adverse events
ART	Antiretroviral therapy
AZT	Zidovudine
AZT/3TC	Combivir
CREC	Clinical Research Ethics Committee
CRF	Case Report Form
CRO	Contract Research Organization
EQ-5D	EuroQol 5-D
HAART	Highly active antiretroviral therapy
HIV-1	Acquired immunodeficiency virus
LPV/r	Lopinavir/ritonavir.
MOS_HIV	Medical Outcome Study in HIV
NNRTIs	Non-nucleoside analogue reverse transcriptase inhibitors
NRTIs	Nucleoside analogue reverse transcriptase inhibitors
PCR	Polymerase chain reaction
PI	Protease Inhibitors
QoL	Quality of life
RTV	Ritonavir
SAE	Serious adverse events
SAMPMD	Spanish Agency for Medicinal Products and Medical Devices
TDF	Tenofovir
TDF+3TC	Truvada
VAS	Visual analog scale

TABLE OF CONTENTS

1. SYNOPSIS 2
2. LIST OF ABBREVIATIONS 6
TABLE OF CONTENTS 7
3. GENERAL INFORMATION 9
4. RATIONALE AND OBJECTIVES 10
4.1 RATIONALE 10
4.2 STUDY OBJECTIVES: 12
5. CLINICAL TRIAL TYPE AND DESIGN 13
5.1 DESIGN 13
5.2 PATIENT ENROLLMENT 13
5.3 PATIENT RANDOMIZATION 13
6. SUBJECT SELECTION 14
6.1 PLANNED NUMBER OF SUBJECTS 14
6.2 SCREENING CRITERIA 14
6.3 NON-COMPLIANCE WITH SCREENING CRITERIA 15
6.4 TREATMENT DISCONTINUATION. STUDY WITHDRAWAL. 16
6.5 ESTIMATED DURATION OF THE RECRUITMENT PERIOD 16
7. DESCRIPTION OF TREATMENT 17
7.1 DRUG ADMINISTRATION SCHEME 17
7.2 Patient withdrawal criteria 17
7.2 PRIOR AND CONCOMITANT TREATMENT 18
8. STUDY CONDUCT AND EFFICACY ASSESSMENT 20
8.1. STUDY ENDPOINTS AND MEASUREMENT INSTRUMENTS 20
8.1.1 Primary endpoint: 20
8.1.2 Secondary endpoints: 20
8.2. STUDY CONDUCT 21
8.2.1 Study timetable and procedures. 21
8.2.2 Subject recruitment and treatment assignment 23
8.2.3 Treatment assessments 23
8.2.4 Post-treatment assessments 26
8.2.5 Early study discontinuation 26
8.2.6 Criteria for treatment discontinuation or modification 27
9. ADVERSE EVENTS AND MANAGEMENT OF TOXICITY 28
9.1. ADVERSE EVENTS: 28
9.2. MANAGEMENT OF TOXICITY: 31
10. ETHICAL ISSUES 32
10.1 GENERAL CONSIDERATIONS 32
10.2 OBTAINING INFORMED CONSENT 32
11. PRACTICAL CONSIDERATIONS 32
11.1 RESPONSIBILITIES OF STUDY PARTICIPANTS 32

11.2 DATA MANAGEMENT AND FILE 33
11.3 PUBLICATION CONDITIONS 33
11.4 PROTOCOL AMENDMENT PROCEDURE 33
11.5 CLINICAL RESEARCH ETHICS COMMITTEE (CREC) 34
12. STATISTICAL ANALYSIS 35
12.1. Population for analysis 36
12.2. Primary endpoint 36
12.3. Secondary endpoints 36
12.4. Statistical methods. 37
REFERENCES 39
PROTOCOL APPENDICES 43
APPENDIX 1 Protocol signature document for the principal investigator 44
APPENDIX 2 WORLD MEDICAL ASSOCIATION DECLARATION OF HELSINKI 46
APPENDIX 3 PATIENT INFORMATION SHEET AND INFORMED CONSENT 52
APPENDIX 4 Serious adverse event reporting form 65
APPENDIX 5 ACTG CLASSIFICATION OF ADVERSE EVENT SEVERITY IN ADULTS 66
EQ-5D Health Questionnaire 78

3. GENERAL INFORMATION

A. Trial identification

1.- Protocol code:	SAI-CDV-2009-01
2.- EudraCT code:	2009-014430-25
3.- Title:	A randomized, open label clinical trial to compare the quality of life of HIV+ patients who start monotherapy with LPV/r tablets versus triple therapy containing a boosted PI.

B. Type of clinical trial:	A Phase IV multicenter, national, randomized, open label, prospective study.
C. Description of test products:	Kaletra®: (Lopinavir/ritonavir).<br>Film-coated tablets containing 200 mg of lopinavir co-formulated with 50 mg of ritonavir.<br>Administration route: Oral.<br>Therapeutic group: Protease Inhibitor (J05AE06)
D. Sponsor data:	SAEI<br>Soc.Andaluza de Enfermedades Infecciosas<br>(Andalusian Society of Infectious Diseases)<br>C/ Reposo 6, bajo 6 (41002).Sevilla<br>Teléfono: 954389553<br>e-mail:secretariatecnica@saei.e.telefonica.net
E. Monitor identification:	Effice spi S.L.<br>c/ Capitán Haya, 51 4º5<br>28020 Madrid

4. RATIONALE AND OBJECTIVES

4.1 RATIONALE

Current antiretroviral treatment for HIV-infected adults is based on the combination of two nucleoside analogue reverse transcriptase inhibitors (NRTIs) combined with a non-nucleoside analogue reverse transcriptase inhibitor (NNRTI) or a protease inhibitor (PI) boosted with a low dose of ritonavir (RTV) **[1].**

Despite the great success of highly active antiretroviral therapy (HAART), triple therapy regimens are associated to long-term toxicity, high costs, and a complex administration that may have direct consequences for HIV-infected patients because of a decreased treatment adherence, treatment dissatisfaction, the negative impact caused on quality of life (QoL), and above all because it could affect treatment efficacy **[1-5].**

Triple therapy based on NRTIs and a PI has been associated to a number of significant metabolic adverse effects such as body fat redistribution (lipoatrophy of the face and limbs, central obesity) and hyperlipidemia (hypercholesterolemia and hypertriglyceridemia). Although the great majority of metabolic adverse effects were initially attributed to use of PIs, the hypothesis that adverse effects such as lipoatrophy are more related to antiretroviral therapies containing NRTIs than to PIs has gained weight over the years. In the Prometheus study **[6]**, a greater incidence of lipoatrophy was seen in patients treated with nucleosides as compared to those receiving two PIs (ritonavir and saquinavir combined).

Another significant adverse effect of HAART is a form of lactic acidosis causing a high mortality **[7].** It is currently accepted that severe lactic acidosis in patients receiving two nucleosides and a PI is due to the mitochondrial toxicity induced by nucleosides, particularly d4T.

Use of a PI boosted with RTV may also complicate the treatment regimen for some patients. This PI-based regimen not only requires additional administration of RTV capsules, but also special storage conditions due to the recommendation of maintaining the capsules refrigerated **[8].** Kaletra® is a LPV/r co-formulation with a high antiviral potency which has been shown to have long-term efficacy and safety in combination with other antiretroviral agents **[9].**

LPV/r is characterized by a very high pharmacological and genetic barrier. Trough plasma levels of LPV/r are more than 70-fold higher than the EC_{50} for wild-type HIV. Occurrence of resistance to LPV/r requires accumulation of mutations in the protease gene. The EC_{50} for wild-type HIV starts to show a clinically significant increase from five mutations **[10-11]**. In

study 863, none of the patients treated with LPV/r with a viremia rebound to more than 400 copies/mL developed mutations in the protease gene **[12].**

In addition, the new formulation of LPV/r as tablets decreases pill overload and also eliminates the requirement of refrigeration. Administration of medication with food is not required either **[9]**.

Together, these advantages promote simplification of antiretroviral therapy and a potential improvement in adherence. In recent years, data have been reported on use of LPV/r monotherapy as starting treatment **[13]**, as maintenance after initial therapy **[14]**, and as maintenance following HAART with undetectability sustained for at least 6 months **[15-17].**

The third approach is the one that has been shown to date to be safest for use of LPV/r as monotherapy, based on reported data from the OK04 study (a randomized, comparative study on 200 patients) at 48 weeks **[15]** and at 96 weeks **[18]**.

In another open label study (MONARK) in which naïve patients were randomized to receive monotherapy based on LPV/r® or standard antiretroviral triple therapy using LPV/r (Kaletra®)+ AZT/3TC (Combivir®), virological efficacy was similar in both groups at 48 weeks of treatment even in patients in the monotherapy group who had episodes of lower viremia levels **[5]**. In addition, the mean total symptoms reported and the number of symptoms causing discomfort were significantly higher in the triple therapy group as compared to the monotherapy group (relative risk 1.3 and 1.4; P= 0.001 and P= 0.0003 respectively), and the proportion of patients with a positive perception of their overall quality of life increased in the monotherapy group from 32% at baseline to 68% at 48 weeks of study (P<0.0001), and in the triple therapy group from 46% to 59% (P= 0.38) **[5]**.

Finally, in a randomized, open label, 48-week study in HIV+ patients with an inhibited viral replication switched from antiretroviral triple therapy based on LPV/r to monotherapy based on LPV/r, the proportion of patients who maintained an undetectable viral load in the monotherapy group was not statistically different from that of patients from the group continuing on triple therapy (81% vs 95%, P=0.34) **[5]**.

<u>Rationale for the study:</u>

Over the past years, the simplification approach of LPV/r as monotherapy has been shown to be not inferior to triple therapy in virological and immunological efficacy.

In Spain, use of simplification to LPV/r monotherapy has been included since January 2008 in treatment guidelines as a therapeutic option for patients with no history of prior PI failure, an undetectable viral load for at least 6 months, and signs and/or symptoms of toxicity from NRTIs **[19]**.

The purpose of this randomized, open label, 24-week study is to compare the quality of life of patients who start monotherapy with co-formulated LPV/r tablets as compared to patients with HAART based on any boosted PI.

4.2 STUDY OBJECTIVES:

Secondary objectives:

4.2.1. Primary objective:

- To compare the quality of life of patients who start simplification to monotherapy with lopinavir/ritonavir (LPV/r) tablets versus patients who continue on triple therapy containing any boosted protease inhibitor (PI).

4.2.2. Secondary objectives:

- Assessment of treatment satisfaction of HIV+ patients who start simplification to monotherapy with LPV/r tablets versus triple therapy containing any PI.
- Assessment of treatment adherence of HIV+ patients who start simplification to monotherapy with LPV/r tablets versus triple therapy containing any PI.
- Assessment of regimen tolerability and safety in HIV+ patients who start simplification to monotherapy with LPV/r tablets versus triple therapy containing any PI.
- Assessment of virological efficacy of simplification to monotherapy based on LPV/r tablets versus triple therapy containing any PI.
- Assessment of changes in immune response in HIV+ patients who start simplification to monotherapy with LPV/r tablets versus triple therapy containing any PI.

5. CLINICAL TRIAL TYPE AND DESIGN

5.1 DESIGN

A Phase IV multicenter, national, randomized, open label, prospective study in HIV+ patients on antiretroviral triple therapy containing any boosted protease inhibitor (PI), as compared to patients who start simplification to monotherapy with LPV/r tablets.

5.2 PATIENT ENROLLMENT

When a patient is considered to be eligible for the study, he/she will be asked to participate in it, for which the patient information sheet (Appendix 3) and any additional information that may be required will be provided. Once informed consent is obtained from the patient, the patient enrollment form, will be faxed to the Study Data Center in order to obtain patient randomization and a patient screening number:
[For more information, please see section 5.3 “patient randomization”.

The following data will be recorded in the enrollment form:

- Rank number of selection at the patient center.
- Investigating center
- Investigator

5.3 PATIENT RANDOMIZATION

Randomization will be centralized and will be performed from the study data center upon data reception. Central randomization will be performed.

Before patients begin the study, patients will be randomized according to a randomization list. The list will be created by generating pseudo-random numbers of Wichmann and Hill (1982) and modified by McLeod (1985). The size of the blocks will be 4 to try to balance the treatment arms of the total sample and within each center. No stratification was planned by any other factor

The randomization list will be centralized and hidden from the investigators. Investigators will access the allocated treatment by telephone at the time of randomization with the sponsor or who is assigned on its behalf. CRD corresponding page will be sent by fax prior to the phone call. CRD corresponding page confirms that the patient meets all inclusion criteria and none of exclusion in order to know the branch of study is given and the number of patients used for

the same. This information is written back to the researcher in the same format for receiving your application, including the number assigned to the patient and the branch of study.

Patients will be randomized in a 1:2 ratio to one of the following two options:

Control group (A): Patients will continue on antiretroviral triple therapy based on any PI boosted with ritonavir.

Experimental group (B): Patients will be switched from antiretroviral triple therapy based on any PI boosted with ritonavir to LPV/r monotherapy (co-formulated tablets).

6. SUBJECT SELECTION

6.1 PLANNED NUMBER OF SUBJECTS

A total of **390 patients** will be enrolled into this study (130 patients in control group A and 260 patients in experimental group B).

6.2 SCREENING CRITERIA

Before any protocol-specific procedures are started, written informed consent of the patient must be obtained and documented.

Inclusion criteria:

- Patients infected with HIV-1, as documented by a positive HIV-1 antibody test and/or a documented positive PCR for HIV-1 RNA.
- Patients on antiretroviral triple therapy containing any boosted PI.
- Patients with an undetectable viral load, defined as < 50 copies/mL in the past 6 months.
- Males or females aged ≥ 18 years.
- For women of childbearing age, a negative urine pregnancy test at the screening visit.
- Patients who have given written informed consent before undergoing any study-specific screening procedure.

Exclusion criteria:

- Patients with documented record, in the resistance accumulated genotyping, of mutations that confers loss of susceptibility to LPV/r. Or, in the absence of genotyping, evidence of having failed to therapy with protease inhibitors (PI)

- Patients with a CD4 cell nadir <100 cel/μL.
- Patients who cannot be treated with LPV/r for any reason.
- History of psychiatric disorders such as depressive syndrome, schizophrenia, or psychotic disease. Known history of drug addiction or chronic alcohol consumption that contraindicates participation in the study in the investigator's criterion.
- Pregnant or nursing women or women of childbearing age not using an adequate contraceptive method according in the investigator's criterion.
- Current active opportunistic infection or documented infection within 4 weeks of screening.
- Patients in whom modification or withdrawal of any component of the current antiretroviral triple therapy is planned due to serious toxicities related to any component of such therapy. Patients in whom investigators consider that antiretroviral medication should be changed for any reason in the next 6 months.
- Renal disease with creatinine clearance < 60 mL/min.
- Concomitant use of drugs contraindicated with LPV/r such as rifampin, dihydroergotamine, ergotamine, methylergonovine, cisapride, St. John's wort, lovastatin, simvastatin, pimozide, midazolam, and triazolam.
- Concomitant use of nephrotoxic or immunosuppressive agents.
- Patient on current treatment with systemic corticosteroids, interleukine-2, or chemotherapy.
- Patients treated with other investigational agents.
- Patients with acute hepatitis.
- Any disease that, in the investigator's judgment, contraindicates patient participation in the study.

6.3 NON-COMPLIANCE WITH SCREENING CRITERIA

All patients should meet all inclusion criteria and none of the exclusion criteria. To prevent enrollment of non-eligible patients, any doubt should be consulted with the sponsor. If a patient does not meet the eligibility criteria and is inadvertently enrolled into the study, this should be reported to the study sponsor so that patient continuation in the study may be assessed, evaluating the risks and benefits for the patient and ensuring maximum safety.

6.4 TREATMENT DISCONTINUATION. STUDY WITHDRAWAL.

Any patient will be withdrawn from the study in the following circumstances:

- At the request of the patient.
- Serious adverse event or unacceptable toxicity.
- Pregnancy.
- When the investigator thinks that a change in treatment not planned in the protocol is required in the best interest of the patient.

The date and reasons for any early discontinuation will be recorded in the Case Report Form (CRF) and considered in the final assessment.

6.5 ESTIMATED DURATION OF THE RECRUITMENT PERIOD

Recruitment will start once the relevant approvals have been obtained. The planned duration of the recruitment period is 6 months. The planned total study duration is 12 months.

7. DESCRIPTION OF TREATMENT

7.1 DRUG ADMINISTRATION SCHEME

Patients will be randomized to one of the following treatment groups:

Control group (A): Patients will continue on antiretroviral triple therapy based on any PI boosted with ritonavir.
Medication will be administered in accordance to the relevant prescribing information.

Experimental group (B): Switch to LPV/r monotherapy.
Kaletra® alone, two tablets twice daily.

7.2 Patient withdrawal criteria

Therapeutic failure

Patients who meet therapeutic failure criteria will be withdrawn from the study. These criteria are defined as follows:

1. In patients receiving triple therapy randomly assigned at baseline, documented virological failure, i.e. the presence of a viral load greater than 500 copies/mL on 2 occasions 15 days apart, is considered as therapeutic failure.
2. Patients assigned to maintenance with LPV/r alone who reach the virological failure criterion will be considered as therapeutic failures if protease gene mutations exist, so that the patient cannot be reinduced with nucleosides and would discontinue the study because of the need of a therapy not based on LPV/r.
3. However, patients assigned to maintenance with LPV/r alone can be reinduced with the same nucleosides as withdrawn if no documented resistance to LPV/r exists. Thus, the following specific situation (a+b+c) will NOT be considered as therapeutic failure and will not lead to study discontinuation:
    a) A patient in the maintenance arm with LPV/r alone has a viral load greater than 500 copies/mL on 2 occasions 15 days apart.
    b) The genotypic resistance test shows no resistance to LPV/r.
    c) The patient is reinduced with the same nucleosides as received before study entry and completes the follow-up period with an undetectable VL.

If this situation has occurred, and once patients are reinduced, they will be considered as therapeutic failures when the following conditions are met:

- 4 weeks after reinduction, viral load has not decreased by at least one decimal logarithmic unit
- and/or does not become undetectable again (<50 copies/mL) at 16 weeks of reinduction
- or if once on triple therapy they experience virological failure again.

In patients in whom a viral load ranging from 50-500 copies/mL is obtained, the above procedure will be followed.

After this viral load has been confirmed three times (2 weeks, 2 weeks, and 4 weeks), the following will be done:

- Patients in the monotherapy group and with no lopinavir resistance will be reinduced with the same nucleosides and the procedure in the previous paragraphs will be followed.
- Patients on triple therapy or in the monotherapy group but with lopinavir resistance: these will be considered as therapeutic failures, and after designing rescue treatment a follow-up visit will be scheduled 4 weeks after the end of study visit.

Annex 1 to the protocol provides a detailed scheme of management of patients with virological failure.

Intolerance

A serious adverse effect as defined in section 7.7 (Scheme modification criteria for toxicity).

7.2 PRIOR AND CONCOMITANT TREATMENT

Patients will continue to receive their concomitant medications as prescribed. Any concomitant medication should be recorded in the Case Report Form (CRF). In addition, any diagnostic, therapeutic, or surgical procedure performed during the study period should be recorded, including the date, indication, description of procedure(s) and any clinical finding.

In order to guarantee patient safety, the following medications not recommended concomitantly with Kaletra will be excluded:

Kaletra®. Contains lopinavir/ritonavir, two inhibitors of the P450 CYP3A isoform, and should therefore not be administered together with drugs whose clearance largely depends on

CYP3A and for which an increase in plasma levels is associated with serious and/or life-threatening effects. Such drugs include astemizole, terfenadine, oral midazolam, triazolam, cisapride, pimozide, amiodarone, ergot alkaloids (e.g. ergotamine, dihydroergotamine, ergonovine, and methylergonovine), and vardenafil.

Herbal drugs containing St. John's wort (*Hypericum perforatum*) should not be used while lopinavir and ritonavir are being taken because of the risk of reducing plasma levels and clinical effects of lopinavir and ritonavir. Rifampin should not be used in combination with Kaletra® because it may markedly decrease lopinavir concentrations and thus significantly decrease its therapeutic effect.

If the patients need to start treatment with any of the excluded medications, the study coordinator should be consulted before it is started.

7.3 DESCRIPTION OF STUDY DRUGS

Description and handling of Kaletra®:

Formulation:

Each film-coated tablet of Kaletra® contains 200 mg of lopinavir and 50 mg of ritonavir as booster. Kaletra® 200/50 mg tablets are yellow coated tablets having engraved the Abbott logo and "KA". Excipients: each tablet contains copovidone, sorbitan laureate, colloidal anhydrous silica, and sodium stearyl fumarate. See the product label for additional information.

Packaging and labeling:

PVC/fluoropolymer blisters. Each carton contains 5 aluminium blisters with 8 film-coated tablets each (40 tablets). Each package contains 3 cartons (120 tablets).

Storage and handling:

This product requires no special storage conditions.

7.4 SUPPLY OF STUDY DRUG

During the study conduct, Kaletra will be provided (at no cost for the hospital) to any patient who needs to start treatment with this medicinal product because of participation in the study. The medication will be identified as "trial medication", and commercial labeling will therefore not be used.

Annex 2 provides a schematic application process for medication, and the re-labeling of medication for clinical use only.

8. STUDY CONDUCT AND EFFICACY ASSESSMENT

8.1. STUDY ENDPOINTS AND MEASUREMENT INSTRUMENTS

8.1.1 Primary endpoint:

- Changes in quality of life at 24 weeks (from the baseline visit) based on the parameters of the Medical Outcomes Study HIV Health Survey (MOS-HIV) and the EuroQol 5D visual analog scale (EQ-5D)**[20]**.

8.1.2 Secondary endpoints:

- Assessment of treatment satisfaction of HIV+ patients who start monotherapy with LPV/r tablets versus triple therapy containing any PI using the CESTA questionnaire (Spanish Questionnaire of Satisfaction with Antiretroviral Treatment) **[21]** from the baseline visit to study week 24.
- Assessment of treatment adherence by HIV+ patients who start monotherapy with LPV/r tablets versus triple therapy containing any PI using the GEEMA questionnaire **[22]** and a visual analog scale (VAS) **[23]**.
- Assessment of regimen tolerability and safety in HIV+ patients who start monotherapy with LPV/r tablets versus triple therapy containing any PI.
- Proportion of patients with a viral load <50 copies/mL of HIV RNA at 24 weeks in HIV+ patients who start monotherapy with LPV/r tablets versus triple therapy containing any PI. A viral load >500 copies/mL of viral RNA (HIV-1) confirmed at study week 24 will be considered as virological failure.
- Changes in immune response in HIV+ patients who start monotherapy with LPV/r tablets versus triple therapy containing any PI, as measured through the change in CD4+ cell count from the baseline visit to study week 24

8.2. STUDY CONDUCT

8.2.1 **Study timetable and procedures.**

Procedures are shown in the following table and detailed below:

	Screening visit	Baseline visit	Week 4 visit	Week 12 visit	Week 24 visit	Early discontinuation visit
Randomization		X (24 h before)				
Informed consent	X	X				
Clinical history	X	X				
Complete physical examination(1)	X	X		X	X	X
Pregnancy test(1)	X	X				
HIV-1 RNA in plasma	X	X	X*	X	X	X
CD4 count	X	X	X*	X	X	X
Hepatology**		X		X	X	X
Chemistry(2)**		X		X	X	X
**MOS-HIV Questionnaire**		X			X	X
**EQ-5D Questionnaire**		X			X	X
CESTA Questionnaire		X	X*	X	X	X
GEEMA Questionnaire		X	X*	X	X	X
Adherence VAS		X	X*	X	X	x
Concomitant medication		X	X*	X	X	X
Adverse events		X	X*	X	X	X

(1) includes vital signs and weight measurement. Since adult patients will be enrolled, height will be measured at the baseline visit.

*Only for patients in the experimental arm.

**Record relevant data related to adverse events.

(2) Urine pregnancy test for women of childbearing age.

Any deviation from the protocol procedures should be reported to the sponsor and recorded in the Case Report Forms (CRFs).

All laboratory samples will be sent to local laboratories to be tested. The same local laboratory should be used throughout patient participation in the trial.

8.2.2 Subject recruitment and treatment assignment

The investigator is responsible for ensuring that each subject is eligible for the study before recruitment. Once a subject number has been assigned, it cannot be assigned to another subject.

8.2.3 Treatment assessments

Quality of life measurement:

Handling of questionnaires will be specifically detailed in Annex 2. As a general rule, each questionnaire should be provided to the patient based on the following premises:

- The following questionnaires ask patients about many aspects of their health state and health care. Questionnaires should be provided to patients before the physical examination, preferably in a quiet environment (e.g. the examination room or other room).
- It is important to be familiar with the content and format of questionnaires before they are given to the study participants. At the first visit, instruct the patient as follows:

"We would like you to answer some questions about how you feel and what you are able to do. Your answers will help us understand the effects of the medication you are taking. We would appreciate it if you would complete these questionnaires."

- You should then briefly explain the format of questions and how to complete them. The patient must complete the questionnaires before the clinical history and physical examination are made and the vital signs are measured.
- Questionnaires are very short and should take no more than 15-30 minutes to be completed. Before questionnaires are given to the patient, fill in the heading and DETACH THE PAGE for the investigator, which is adequately marked.

- Collect the completed questionnaires before the physical examination. Before continuing, review the questionnaires for potential omissions. If the participant has not answered any question, point this to him/her and have him/her complete the omissions.

<u>Other study procedures:</u>

In addition to quality of life measurements (section 8.2), that will be done using the corresponding questionnaires, the following procedures will be performed to cover the secondary objectives of the study.

Screening visit:

The eligibility of each subject will be established within 3 weeks of study randomization. This should be done at this visit, and the following should be documented:

- Written informed consent.
- Clinical history including a history of events related to the disease and drugs used in the previous 30 days.
- Physical examination (vital signs, height and weight).
- Urine pregnancy test (women of childbearing age).
- Complete blood count (red blood cell count, white blood cell count and differential, and platelet count).
- Fasting chemistry profile (alkaline phosphatase, ALT, AST, GGT, total bilirubin, creatinine, glucose, potassium, sodium, uric acid, and lipid profile (total cholesterol, HDL-C, LDL-C, VLDL-C, triglycerides).
- CD4 cell count
- HIV-1 RNA in plasma

Baseline visit:

If laboratory data for confirming screening criteria have been obtained within two months of study recruitment and are available, the screening visit may be the same as the baseline visit.

The study drugs should start to be taken within 24 hours of the baseline visit. The following assessments should be available at the baseline visit:

- Physical examination (vital signs, height and weight).
- Quality of life questionnaires (MOS-HIV and EQ-5D)
- A negative urine pregnancy test for women of childbearing age.

- Complete blood count (red blood cell count, white blood cell count and differential, and platelet count).
- Fasting chemistry profile (alkaline phosphatase, ALT, AST, GGT, total bilirubin, creatinine, glucose, potassium, sodium, uric acid, lipid profile (total cholesterol, HDL-C, LDL-C, VLDL-C, triglycerides).
- CD4 cell count
- HIV-1 RNA in plasma: This will be measured in real time at the site using the standard virological methods at each site. The same methods should be used throughout the study for each subject.
- CESTA questionnaire to assess satisfaction with antiretroviral treatment.
- GEEMA questionnaire to assess treatment adherence
- VAS to assess treatment adherence
- Recording of concomitant medication
- Adverse events

Once the baseline visit procedures and assessments are reviewed, subjects will be centrally randomized at the study data center after data are received.

Patients randomized to group B will return for HIV RNA monitoring at treatment week 4 (+/- 2 days)

The following assessments should be completed at the **week 12 and week 24 visits or at the study discontinuation visit**. Study visits should be scheduled to occur within ± 1 week of the date specified for weeks 12 and 24, calculating the dates from the baseline visit:

- Physical examination (vital signs, weight).
- Complete blood count (red blood cell count, white blood cell count and differential, and platelet count)
- Fasting chemistry profile (alkaline phosphatase, ALT, AST, GGT, total bilirubin, creatinine, glucose, potassium, sodium, uric acid, lipid profile (total cholesterol, HDL-C, LDL-C, VLDL-C, triglycerides)
- CD4 cell count
- HIV-1 RNA in plasma: If the patient shows at any time a detectable viral load (> 50 copies/mL), the test should be repeated within 2 weeks for confirmation. Annex 1 provides suggestions for management of these cases
- Concomitant medication
- Adverse events

The following assessments should be completed at the end of the **week 12 and week 24 visits or at the early study discontinuation visit**.

- Questionnaires for assessing treatment adherence (GEEMA and VAS)
- Questionnaire for assessing treatment satisfaction (CESTA)
- Concomitant medication
- Adverse events

The following assessments should be completed at the end of the **baseline and week 24 visits or at the early study discontinuation visit**.

- Quality of life questionnaires (MOS-HIV and EQ-5D)
- Questionnaires for assessing treatment adherence (GEEMA and VAS)
- Questionnaire for assessing treatment satisfaction (CESTA)
- Concomitant medication
- Adverse events

8.2.4 Post-treatment assessments

Only if it has been reported any adverse event that may be under way in the last visit or, if the patient had presented a viral load> 50 copies / mL at that visit, a telephone follow-up call will be made after 30 days of last visit study to monitor safety.

8.2.5 Early study discontinuation

In the event of early discontinuation, the subject should return to the site as soon as possible (preferably within 72 hours of study drug discontinuation) for an early study discontinuation visit. The following assessments should be completed at that visit:

- Physical examination (vital signs, weight).
- Complete blood count (red blood cell count, white blood cell count and differential, and platelet count).
- Fasting chemistry profile (alkaline phosphatase, ALT, AST, GGT, total bilirubin, creatinine, glucose, potassium, sodium, uric acid, lipid profile (total cholesterol, HDL-C, LDL-C, VLDL-C, triglycerides).
- CD4 cell count
- HIV-1 RNA in plasma: If the patient shows at any time a detectable viral load (> 50 copies/mL), the test should be repeated within 2 weeks for confirmation
- Quality of life questionnaires (MOS-HIV and EQ-5D)

- Questionnaires for assessing treatment adherence (GEEMA and VAS)
- Questionnaire for assessing treatment satisfaction (CESTA)
- Concomitant medications.
- Adverse events

8.2.6 Criteria for treatment discontinuation or modification

Study medication should be discontinued if the following occurs:

- Underlying disease that may significantly affect assessments of clinical status in the investigator’s judgment.
- Unacceptable toxicity (toxicity that compromises the ability to continue with the protocol-specific procedures).
- At the request of the subject.
- Pregnancy
- Therapeutic failure, defined as 2 VL measurements > 500 copies/mL at lest 2 weeks apart in the experimental group and/or genotyping with PI mutations that prevent continued treatment with LPV/r monotherapy.

9. ADVERSE EVENTS AND MANAGEMENT OF TOXICITY

9.1. ADVERSE EVENTS:

Definition of adverse events and categories of serious adverse events

Investigators will routinely monitor all subjects during the study for detecting SAEs. An AE is defined as any untoward medical occurrence in a patient during treatment but that does not necessarily have a causal relationship with treatment. An AE may therefore be any untoward and undesired sign (such as an abnormal laboratory finding), symptom, or disease temporally associated to use of a medicinal product, whether or not the event is causally related to use of the product.

This event may occur due to use of the drug as stipulated in the protocol or to accidental or intentional overdose, abuse, or discontinuation of the drug. Any worsening of a pre-existing condition or disease is considered as an AE.

All adverse events will be recorded in the Case Report Form (CRF).

Serious adverse event (SAE): definition and reporting procedure

A serious adverse event (SAE) is any event that is fatal or life-threatening, requires or prolongs patient hospitalization, causes persistent or significant incapacity or disability, is a congenital anomaly or birth defect, or is a clinically significant event.

Clinically significant events are those that may not be immediately life-threatening, but are clearly clinically significant. They may jeopardize the subject or require intervention to prevent any serious outcome. Overdose or abuse of a drug will usually be considered as serious.

**Death**	An event that results in patient death.
**Life-threatening**	An event that, in the investigator's judgment, could have caused the immediate death of the patient if no medical intervention had occurred. This does not include the events that could had been fatal had they been more severe.
**Hospitalization**	An event that requires hospital admission, regardless of hospitalization time. This does not include emergency room visits or the stay at an outpatient center.
**Prolongation of hospitalization**	An event that occurs while the study subject is hospitalized and prolongs hospital stay.

**Congenital anomaly**	An anomaly detected at of after delivery, or any anomaly causing fetal loss.
**Persistent or significant disability or incapacity**	An event that causes a change significantly impairing the daily activities of a patient. Disability does not include experiences that have relatively little medical significance, such as headache, vomiting, diarrhea, flue, or accidental trauma (e.g. an ankle sprain).
**Medically important event requiring medical or surgical intervention to prevent a serious outcome**	Medically important event that does not cause death or is immediately life-threatening and does not require hospital admission but which, based on medical judgment, involves a risk for the patient and requires medical or surgical intervention to prevent one of the above listed outcomes (patient death, life-threatening, hospital admission or prolongation of an existing hospitalization, congenital anomaly, or persistent or significant disability or incapacity). Examples of such events may include intensive treatment of an allergic bronchospasm at an emergency room or at the patient home, blood dyscrasies or seizures not leading to hospital admission of the patient, or development of drug dependence or abuse.

Severity

The physician will use the following definitions to rate the severity of each AE of special interest recorded as a study endpoint and for all SAEs.

Mild	The adverse event is transient and easily tolerated by the patient.
Moderate	The adverse event causes discomfort to the patient and interferes with his usual activities.
Severe	The adverse event substantially interferes with the usual activities of the patient and may be disabling or life-threatening.

Relationship with the drug

The physician will use the following definitions to establish the causal relationship of each AE:

Probably related	The adverse event has a clear temporal relationship with the drug or recurs on drug rechallenge, and other cause is unlikely or significantly less likely.
Possibly related	The adverse event has a marked temporal relationship with the drug and is equally likely or less likely that another cause exists as compared to the possible relationship with the study drug.
Probably unrelated	The adverse event has a minimal or no temporal relationship with the drug or another more likely cause exists, or both.
Unrelated	The adverse event is due to an underlying or concomitant disease or to the effect of another drug, and is not related to the drug (e.g. has no temporal relationship to the drug or another much more likely cause exists).

Adverse event collection period

SAEs will be reported from signature of informed consent and up to 30 days after the last dose of medication as described below. The same reporting procedure should be followed for any late SAE (occurring after this 30-day period) that is possibly or probably related to study medication.

Serious adverse event reporting

If a SAE occurs, whether or not related to the investigational drug, the investigator will report to the Sponsor by telephone or fax any serious adverse events occurring during the study, regardless of their causal relationship, within 24 h of awareness of the event or in the next working day. The investigator must expeditely report to the sponsor any SAEs that are fatal or life-threatening:

Fax no.: 0034-*915791580*

Telephone no.: 0034-*912948910*

All SAE reports by telephone must be followed by the corresponding written report form within 48 hours. For fax reports, the investigator will record the information referring to the SAE in the relevant form.

The minimum initial information for reporting an adverse event should include the following:
- adverse event description and onset date.
- patient code, sex, and age.
- information about the treatment received.
- name and address of the reporting physician.
- whether or not a causal relationship to the study drugs is considered to exist.

If death of a participating subject has been reported, the investigator will provide the Sponsor and CRECs involved with any additional information requested.

The form for reporting SAEs to the sponsor is enclosed as Appendix 4.

The Sponsor is responsible for reporting all suspected SUSARs within the times established by the applicable Spanish regulations to:

- The Spanish Agency for Medicinal Products and Medical Devices (AEMPS)
- The Clinical Research Ethics Committee (CREC) involved in the trial for SUSARs occurring in participating subjects from the sites of their area of influence.
- The relevant bodies of the autonomous communities when the suspected SUSAR has occurred in the healthcare centers of the corresponding region.
- The principal investigators.

The Sponsor also agrees to report to the pharmacovigilance department of the marketing authorization holders of the study drugs in Spain any serious or unexpected adverse events according to the reporting procedures.
Appendix D, provided by the AEMPS in the clarifications on the application of the clinical trial regulations from May 1, 2004 (version no. 4, 30 November 2006), will be used for reporting SUSARs to the CRECs and regulatory authorities.

9.2. MANAGEMENT OF TOXICITY:

All clinically significant laboratory toxicities will be managed according to the ACTG classification of adverse event severity (version 1.0 December 2004) (Appendix 5).

10. ETHICAL ISSUES

10.1 GENERAL CONSIDERATIONS

This study will be performed in compliance with the principles established in the Declaration of Helsinki and its subsequent revisions (Appendix 2) and the applicable Spanish regulations on clinical trials.

The study will be conducted according to the ICH Good Clinical Practice guidelines.

An insurance policy has been contracted for the study with the company HDI Hannover Internacional (España) Seguros y Reaseguros, S.A., in compliance with the provisions in Royal Decree 223/2004.

10.2 OBTAINING INFORMED CONSENT

Before any study-specific procedure is performed, the patient will be informed about the nature of the study treatment and will be provided with any relevant information about the intended objectives, potential benefits, and potential adverse events. The procedures to be followed and the potential risks will be explained to the patient. The patient must read and sign the approved informed consent form. Once signed and dated by the patient and the investigator, the patient will be given a copy of the signed informed consent. The patient may withdraw from the study at any time, without this affecting his or her future medical care. The patient information sheet and informed consent for the study is enclosed as Appendix 3.

11. PRACTICAL CONSIDERATIONS

11.1 RESPONSIBILITIES OF STUDY PARTICIPANTS

11.1.1. Rules for the patient

The patient must follow the instructions of investigators and report any events to them.

The patient will be duly informed of any prohibitions or restrictions to be respected during the study. Non-compliance with these recommendations will result in study withdrawal.

Patients may terminate their participation in the study at any time.

11.1.2. Rules for the investigating staff

The investigator agrees to comply with the rules set forth in the applicable clinical trial regulations: Medicines Act 2006 (Official State Journal no. 178, 27.07.06) and Royal Decree 223/2004 on Clinical Trials.

11.2 DATA MANAGEMENT AND FILE

The management, reporting and transfer of personal data from all participating subjects will comply with the Spanish Personal Data Protection Act 15/1999, of December 13.

On enrolment, each patient will be assigned a patient number, that will be recorded in the CRF.

Copies of all relevant information will be kept by the investigator and Sponsor in accordance to the provisions in Article 17 of Directive 2005/28/EC for at least 5 years after study completion or for a longer time if so required by the applicable local regulations or any agreement between the Sponsor and the Investigator.

The investigator will be responsible for keeping adequate information about each patient so that regulatory authorities may have access to such information if required. These records should be kept confidential for the time period legally established in the applicable regulations.

11.3 PUBLICATION CONDITIONS

The sponsor will review all requests for publication of the results of this study. The sponsor recognizes the importance of dissemination of results and therefore agrees to their partial or total disclosure by the principal investigators, provided the manuscript has previously been approved by the sponsor. The sponsor may require changes considered necessary for ensuring quality and protecting intellectual property. The principal investigator understands and agrees that, under certain conditions, publication of results will have to be delayed to ensure the scientific quality and integrity of data; for instance, until results are available in a number of sites representative of the study. The principal investigator understands and agrees that the sponsor has the right to decide the exact time when data may be disclosed. By signing this protocol, the principal investigator accepts the terms of the publication policy of the sponsor and agrees to abide by them.

11.4 PROTOCOL AMENDMENT PROCEDURE

Any protocol change should be documented in writing as an amendment.

Amendments should be duly identified by their chronological order number, and dated and signed by the sponsor and investigator.

If changes are relevant, the sponsor must request approval from the reference CREC and AEMPS, as set down in the applicable regulations.

The sponsor will be responsible for keeping a log of any changes that do not meet the criteria for a relevant amendment, as required in the applicable regulations, and monitor their implementation at the trial sites. Such log should be available in the event of an inspection or audit.

11.5 CLINICAL RESEARCH ETHICS COMMITTEE (CREC)

The protocol and informed consent form will be reviewed by a duly formed CREC. The decision of the CREC on the study conduct will be provided to the investigator in writing; a copy of this decision should be provided to the sponsor. The CRECs of participating centers will also assess the patient information sheet and the suitability of the investigating staff of their own hospitals.

The sponsor will submit the required study progress reports to the CREC and will report any suspected unexpected serious adverse reactions. At the end of the study, the sponsor should report them to the CREC.

12. STATISTICAL ANALYSIS

The primary analysis of the study will be performed at the end of the 6 months of follow-up in all patients.

Sample size and rationale

In a similar study population (e.g. HIV+ patients with an undetectable viral load, i.e. < 50 copies/mL of HIV-1 RNA), the mean baseline quality of life score measured with the MOS-HIV questionnaire was 61.84 **[24]**. As standard deviation is not available, we assume for all calculations that standard deviation will be the highest score minus the lowest score in the quality of life dimension (e.g. 100) divided by 5, which gives 20. This standard deviation of 20 is the same as found in HIV-infected asymptomatic patients, patients with AIDS-related symptoms, or patients with AIDS and a CD4 cell count <100 **[25].** Assuming, then, that quality of life will remain unchanged in patients assigned to the control group (maintaining current antiretroviral therapy) and will improve by 10% (e.g. to scores of 68.02) in patients assigned to the experimental group (LPV/r® as monotherapy), with a 1:2 ratio, an 80% statistical power, and a two-sided alpha of 0.05, 372 patients would be required, 248 randomized to the experimental group and 124 randomized to continue on the current antiretroviral therapy.
Assuming 7% follow-up losses, the final sample size required will be of 260 patients for the experimental group and 130 patients for the control group, i.e. a total of 390 patients.

Because the triple antiretroviral therapy based on a PI boosted with ritonavir (control treatment) has been widely studied for a practical sense, it has been considered to allocate the sample to twice the experimental treatment (1:2, respectively) **[24], [26].**

This distribution of the sample (1:2) compared with the distribution of equal groups (1:1) ensures greater security in the contrast of the study to a possible greater variability of response in the quality of life in the experimental group since this group is expected to more patients with diarrhea to begin new therapy. **[27]**

Also it will provide an improved accuracy in estimating mean and smaller confidence intervals, for example, more than 1% in the estimated proportions near 50%.

12.1. Population for analysis

A. Intention-to-treat population (ITT)

A.1. Population for the main analysis

This population includes all subjects in the control group that were included in the study and all subjects in the experimental group who have taken at least one dose of Kaletra ®. This analysis will provide estimates of treatment effect that will reflect the usual practice with greater accuracy.

A.2. Population for the secondary analysis

This population includes all subjects covered in "A.1.", but excluding those patients who have left the study for adverse events (suffer diarrhea). This analysis will assess the likely increase in difference in quality of life that is believed to provide a higher level of neglect in the experimental group because of an expected adverse event, which will give more optimistic estimates of the difference between treatments

B. Per protocol population (PP):

This population will include all patients who complete the 24 weeks of follow-up.

C. Safety population:

This population will include all subjects who have received at least one dose of study drug.

12.2. Primary endpoint

- To compare the quality of life from baseline to 24 weeks in patients who start monotherapy with LPV/r tablets versus patients on triple therapy containing any boosted protease inhibitor (PI) using the Medical Outcomes Study HIV Health Survey (MOS-HIV) and the visual analog scale EuroQol 5D (EQ-5D).

12.3. Secondary endpoints

- Assessment of treatment satisfaction of HIV+ patients who start monotherapy with LPV/r tablets versus triple therapy containing any PI using the CESTA questionnaire (Spanish Questionnaire of Satisfaction with Antiretroviral Treatment) from the baseline visit to study week 24.

- Assessment of treatment adherence by HIV+ patients who start monotherapy with LPV/r tablets versus triple therapy containing any PI using the GEEMA questionnaires and a visual analog scale (VAS).
- Assessment of regimen tolerability and safety in HIV+ patients who start monotherapy with LPV/r tablets versus triple therapy containing any PI.
- Proportion of patients with a viral load <50 copies/mL of HIV RNA at 24 weeks in HIV+ patients who start monotherapy with LPV/r tablets versus triple therapy containing any PI. A viral load >500 copies/mL of viral RNA (HIV-1) confirmed at study week 24 will be considered as virological failure.
- Changes in immune response in HIV+ patients who start monotherapy with LPV/r tablets versus triple therapy containing any PI, as measured through the change in CD4+ cell count from the baseline visit to study week 24

12.4. Statistical methods.

The statistical tests to be used and the specific study of each variable included will be described in detail in the Statistical Analysis Plan that will be prepared before the database is locked. However, a brief description is given below.
All analyses will be performed using statistical software SPSS, version 15.0 or higher. All statistical tests will be two-sided, with a significance level of 0.05.

Statistical analysis

Baseline characteristics of study groups will be compared using an analysis of variance (ANOVA). The mean, median, standard deviation, and range will be provided for continuous variables, and the distribution of absolute frequencies and percentages will be given for discrete variables. When required, 95% confidence intervals will be calculated. Discrete variables will be compared using a Chi-square test.
For quality of life analysis, the primary population will include all patients with data available from baseline up to at least one post-baseline visit (e.g. the intention-to-treat population).

In case of patient abandonment is taken as a post-treatment evaluation the one obtained at the time of abandonment. If there is no post-treatment evaluation as a neutral value, baseline observation value will be carried forward until month 24 or the time of abandonment (Baseline Observation Carried Forward).

The main deadline to study results will be week 24 of the study. An analysis of covariance (ANCOVA) will be used for quality of life comparison. The model will include the baseline values for the items in the MOS-HIV questionnaire, age and sex as covariates, in the control and study groups. For primary endpoint analysis, the values in the MOS-HIV and EQ-5D questionnaires in the different weeks as compared to baseline will be provided for each treatment group.

An analysis of covariance (ANCOVA) will be used for comparisons between both treatment groups.

For secondary endpoint analysis, a descriptive analysis will be done depending to variable distribution.

The mean, median, standard deviation, minimum, maximum, and confidence interval (95%) will be provided for the mean score in the visual analog scale (VAS) and GEEMA, and for each treatment group on treatment adherence.

The distribution of absolute and percent frequencies of the degree of perception in each of the questions will also be provided for each patient group, according to the questionnaire of patient satisfaction with treatment (CESTA)..

REFERENCES

1- Hammer SM, Saag MS, Schechter M, Montaner JS, Schooley RT, Jacobsen DM, Thompson MA, Carpenter CC, Fischl MA, Gazzard BG, Gatell JM, Hirsch MS, Katzenstein DA, Richman DD, Vella S, Yeni PG, Volberding PA; International AIDS Society-USA panel. Treatment for adult HIV infection: 2006 recommendations of the International AIDS Society-USA panel. JAMA. 2006 Aug 16;296(7):827-43.

2- Ammassari A, Trotta MP, Murri R, Castelli F, Narciso P, Noto P, Vecchiet J, D'Arminio Monforte A, Wu AW, Antinori A; AdICoNA Study Group. Correlates and predictors of adherence to highly active antiretroviral therapy: overview of published literature. J Acquir Immune Defic Syndr. 2002 Dec 15;31 Suppl 3:S123-7

3- Van der Valk M, Gisolf EH, Reiss P, Wit FW, Japour A, Weverling GJ, Danner SA; Prometheus study group. Increased risk of lipodystrophy when nucleoside analogue reverse transcriptase inhibitors are included with protease inhibitors in the treatment of HIV-1 infection. AIDS. 2001 May 4;15(7):847-55.

4- Giordano TP, Suarez-Almazor ME, Grimes RM. The population effectiveness of highly active antiretroviral therapy: are good drugs good enough? Curr HIV/AIDS Rep. 2005 Nov;2(4):177-83.

5- Spire B, Marcellin F, Cohen Codar I, et al. Impact of a lopinavir/ritonavir (LPV/r) monotherapy on self-reported side effects and global health perception among antiretroviral-naive patients: 48-week analysis of the MONARK Trial. 8th Annual International Congress on Drug Therapy in HIV Infection (HIV8). Glasgow, UK. November 12-16, 2006. Abstract PL13.3

6-Van der Valk M, Gisolf EH, Reiss P et al. Increased risk of lipodystrophy when nucleoside analogue reverse transcriptase inhibitors are included with protease inhibitors in the treatment of HIV infection. AIDS 2001;15:847-55

7-Falco V, Rodriguez D, Ribera E et al. Severe nucleoside-associated lactic acidosis in human immunodeficiency virus-infected patients: report of 12 cases and review of the literature. Clin Infect Dis. 2002;34:838-46.

8- Norvir Package Insert 2007.

9- Tam and Walmsley 2007.

10- Kempf DJ, Isaacson JD, King MS, Brun SC, Xu Y, Real K, Bernstein BM, Japour AJ, Sun E, Rode RA. Identification of genotypic changes in human immunodeficiency virus protease that correlate with reduced susceptibility to the protease inhibitor lopinavir among viral isolates from protease inhibitor-experienced patients. J Virol 2001; 75: 7462-7469.

11-King MS, Rode R, Cohen-Codar I, Calvez V, Marcelin AG, Hanna GJ, Kempf DJ. Predictive genotypic algorithm for virologic response to lopinavir-ritonavir in protease inhibitor-experienced patients. Antimicrob Agents Chemother 2007; 51: 3067-74.

12- Walmsley S, Bernstein B, King M, Arribas J, Beall G, Ruane P, Johnson M, Johnson D, Lalonde R, Japour A, Brun S, Sun E; M98-863 Study Team. Lopinavir-ritonavir versus nelfinavir for the initial treatment of HIV infection. N Engl J Med. 2002 Jun 27;346 (26):2039-46.

13-JF Delfraissy, P Flandre, C Delaugerre, et al. “MONARK Trial (MONotherapy AntiRetroviral Kaletra): 48-Week Interim Analysis of LPV/r Monotherapy compared to LPV/r + AZT/3TC in Antiretroviral-Naïve Patients” THLB0202. Oral LB. 16th IAC 2006. Toronto (Canada)

14-Cameron DW, da Silva B, Arribas JR, Pulido F, Katner H, Wikstrom K, et al. Significant sparing of peripheral lipoatrophy by HIV treatment with LPV/r + ZDV/3TC induction followed by LPV/r monotherapy compared with EFV + ZDV/3TC. Program and abstracts of the 14th Conference on Retrovirus and Opportunistic Infections; February 25-28, 2007; Los Angeles, California. Abstract 44.

15-Pulido F, Arribas JR, Delgado R, Cabrero E, González-García J, Pérez-Elias MJ, Arranz A, Portilla J, Pasquau J, Iribarren JA, Rubio R, Norton M; OK04 Study Group. Lopinavir-ritonavir monotherapy versus lopinavir-ritonavir and two nucleosides for maintenance therapy of HIV. AIDS 2008; 22: F1-F9.

16-Arribas JR, Pulido F, Delgado R, Lorenzo A, Miralles P, Arranz A, González-García JJ, Cepeda C, Hervás R, Paño JR, Gaya F, Carcas A, Montes ML, Costa JR, Peña JM. Lopinavir/ritonavir as single-drug therapy for maintenance of HIV-1 viral suppression: 48-

week results of a randomized, controlled, open-label, proof-of-concept pilot clinical trial (OK Study). J Acquir Immune Defic Syndr. 2005;40: 280-7.

17-Nunes EP, Oliveira MS, Almeida MMTB, Pilotto JH, Ribeiro JE, Faulhaber JC et al. 48-week efficacy and safety results of simplification to single agent lopinavir/ritonavir regimen in patients suppressed below 80 copies/ml on HAART - the KalMo study. Sixteenth International AIDS Conference, Toronto, abstract TuAb0103, 2006.

18-Arribas JR, Pulido F, Delgado R, González-García J, Pérez-Elias MJ, Arranz A, Portilla J, et al OK04 Study Group. Lopinavir-ritonavir monotherapy versus lopinavir-ritonavir and two nucleosides for maintenance therapy of HIV. Ninety-six Week Results of a Randomized, Controlled, Open Label, Clinical Trial (OK04 Study). 11th European AIDS Conference/EACS; October 24-27. 2007. Abstract PS3/1.

19- Recomendaciones de Gesida y Plan Nacional sobre el Sida sobre el tratamiento antirretroviral del adulto (enero, 2008).

20- Badia X, Roset M, Montserrat S, Herdman M, Segura A. [The Spanish version of EuroQol: a description and its applications. European Quality of Life scale]. Med Clin (Barc). 1999;112 Suppl 1:79-85.

21- Condes E, Aguirrebengoa K, Dalmau D, Estrada JM, Force L, Górgolas M, Badia X, Podzamczer D. [Validation of a questionnaire to estimate satisfaction with antiretroviral treatment: CESTA questionnaire] Enferm Infecc Microbiol Clin. 2005 Dec;23(10):586-92.

22- Giordano TP, Guzman D, Clark R, Charlebois ED, Bangsberg DR. Measuring adherence to antiretroviral therapy in a diverse population using a visual analogue scale. HIV Clin Trials. 2004 Mar-Apr;5(2):74-9.

23- Knobel H, Alonso J, Casado JL, Collazos J, Gonzalez J, Ruiz I, Kindelan JM, Carmona A, Juega J, Ocampo A; GEEMA Study Group. Validation of a simplified medication adherence questionnaire in a large cohort of HIV-infected patients: the GEEMA Study. AIDS. 2002 Mar 8;16(4):605-13.

24- Sprinz E, Neto AJ, Bargman E, Green SL, Luo MP, Sylte JR, McMillan FI, King KR, Rode RA, Brun SC, Hanna GJ, Podsadecki TJ. Substitution with lopinavir/ritonavir improves patient-reported outcomes including quality of life in patients who were intolerant to their antiretroviral therapy. HIV Clin Trials. 2006 Nov-Dec;7(6):291-308.

25- Wu AW, Revicki DA, Jacobson D, Malitz FE. Evidence for reliability, validity and usefulness of the Medical Outcomes Study HIV Health Survey (MOS-HIV). Qual Life Res. 1997 Aug;6(6):481-93.

26- Johnson MA, Gathe JC Jr, Podzamczer D, Molina JM, Naylor CT, Chiu YL, King MS, Podsadecki TJ, Hanna GJ, Brun SC. A once-daily lopinavir/ritonavir-based regimen provides noninferior antiviral activity compared with a twice-daily regimen. J Acquir Immune Defic Syndr. 2006 Oct 1;43(2):153-60.

27- Martín Andrés A. y Luna del Castillo J. de D. (1999), Bioestadística para las Ciencias de la Salud, Ediciones Norma.).

PROTOCOL APPENDICES

Appendix 1 - Protocol signature document

Appendix 2 - Declaration of Helsinki of the World Medical Association

Appendix 3 – Patient Information Sheet and Informed Consent

Appendix 4 – Serious adverse event reporting form

Appendix 5 – ACTG Classification of adverse event severity in adults

Appendix 6 - MOS-HIV, EQ-5D, CESTA, VAS, and GEEMA questionnaires.

APPENDIX 1

Protocol signature document for the principal investigator

Mr/Ms:

Department:

Site:

States,

That he or she has evaluated the clinical trial protocol entitled: **"A randomized, open label clinical trial to compare the quality of life of HIV+ patients who start monotherapy with LPV/r tablets versus triple therapy containing a boosted PI"**

Sponsor code: **SAI-CDV-2009-01**

EudraCT No.: **2009-014430-25**

Version dated July 24th 2009.

Sponsored by the Andalusian Society of Infectious Diseases (SAEI)

That the clinical trial abides by the ethical rules applicable to this type of study.

That he or she agrees to participate as principal investigator in this clinical trial.

That he or she has the necessary material and human resources to conduct the clinical trial, without this interfering with the conduct of other types of studies or other tasks usually discharged.

That he or she agrees to manage and monitor each patient in compliance with the provisions in the protocol, which has received the favorable opinion of the Clinical Research Ethics Committee and has been approved by the Spanish Agency for Medicinal Products and Medical Devices.

That he/she will abide by the ethical and legal rules applicable to this type of study and will follow good clinical practice guidelines for its conduct.

That the subinvestigators required to conduct the proposed clinical trial are suitable.

In ______________________ on__________________

Signed:

Mr/Ms__________________

Principal Investigator

APPENDIX 2

WORLD MEDICAL ASSOCIATION DECLARATION OF HELSINKI

Ethical Principles for Medical Research Involving Human Subjects

Adopted by the 18th WMA General Assembly, Helsinki, Finland, June 1964, and amended by the:
29th WMA General Assembly, Tokyo, Japan, October 1975
35th WMA General Assembly, Venice, Italy, October 1983
41st WMA General Assembly, Hong Kong, September 1989
48th WMA General Assembly, Somerset West, Republic of South Africa, October 1996
52nd WMA General Assembly, Edinburgh, Scotland, October 2000 53th WMA General Assembly, Washington 2002 (Note of Clarification on paragraph 29 added)
55th WMA General Assembly, Tokyo 2004 (Note of Clarification on Paragraph 30 added)
59th WMA General Assembly, Seoul, October 2008

A. INTRODUCTION

1. The World Medical Association (WMA) has developed the Declaration of Helsinki as a statement of ethical principles for medical research involving human subjects, including research on identifiable human material and data.

The Declaration is intended to be read as a whole and each of its constituent paragraphs should not be applied without consideration of all other relevant paragraphs.

2. Although the Declaration is addressed primarily to physicians, the WMA encourages other participants in medical research involving human subjects to adopt these principles.

3. It is the duty of the physician to promote and safeguard the health of patients, including those who are involved in medical research. The physician's knowledge and conscience are dedicated to the fulfilment of this duty.

4. The Declaration of Geneva of the WMA binds the physician with the words, “The health of my patient will be my first consideration,” and the International Code of Medical Ethics declares that, “A physician shall act in the patient's best interest when providing medical care.”

5. Medical progress is based on research that ultimately must include studies involving human subjects. Populations that are underrepresented in medical research should be provided appropriate access to participation in research.

6. In medical research involving human subjects, the well-being of the individual research subject must take precedence over all other interests.

7. The primary purpose of medical research involving human subjects is to understand the causes, development and effects of diseases and improve preventive, diagnostic and therapeutic interventions (methods, procedures and treatments). Even the best current interventions must be evaluated continually through research for their safety, effectiveness, efficiency, accessibility and quality.

8. In medical practice and in medical research, most interventions involve risks and burdens.

9. Medical research is subject to ethical standards that promote respect for all human subjects and protect their health and rights. Some research populations are particularly vulnerable and need special protection. These include those who cannot give or refuse consent for themselves and those who may be vulnerable to coercion or undue influence.

10. Physicians should consider the ethical, legal and regulatory norms and standards for research involving human subjects in their own countries as well as applicable international norms and standards. No national or international ethical, legal or regulatory requirement should reduce or eliminate any of the protections for research subjects set forth in this Declaration.

B. PRINCIPLES FOR ALL MEDICAL RESEARCH

11. It is the duty of physicians who participate in medical research to protect the life, health, dignity, integrity, right to self-determination, privacy, and confidentiality of personal information of research subjects.

12. Medical research involving human subjects must conform to generally accepted scientific principles, be based on a thorough knowledge of the scientific literature, other relevant sources of information, and adequate laboratory and, as appropriate, animal experimentation. The welfare of animals used for research must be respected.

13. Appropriate caution must be exercised in the conduct of medical research that may harm the environment.

14. The design and performance of each research study involving human subjects must be clearly described in a research protocol. The protocol should contain a statement of the ethical considerations involved and should indicate how the principles in this Declaration have been addressed. The protocol should include information regarding funding, sponsors, institutional affiliations, other potential conflicts of interest, incentives for subjects and provisions for treating and/or compensating subjects who are harmed as a consequence of

participation in the research study. The protocol should describe arrangements for post-study access by study subjects to interventions identified as beneficial in the study or access to other appropriate care or benefits.

15. The research protocol must be submitted for consideration, comment, guidance and approval to a research ethics committee before the study begins. This committee must be independent of the researcher, the sponsor and any other undue influence. It must take into consideration the laws and regulations of the country or countries in which the research is to be performed as well as applicable international norms and standards but these must not be allowed to reduce or eliminate any of the protections for research subjects set forth in this Declaration. The committee must have the right to monitor ongoing studies. The researcher must provide monitoring information to the committee, especially information about any serious adverse events. No change to the protocol may be made without consideration and approval by the committee.

16. Medical research involving human subjects must be conducted only by individuals with the appropriate scientific training and qualifications. Research on patients or healthy volunteers requires the supervision of a competent and appropriately qualified physician or other health care professional. The responsibility for the protection of research subjects must always rest with the physician or other health care professional and never the research subjects, even though they have given consent.

17. Medical research involving a disadvantaged or vulnerable population or community is only justified if the research is responsive to the health needs and priorities of this population or community and if there is a reasonable likelihood that this population or community stands to benefit from the results of the research.

18. Every medical research study involving human subjects must be preceded by careful assessment of predictable risks and burdens to the individuals and communities involved in the research in comparison with foreseeable benefits to them and to other individuals or communities affected by the condition under investigation.

19. Every clinical trial must be registered in a publicly accessible database before recruitment of the first subject.

20. Physicians may not participate in a research study involving human subjects unless they are confident that the risks involved have been adequately assessed and can be satisfactorily managed. Physicians must immediately stop a study when the risks are found to outweigh the potential benefits or when there is conclusive proof of positive and beneficial results.

21. Medical research involving human subjects may only be conducted if the importance of the objective outweighs the inherent risks and burdens to the research subjects.

22. Participation by competent individuals as subjects in medical research must be voluntary. Although it may be appropriate to consult family members or community leaders, no competent individual may be enrolled in a research study unless he or she freely agrees.

23. Every precaution must be taken to protect the privacy of research subjects and the confidentiality of their personal information and to minimize the impact of the study on their physical, mental and social integrity.

24. In medical research involving competent human subjects, each potential subject must be adequately informed of the aims, methods, sources of funding, any possible conflicts of interest, institutional affiliations of the researcher, the anticipated benefits and potential risks of the study and the discomfort it may entail, and any other relevant aspects of the study. The potential subject must be informed of the right to refuse to participate in the study or to withdraw consent to participate at any time without reprisal. Special attention should be given to the specific information needs of individual potential subjects as well as to the methods used to deliver the information. After ensuring that the potential subject has understood the information, the physician or another appropriately qualified individual must then seek the potential subject's freely-given informed consent, preferably in writing. If the consent cannot be expressed in writing, the non-written consent must be formally documented and witnessed.

25. For medical research using identifiable human material or data, physicians must normally seek consent for the collection, analysis, storage and/or reuse. There may be situations where consent would be impossible or impractical to obtain for such research or would pose a threat to the validity of the research. In such situations the research may be done only after consideration and approval of a research ethics committee.

26. When seeking informed consent for participation in a research study the physician should be particularly cautious if the potential subject is in a dependent relationship with the physician or may consent under duress. In such situations the informed consent should be sought by an appropriately qualified individual who is completely independent of this relationship.

27. For a potential research subject who is incompetent, the physician must seek informed consent from the legally authorized representative. These individuals must not be included in a research study that has no likelihood of benefit for them unless it is intended to promote the health of the population represented by the potential subject, the research cannot instead be

performed with competent persons, and the research entails only minimal risk and minimal burden.

28. When a potential research subject who is deemed incompetent is able to give assent to decisions about participation in research, the physician must seek that assent in addition to the consent of the legally authorized representative. The potential subject's dissent should be respected.

29. Research involving subjects who are physically or mentally incapable of giving consent, for example, unconscious patients, may be done only if the physical or mental condition that prevents giving informed consent is a necessary characteristic of the research population. In such circumstances the physician should seek informed consent from the legally authorized representative. If no such representative is available and if the research cannot be delayed, the study may proceed without informed consent provided that the specific reasons for involving subjects with a condition that renders them unable to give informed consent have been stated in the research protocol and the study has been approved by a research ethics committee. Consent to remain in the research should be obtained as soon as possible from the subject or a legally authorized representative.

30. Authors, editors and publishers all have ethical obligations with regard to the publication of the results of research. Authors have a duty to make publicly available the results of their research on human subjects and are accountable for the completeness and accuracy of their reports. They should adhere to accepted guidelines for ethical reporting. Negative and inconclusive as well as positive results should be published or otherwise made publicly available. Sources of funding, institutional affiliations and conflicts of interest should be declared in the publication. Reports of research not in accordance with the principles of this Declaration should not be accepted for publication.

C. ADDITIONAL PRINCIPLES FOR MEDICAL RESEARCH COMBINED WITH MEDICAL CARE

31. The physician may combine medical research with medical care only to the extent that the research is justified by its potential preventive, diagnostic or therapeutic value and if the physician has good reason to believe that participation in the research study will not adversely affect the health of the patients who serve as research subjects.

32. The benefits, risks, burdens and effectiveness of a new intervention must be tested against those of the best current proven intervention, except in the following circumstances:

- The use of placebo, or no treatment, is acceptable in studies where no current proven intervention exists; or
- Where for compelling and scientifically sound methodological reasons the use of placebo is necessary to determine the efficacy or safety of an intervention and the patients who receive placebo or no treatment will not be subject to any risk of serious or irreversible harm. Extreme care must be taken to avoid abuse of this option.

33. At the conclusion of the study, patients entered into the study are entitled to be informed about the outcome of the study and to share any benefits that result from it, for example, access to interventions identified as beneficial in the study or to other appropriate care or benefits.

34. The physician must fully inform the patient which aspects of the care are related to the research. The refusal of a patient to participate in a study or the patient's decision to withdraw from the study must never interfere with the patient-physician relationship.

35. In the treatment of a patient, where proven interventions do not exist or have been ineffective, the physician, after seeking expert advice, with informed consent from the patient or a legally authorized representative, may use an unproven intervention if in the physician's judgement it offers hope of saving life, re-establishing health or alleviating suffering. Where possible, this intervention should be made the object of research, designed to evaluate its safety and efficacy. In all cases, new information should be recorded and, where appropriate, made publicly available.

APPENDIX 3

PATIENT INFORMATION SHEET AND INFORMED CONSENT

Patient information sheet

"A randomized, open label clinical trial to compare the quality of life of HIV+ patients who start monotherapy with LPV/r tablets versus triple therapy containing a boosted PI"

Sponsor: Andalusian Society of Infectious Diseases (SAEI)

1. STUDY OBJECTIVE AND PURPOSE

You are infected with the human immunodeficiency virus (HIV-1). Your doctor has invited you to participate in a clinical research trial in which approximately 390 patients diagnosed of HIV-1 in Spain are planned to be included.

In this trial, that is sponsored by the Andalusian Society of Infectious Diseases (SAEI), we offer you the possibility of assessing the impact on quality of life of maintaining a triple therapy treatment (the one you are now taking) or switching to a single antiretroviral treatment (LPV/r, Kaletra®).

Before you take a decision, you should know what is expected from you during the trial. Read this Patient Information Sheet carefully and ask your doctor any questions you may have. Take all the time you need to decide whether or not you would like to take part in this trial.

The purpose of this trial is to document the changes in quality of life. It is intended to verify whether the course of changes occurring in your quality of life as the result of your disease and the two treatments compared in the trial may be changed. This trial will investigate a treatment based on switching from your current therapy to use of Kaletra® (LPV/r) as monotherapy.

This possibility of switching to Kaletra® after being for at least 6 months with an undetectable viral load and without showing protease gene resistance has been previously tested, and results have been shown to be not inferior to those of triple therapy with Kaletra® in terms of virological and immunological efficacy.

Nobody can force you to take part in this trial. If you want to take part, keep this Patient Information Sheet in case you need to read it in the future. In addition, as required by law, you should sign and date the enclosed informed consent form.

Participation in this clinical trial is voluntary. Even if you agree to take part now, you are free to change your mind and withdraw from it at a later time without any penalty or loss of the benefits to which you are otherwise entitled. Your doctor may also decide to withdraw you from the study at any time if, according to his or her medical opinion and expertise, he or she considers that you would benefit from discontinuation of the trial treatment.

The treatment of your disease or continuation of treatment once this trial has been completed are issues you will discuss with your doctor, who will inform you in detail about the available therapeutic options (other drugs with different mechanisms of action).

2. TRIAL MEDICATION

This clinical trial is designed as a randomized, comparative trial. A comparative trial means that two treatment groups will be compared. The purpose of this is to determine whether the dosing regimen based on your current treatment or Kaletra® as monotherapy show differences in terms of your quality of life. Before the start of the trial, participants will be assigned at random to the treatment group with the dosing regimen you are currently receiving or the treatment group with Kaletra® as monotherapy.

If you meet all requirements to enter this trial and agree to participate, you may receive one of the following dosing regimens:

- ☞ **Your current antiretroviral treatment may be switched to Kaletra® as monotherapy.**

  Kaletra contains 200 mg of lopinavir and 50 mg of ritonavir, two antiretroviral drugs that are already authorized. The dose of Kaletra is 2 tablets twice daily.

- ☞ **You may continue with your current dosing regimen without changes.**

  Medication will be administered in accordance to the relevant prescribing information.

Potential risks

Risks related to the interventions tests:

Treatment of HIV infection currently requires administration of at least three antiretroviral drugs. When you are assigned to the treatment simplification group and the drugs you are currently taking are changed, there is a risk that control of HIV infection is lost

and your viral load may become detectable again. There is also a risk that your virus becomes resistant to treatment with Lopinavir/ritonavir. For this reason, if a detectable viral load is found, your viral load will be measured again, and if an increase to more than 500 copies is confirmed, a resistance test will be performed to allow for advising the best treatment for you.

Other potential risks related to the study medication:

-- Lopinavir/ritonavir: The side effects most commonly reported in patients treated with Lopinavir/ritonavir (Kaletra®) include: diarrhea, nausea, increased plasma lipids (cholesterol and triglycerides), potential hepatic enzyme elevations, and risk of hepatitis.

During treatment with Kaletra you should not take any of the following medicines because they may cause severe problems or even death:

Antihistamines: terfenadine (Cyater®, Rapidal®, Ternadin®)

Sedatives/hypnotics: midazolam (Dormicum®) or triazolam (Halcion®)

Antimicrobials: rifampin (Rifaldin®, Rifater®)

Drugs for migraine: ergotamine (Cafergot®,Hemicraneal®), dihydroergotamine (Tonopan®), and methylergonovine (Methergin®)

Neuroleptics: pimozide (Orap®)

Antemetic/antidiarrheal drugs: cisapride (Prepulsid®, Arcasin®)

Lipid lowering drugs: lovastatin (Mevacor®) or simvastatin (Zocor®)

Herbal preparations: St. John wort

Antiarrhythmics: propafenone (Rytmonorm®) and flecainide (Apocard®)

It is important that you discuss with your doctor or a member of his/her staff any other medicines you are taking, because dosage of such medicines must sometimes be changed when they are taken with lopinavir/ritonavir.

You agree not to use drugs (including alcohol and prescription or over-the-counter medicines, herbal preparations, vitamin supplements, or illicit drugs) other than lopinavir/r without authorization from the study doctor. Treatment with drugs other than Lopinavir/ritonavir may cause severe and even life-threatening reactions.

-- Abacavir/Lamivudine: Even if you are tolerating your current treatment, nucleoside drugs (abacavir and lamivudine) may cause toxic effects of several types: anemia, nerve inflammation, and loss of body fat, among others.

You may also experience side effects that are unknown or not reported to date.

Because of its effect on viral load, you should tell your study doctor or staff if you have received a vaccine in the month prior to the study or if you are vaccinated at any time during the study.

Risks in special populations: Women of childbearing age:

Lopinavir/ritonavir is currently the PI of choice during pregnancy, but its use as monotherapy is not safe during pregnancy. Thus, if you are a woman of childbearing age, you will only be recruited into the study if you are not pregnant and are using a reliable barrier contraceptive method (in the opinion of the study doctor) or if you have no sexual activity. A pregnancy test will be done at baseline/day 1. You will not receive study treatment until a negative urine pregnancy test is available. Additional pregnancy tests may be performed during the study at discretion of the study doctor. If you become pregnant during this study, you must inform your study doctor immediately, and your participation in the study may be discontinued

Other risks:

Risks associated to blood sampling include: pain, bruising, bleeding, or other discomfort at the site where blood is taken. Anemia, fainting, or infection at the blood sampling site may rarely occur. Adequate precautions will be taken to minimize these risks.

New information:

You will be informed in writing of any significant new finding related to the study that may affect your safety or change your decision to participate in the study. If you decide to continue in this study after receiving the information, you will be asked to sign a new (revised) informed consent to document that you have received this new information.

3. ELIGIBILITY CRITERIA

To be able to participate in the trial, your doctor will review all inclusion/exclusion criteria and will tell you whether or not you can participate based on clinical judgment. You will then freely decide if you want to participate after you have been provided all information about the trial.

4. TRIAL PROCEDURES

The total duration of the trial is 24 weeks. If you agree to take part in the trial and you give your written informed consent (with your signature and the acceptance date), you will attend a screening visit, a baseline visit (where follow-up will be started and you will start taking the assigned medication or will continue with the medication you are currently taking), and visits at weeks 12 and 24 since the baseline visit. If you have started Kaletra® as monotherapy, you will return at week 4 after the baseline visit for virological and tolerability monitoring. These visits represent no change in the usual follow-up frequency of your doctor, except for the week 4 visit.

At these visits, the following assessments will be performed:

Screening visit

At this visit, if you have decided to take part in the trial, you must sign a document called an Informed Consent Form indicating that you want to participate in the trial voluntarily once you have been given all the information requested. You will then be asked some questions about your clinical history and will be performed a physical examination and blood tests to confirm that you meet all the criteria required for participating in the trial.

If you are a woman of childbearing age, you will be done a pregnancy test (urine test).

Baseline visit

Once the results of the screening visit are available, you will be given an appointment for the baseline study visit. At this visit, a physical examination and blood tests will be done again, and you will be informed of the anti-HIV treatment assigned. If recent results of blood tests are available (within the previous two months), your doctor may decide to perform the screening and baseline visits in a single visit.

At this visit, you must also complete a questionnaire on your currently perceived quality of life, your satisfaction with the treatment you are receiving, and your treatment adherence.

Follow-up and final visits

Your will return for a follow-up visit at week 12 and for a final visit at week 24 after the baseline visit. . If you have started Kaletra® as monotherapy, you will return at week 4 after the baseline visit for virological and tolerability monitoring.

At some of these visits, you will be required to complete a questionnaire on your currently perceived quality of life, your satisfaction with the treatment you are receiving, and your treatment adherence.

In addition, blood samples will be required for routine laboratory tests. At each visit, your doctor will ask you about any unusual signs or symptoms occurring since the last visit. You will also be asked if you have taken any medication other than the study medication since your last visit.

Your doctor will prescribe you your medication and will appoint you for the next visit.

In addition, at the final visit you will undergo a final assessment and your doctor will decide whether you should continue with the medication used during the trial or if a switch in treatment is required.

You should understand that there are some drugs that may be contraindicated with the trial treatment and that **you should not start any treatment** without previously consulting your trial doctor.

5. RISKS AND BENEFITS

Before designing this clinical trial, doctors and investigators have carefully weighed the benefits of the study treatment against its potential risks. To protect the patient, this trial will be conducted in full compliance with the principles of the World Medical Association, pursuant to the rules, directives and regulations of the European Union, and in strict compliance with the Spanish regulations on medicinal products and the Spanish personal data protection act.

Inform your doctor of any side effect:

The side effects most commonly related to the study drug are summarized below:

Very common side effects

(These may affect more than 1 out of every 10 patients treated)

- Headache, diarrhea, dizziness (nausea).

Common side effects

(These may affect from 1 to 10 out of every 100 patients treated)

- Vomiting, abdominal pain, stomach ache, abnormal stools.
- Difficulty sleeping, abnormal dreams, fever, malaise.
- A tingling sensation.
- Cough, nasal symptoms.
- Digestive problems resulting in discomfort after meals, flatulence.
- Rash, that may consist of allergic reactions, itching, skin discoloration.
- Other allergic reactions.
- Feeling of weakness.

The tests may also show:

- a low white blood cell count (a reduced white blood cell count can make you more susceptible to infection), low red blood cell count (anemia).
- increased blood sugar levels.
- hepatic and pancreatic problems.

Uncommon side effects

(These may affect less than 1 out of every 1,000 patients treated)

- pain in the abdomen (stomach) caused by pancreas inflammation.

- changes in urine and backache caused by renal problems.

The tests may also show:

- lactic acidosis (excess lactic acid in blood, a serious side effect that may be fatal). The following side effects may be signs of lactic acidosis:
    - deep, fast breathing.
    - drowsiness
    - dizziness (nausea), vomiting, and stomach ache.

  If you think you may have lactic acidosis, contact your doctor immediately.

Other potential effects

Combined antiretroviral treatment may change the shape of your body by modifying how your body fat is distributed. You may lose fat from the legs, arms, and face; fat may increase around the abdomen (stomach) and internal organs; breasts may be enlarged, or fatty lumps may appear in the back of your neck ("buffalo hump"). The reason and long-term effects of these changes are still unknown.

Combined antiretroviral treatment may also cause hyperlipidemia (increased blood fat) and insulin resistance. Your doctor will perform tests to establish these changes.

Remember that if you notice any other side effect should report it to your trial doctor.

You may ask any question about the potential and/or known risks of the trial at any time during the trial.

Patients treated with drugs to be used in the trial or with any other antiretroviral treatment may continue to develop opportunistic infections and other complications related to HIV.

The current antiretroviral dosing regimens, including the drugs used in this study, have not been shown to prevent the risks of HIV transmission to other people by the sexual route or by blood contamination and continued use of adequate precautions is therefore required.

In the event of pregnancy, you should inform your doctor immediately.

If you experience any problem after you start receiving the trial medication, contact your doctor immediately.

If you have any question about the adverse effects of the trial medication, do not hesitate to contact your doctor or refer to the corresponding package leaflets.

Your family doctor/general practitioner will be informed of your participation in the study with your consent.

Your participation in the trial may benefit you because you may experience an improvement, but you should know that your participation may provide no benefit, though it may help other HIV patients to benefit from it. In full compliance with the rules and regulations applicable to clinical research trials, your doctor will monitor your condition carefully in terms of treatment efficacy and potential side effects.

6. ALTERNATIVE TREATMENTS

There are other antiretroviral treatment options that you may discuss with your doctor.

If you participate in the trial and have to be withdrawn from it due to treatment failure, intolerance or toxicity, your doctor will inform you of the available alternative treatments.

7. ETHICAL ISSUES

This trial has been approved by a reference clinical research ethics committee and has also been reviewed by the ethics committees of all participating hospitals. The study has also been submitted to and reviewed and approved by the relevant regulatory authorities.

8. DATA CONFIDENTIALITY

Your participation in the trial will be confidential and nobody will know your name, except for the person responsible for the trial at your site.

The trial monitor(s), auditor(s), clinical research ethics committee members, and representatives from regulatory authorities will have direct access to your original clinical history to verify trial data and/or procedures, maintaining confidentiality at all times as required by law.

If the trial results are published in medical literature, you will not be identified by your name.

Legal regulations on data privacy and confidentiality will be respected at all times: Spanish Data Protection Organic Act 1999, of 13 December 1999, published in the Official State Journal no. 298, 14 December, 1999, pages 43088-43099.

9. PARTICIPATION CONDITIONS

Your participation in this clinical trial is completely voluntary. You are free to change your mind and withdraw from the study at a later time without any penalty or loss of the benefits to which you are otherwise entitled.

Dr………………………….. will inform you about any treatment alternatives he or she considers appropriate for your and about the procedure for discontinuing the treatment of the trial with no problems.

You understand that Dr …………………. may discontinue your participation in the clinical trial if you have not followed the study instructions, if withdrawal is in your best interest, or if the sponsor terminates the study. If any significant information is discovered about the study medication or the disease during the course of the clinical trial, you will be informed immediately in order to decide whether you want to continue or withdraw from the study.

10. STUDY INSURANCE

You should know that a civil liability insurance has been contracted by the sponsor with the company HDI Hannover Internacional (España) Seguros y Reaseguros, S.A.…………………………………………., Spanish branch, with registered office at……………………………… number …………………………………….. to cover potential injuries or damages, as required by the applicable law (Royal Decree 223/04).

11. ADDITIONAL INFORMATION

This trial will be conducted under the supervision of Dr ……………………………..

You may contact him/her whenever you wish. If you notice any side effects or you have any questions about any aspect of the study or your rights as a patient, do not hesitate to call……………………. or ………………………………….

WRITTEN INFORMED CONSENT FORM

"A randomized, open label clinical trial to compare the quality of life of HIV+ patients who start monotherapy with LPV/r tablets versus triple therapy containing a boosted PI"

I, (insert full name) ____

- Have read the information sheet given to me.
- Have been able to ask questions about the trial.
- Have received adequate information about the trial.
- Have talked to (name of investigator) – Dr.________________________________

I understand that my participation is voluntary.

I understand that I can withdraw from the trial:

1. Whenever I so wish.
2. Without having to give a reason.
3. Without this affecting my medical care.

I hereby freely give my consent to take part in the trial.

____

Signature of participant **Date**

____

Signature of investigator **Date**

CONSENT FORM FOR THE REPRESENTATIVE

"A randomized, open label clinical trial to compare the quality of life of HIV+ patients who start monotherapy with LPV/r tablets versus triple therapy containing a boosted PI"

I, (insert full name) .... as (relationship with participant) ………………………. of .... (full name of participant)

- Have read the information sheet given to me.
- Have been able to ask questions about the trial.
- Have received satisfactory answers to all my questions.
- Have received adequate information about the trial.
- Have talked to Dr. .... (name of investigator)
- I understand that participation of the patient is voluntary.
- I understand that he/she can withdraw from the trial:
  1. Whenever he/she so wishes.
  2. Without having to give a reason.
  3. Without this affecting his/her medical care.

In my presence, (name of participant) .... has been given all relevant information adapted to his/her level of understanding and agrees to take part.

And I hereby give my approval for participation of (full name of participant) .... in this study.

Signature of participant **Date**

Signature of investigator **Date**

APPENDIX 4

Serious adverse event reporting form

Attached as a separate document

APPENDIX 5

ACTG CLASSIFICATION OF ADVERSE EVENT SEVERITY IN ADULTS

Attached as a separate document

ANNEX 1

MANAGEMENT FLOW CHART FOR PATIENTS WITH VIROLOGICAL FAILURE

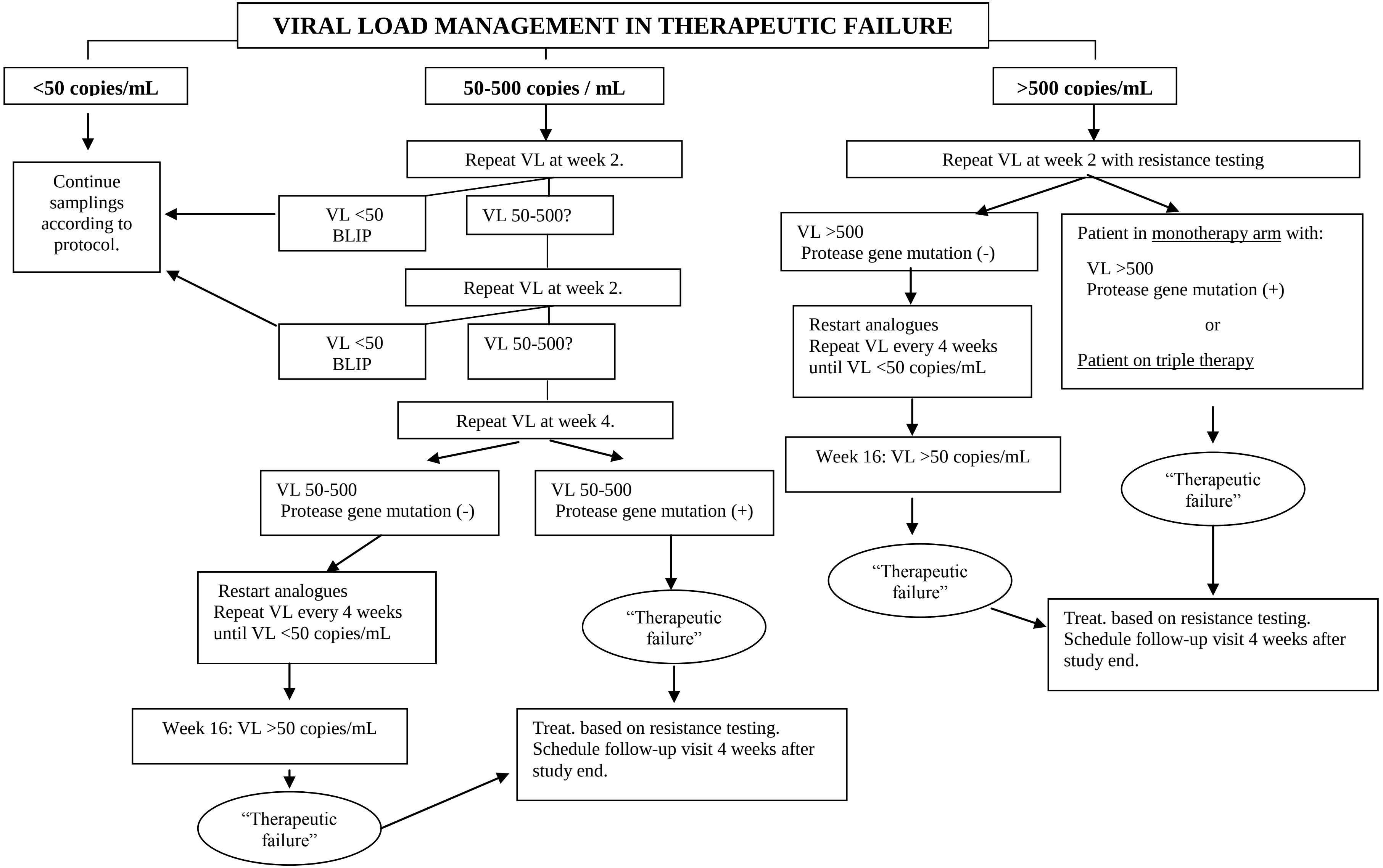
VIRAL LOAD MANAGEMENT IN THERAPEUTIC FAILURE
<50 copies/mL
50-500 copies / mL
>500 copies/mL
Continue samplings according to protocol.
Repeat VL at week 2.
VL <50 BLIP
VL 50-500?
Repeat VL at week 2.
VL <50 BLIP
VL 50-500?
Repeat VL at week 4.
VL 50-500 Protease gene mutation (-)
VL 50-500 Protease gene mutation (+)
Restart analogues Repeat VL every 4 weeks until VL <50 copies/mL
"Therapeutic failure"
Week 16: VL >50 copies/mL
Treat. based on resistance testing. Schedule follow-up visit 4 weeks after study end.
"Therapeutic failure"
Repeat VL at week 2 with resistance testing
VL >500 Protease gene mutation (-)
Patient in monotherapy arm with:
VL >500
Protease gene mutation (+)
or
Patient on triple therapy
Restart analogues Repeat VL every 4 weeks until VL <50 copies/mL
Week 16: VL >50 copies/mL
"Therapeutic failure"
"Therapeutic failure"
Treat. based on resistance testing. Schedule follow-up visit 4 weeks after study end.

ANNEX 2:

MEDICATION RE-LABELLING PROCEDURE FOR CLINICAL TRIAL USE QoLKAMON STUDY

Attached as a separate document

ANNEX 3:

QUALITY OF LIFE, TREATMENT ADHERENCE, AND TREATMENT SATISFACTION QUESTIONNAIRES

QUALITY OF LIFE ASSESSMENT QUESTIONNAIRE

MOS-HIV Health Survey

INSTRUCTIONS FOR STUDY COORDINATOR:

The following questionnaires ask patients about many aspects of their health state and health care. Questionnaires should be administered to patients before the physical examination, preferably in a quiet environment (e.g. the examination room or other room).

It is important to be familiar with the content and format of questionnaires before they are given to the study participants. At the first visit, instruct the patient as follows:

> "We would like you to answer some questions about how you feel and what you are able to do. Your answers will help us understand the effects of the medication you are taking. We would appreciate it if you would complete these questionnaires."

You should then briefly explain the format of questions and how to complete them. The patient must complete the questionnaires before the clinical history and physical examination are made and the vital signs are measured.

Questionnaires are very short and should take no more than 15-30 minutes to be completed. Before giving the questionnaires to the patient, fill in the header and DETACH THIS PAGE.

Collect the completed questionnaires before the physical examination. Before continuing, review the questionnaires for potential omissions. If the participant has not answered any question, point this to him/her and have him/her complete the omissions.

PLEASE COMPLETE THE FOLLOWING ITEMS AFTER THE PATIENT HAS COMPLETED THE QUESTIONNAIRES, OR AFTER YOU HAVE VERIFIED THAT THIS IS NOT POSSIBLE:

1. How were the questionnaires completed?...................... 1- They were self-administered by the study participant
   2- In a personal interview with you
   3- By a telephone interview
   4- They were not completed
   5- Other

   If the answer is Other, please specify ______________________

2. If the answer is 2 or 4, state the reason (1 = Yes, 2 = No)

   Initial patient refusal .... ☐

   Inadequate reading level of patient...................................... ☐

   Not enough time.. ☐

   Patient did not have his/her glasses .... ☐

   Other reason... ☐

   If the answer is Other reason, please specify ______________________

MOS-HIV Health Survey (continued)

PATIENT INSTRUCTIONS: **Please answer the following questions by checking the relevant box.**

1. Overall, would you say your health state is: (Check one answer only)

Excellent	1 ☐
Very good	2 ☐
Good	3 ☐
Fair	4 ☐
Poor	5 ☐

2. Overall, how much bodily pain have you experienced during the past 4 weeks? (Check one answer only)

None	1 ☐
Very mild	2 ☐
Mild	3 ☐
Moderate	4 ☐
Severe	5 ☐
Very severe	6 ☐

3. Over the past 4 weeks, how much did pain interfere with your normal work (or your normal activities, including work outside the home and housework)? (Check one answer only)

None at all	1 ☐
A little bit	2 ☐
Moderately	3 ☐
Quite a bit	4 ☐
Extremely	5 ☐

MOS-HIV Health Survey (continued)

4. The following questions refer to activities you might do during a normal day. Does your current health limit you for these activities? If yes, how much?

**(Check one box only on each line)**	**Yes, it limits me a lot (1)**	**Yes, it limits me a little (2)**	**NO, it does not limit me at all (3)**
a. Vigorous activities, such as lifting heavy objects, running or participating in sports requiring strenuous effort	☐ (1)	☐ (2)	☐ (3)
b. Moderate activities such as moving a table, carrying a shopping bag or bowling	☐ (1)	☐ (2)	☐ (3)
c. Walking uphill or climbing (several flights of stairs)	☐ (1)	☐ (2)	☐ (3)
d. Bending, lifting or stooping	☐ (1)	☐ (2)	☐ (3)
e. Walking one block	☐ (1)	☐ (2)	☐ (3)
f. Eating, dressing, bathing or using the toilet	☐ (1)	☐ (2)	☐ (3)

5. Does your health state keep you from working, doing household chores or going to school?

(Check one answer only)

Yes 1 ☐
No 2 ☐

6. Have you been unable to do certain kinds or amounts of work, housework or schoolwork because of your health state?

(Check one answer only)

Yes 1 ☐
No 2 ☐

MOS-HIV Health Survey (continued)

For each of the following questions, check the box of the answer <u>best reflecting</u> how you have been feeling <u>during the past 4 weeks</u>.

	All of the time<br>1	Most of the time<br>2	A good bit of the time<br>3	Some of the time<br>4	A little of the time<br>5	None of the time<br>6
7. During the past 4 weeks, how much of the time has your health state limited your social activities (like visiting friends or relatives)?	☐	☐	☐	☐	☐	☐
8. During the past 4 weeks, how much of the time:						
a. How you been very nervous?	☐	☐	☐	☐	☐	☐
b. Have you felt calm and peaceful?	☐	☐	☐	☐	☐	☐
c. Have you felt sad and downhearted?	☐	☐	☐	☐	☐	☐
d. Have you been a happy person?	☐	☐	☐	☐	☐	☐
e. Have you felt so down in the dumps that nothing could cheer you up?	☐	☐	☐	☐	☐	☐

MOS-HIV Health Survey (continued)

For each of the following questions, check the box of the answer <u>best reflecting</u> how you have been feeling <u>during the past 4 weeks</u>.

	All of the time<br>1	Most of the time<br>2	A good bit of the time<br>3	Some of the time<br>4	A little of the time<br>5	None of the time<br>6
9. During the past 4 weeks, how often:						
a. Did you feel full of pep?	☐	☐	☐	☐	☐	☐
b. Did you feel worn out?	☐	☐	☐	☐	☐	☐
c. Did you feel tired?	☐	☐	☐	☐	☐	☐
d. Did you have enough energy to do what you wanted to do?	☐	☐	☐	☐	☐	☐
e. Did you feel weighed down by your health problems?	☐	☐	☐	☐	☐	☐
f. Did you feel discouraged by your health problems?	☐	☐	☐	☐	☐	☐
g. Did you feel despair over your health problems?	☐	☐	☐	☐	☐	☐
h. Were you afraid because of your health state?	☐	☐	☐	☐	☐	☐

MOS-HIV Health Survey (continued)

	All of the time 1	Most of the time 2	A good bit of the time 3	Some of the time 4	A little of the time 5	None of the time 6
10. During the past 4 weeks, how much of the time:						
a. Did you have difficulties for reasoning and solving problems, for example, making plans, making decisions, learning new things?	☐	☐	☐	☐	☐	☐
b. Did you forget things that happened recently, such as were you put things and the appointments you had?	☐	☐	☐	☐	☐	☐
c. Did you have difficulties for keeping your attention on any activity for a long period?	☐	☐	☐	☐	☐	☐
d. Did you have difficulties doing activities requiring concentration and thinking?	☐	☐	☐	☐	☐	☐

11. Please check the box that best describes whether each of the following statements is true or false for you (Check one box on each line)

	Definitely true 1	Mostly true 2	Not sure 3	Mostly false 4	Definitely false 5
a. I am somewhat ill	☐	☐	☐	☐	☐
b. I am as healthy as anybody I know	☐	☐	☐	☐	☐
c. My health is excellent	☐	☐	☐	☐	☐
d. I have been feeling bad lately	☐	☐	☐	☐	☐

MOS-HIV Health Survey (continued)

12. How has your quality of life been during the past 4 weeks? That is, how have things been going for you? (Check one answer only)

Very well; could hardly be better	1	☐
Pretty good	2	☐
Good and bad parts about equal	3	☐
Pretty bad	4	☐
Very bad; could hardly be worse	5	☐

13. How would you rate your physical health and emotional status now as compared to 4 weeks ago? (Check only one answer)

Much better	1	☐
A little better	2	☐
About the same	3	☐
A little worse	4	☐
Much worse	5	☐

THANK YOU VERY MUCH FOR YOUR COOPERATION

EQ-5D Health Questionnaire

Translated from the Spanish Version

Check the answer in each section that best describes your health state TODAY.

Mobility

I have no problems for walking ❑
I have some problems for walking ❑
I have to stay in bed ❑

Self-care

I have no problems with self-care ❑
I have some problems for washing or dressing myself ❑
I am unable to wash or dress myself ❑

Usual activities *(e.g. work, school, housework, family activities, or leisure activities)*

I have no problems for doing my usual activities ❑
I have some problems for doing my usual activities ❑
I am unable to do my usual activities ❑

Pain/Discomfort

I have no pain or discomfort ❑
I have moderate pain or discomfort ❑
I have much pain or discomfort ❑

Anxiety/Depression

I am not anxious or depressed ❑
I am moderately anxious or depressed ❑
I am very anxious or depressed ❑

To help people describe how good or bad is their state of health, we have drawn a scale similar to a thermometer where the best state of health that can be imagined is marked 100 and the worst state of health that can be imagined is marked 0.

We would like you to indicate us in this scale how good or bad is, in your opinion, your state of health TODAY. Please draw a line from the box that says "Your health state today" to the point in the thermometer that indicates how good or bad is in your opinion your state of health TODAY.

The best state
of health
imaginable

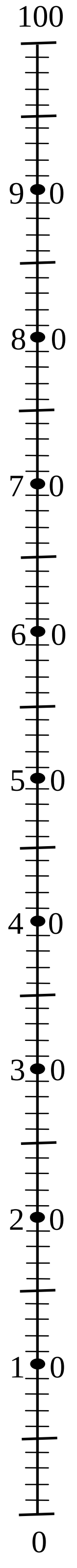

Your state of health today

The worst state
of health
imaginable

CESTA Questionnaire: SATISFACTION WITH HAART

Please answer all questions by checking the answer that best reflects your opinion about the antiretroviral treatment you are currently receiving.

1. With regard to the antiretroviral treatment you are currently taking: how satisfied have you been with your general state of health with the treatment you are receiving?

	Very satisfied
	Quite satisfied
	Regular
	Quite dissatisfied
	Very dissatisfied

2. With regard to the antiretroviral treatment you are currently taking: how satisfied how you been with the control of your disease produced by the treatment you are receiving?

	Very satisfied
	Quite satisfied
	Regular
	Quite dissatisfied
	Very dissatisfied

3. With regard to the antiretroviral treatment you are currently taking: how satisfied how you been with the absence of side effects or discomfort associated to the treatment?

	Very satisfied
	Quite satisfied
	Regular
	Quite dissatisfied
	Very dissatisfied

4. With regard to the antiretroviral treatment you are currently taking: how satisfied how you been with the amount of pills you take daily?

	Very satisfied
	Quite satisfied
	Regular
	Quite dissatisfied
	Very dissatisfied

5. With regard to the antiretroviral treatment you are currently taking: how satisfied how you been with the number of times you need to take your treatment daily?

	Very satisfied
	Quite satisfied
	Regular
	Quite dissatisfied
	Very dissatisfied

6. With regard to the antiretroviral treatment you are currently taking: how satisfied how you been with the changes or restrictions in your diet caused by the treatment you are taking?

	Very satisfied
	Quite satisfied
	Regular
	Quite dissatisfied
	Very dissatisfied

7. In general, are you satisfied with the antiretroviral treatment you are currently receiving?

	Very satisfied
	Quite satisfied
	Regular
	Quite dissatisfied
	Very dissatisfied

8. How important are the following aspects for you to feel satisfied with a given treatment? Check the relevant box for each of the aspects.

	Not important at all 0	Somewhat important 1	Fairly important 2	Quite important 3	Very important 4
Control of disease					
Side effects					
Number of daily doses (1, 2 or more daily doses)					
Number of pills you take					
Change or restriction in diet					

References:
Condes E, et al. Validación del cuestionario de satisfacción con el tratamiento antirretroviral: cuestionario CESTA Enferm Infecc Microbiol Clin 2005;23(10):586-92

Monitoring of treatment compliance

GEEMA Structured Questionnaire

Do you ever forget to take your medicines?	YES □ NO □
Do you take your medicines at the instructed time?	YES □ NO □
If you ever feel ill, do you stop taking the medication?	YES □ NO □
In the past week, how many times have you missed your medication?	
0 □ 1-2 □ 3-5 □ 6-10 □	More than 10 □
Have you ever missed your medication during weekends?	YES □ NO □
How many full days have you missed your medication since your last visit?	______ days
Why did you not take your medication?	

- □ Forgetfulness
- □ Adverse effect
- □ Timing difficulties
- □ Holidays or weekend trips
- □ Confusing doctor instructions
- □ None
- □ Other __________________________

Knobel H, Alonso J et al. Validation of a simplified medication adherence questionnaire in a large cohort of HIV-infected patients: the GEEMA Study. *AIDS 2002; 16: 605-613.]*

The best
adherence
imaginable

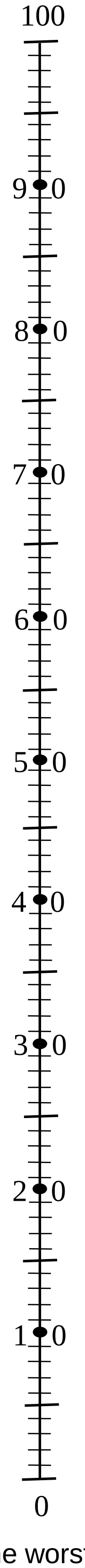

Your treatment adherence in the past month

The worst
adherence
imaginable
